# Short-term memory of past caspase activity biases cell elimination in vivo

**DOI:** 10.1038/s41467-026-77685-9

**Published:** 2026-09-22

**Authors:** Tom Cumming, Alexis Villars, Anđela Davidović, Florence Levillayer, Léo Valon, Romain Levayer

**Affiliations:** 1 Institut Pasteur, Université Paris Cité, CNRS UMR 3738, Cell Death and Epithelial Homeostasis Unit, Paris, France; 2 Institut Pasteur, Université Paris Cité, Bioinformatics and Biostatistics Hub, Paris, France; 3 Present Address: Department of Biosystems Science and Engineering, ETH Zürich, Basel, Switzerland

**Keywords:** Apoptosis, Single-cell imaging, Morphogenesis

## Abstract

Apoptosis is triggered by the activation of Caspases leading to irreversible engagement in cell death. However, recent data outline numerous non-apoptotic functions of caspases as well as quite ubiquitous sublethal activation of effector caspases in vivo, opening the question of what drives the bifurcation between cell death and survival. Using quantitative live imaging combined with machine learning and optogenetics in the *Drosophila* pupal notum (a single-layer epithelium), we reveal the existence of a large heterogeneity of caspase activity thresholds between cells and distinct spatial domains with low or high sensitivity to caspases. We outline the central role of past exposure to sublethal caspase activity, which sensitises cells to apoptosis for several hours and reduces their threshold, and can bias single-cell elimination. Altogether, this work reveals the role of a cell's past history for apoptosis regulation in vivo, which can bias clonal selection and the spatial pattern of cell death.

## Abstract (web summary)

Quantitative live imaging, machine learning and optogenetics in the *Drosophila* pupal thorax epithelium reveal the influence of past exposure to sublethal caspase activation, which sensitises cells for apoptosis for several hours and can bias cell elimination.

## Introduction

Apoptosis, the most prevalent form of programmed cell death, is essential to modulate tissue shape and size and contributes to developmental robustness^1,2^. This is particularly relevant in epithelia, which form a tight mechano-chemical barrier through cell-cell adhesion, and can undergo massive remodelling during development or adult organ homoeostasis^3^. While the molecular regulators of apoptosis are very well known, and all converge upon the cleavage and activation of the caspase proteases and the subsequent cleavage of thousands of proteins^4^, we still have a relatively limited understanding of apoptosis regulation in a multicellular and tissular context. This is epitomised by our inability to predict precisely when, where, how many and which specific cells will die in an epithelial tissue, most likely revealing the complex multilayered regulation of epithelial cell death^5^. Nonetheless, this level of predictivity appears essential when it comes to understanding the evolution of tissue size and shape, or anticipating clonal evolution during development or tumour progression^6^.

The activation of the most terminal caspases, so-called effector/executioner caspases, was long thought to constitute an irreversible point of engagement in cell death^7–12^. As a result, most molecular dissections of apoptosis regulation have focused on identifying factors acting upstream of caspase cleavage. However, studies over the past decade have now characterised an ever-increasing number of unexpected effector caspase functions which are unrelated to programmed cell death^13–15^. Alternatively, quantitative studies in cell culture revealed early on the variability of cell response to cytotoxic stress, so-called fractional killing^16^, where heterogeneity and noise related to cell protein content can alter the engagement in apoptosis despite similar upstream pro-apoptotic cues^17–19^. Similarly, mitochondrial outer membrane permeabilisation (MOMP), which was long thought to be an all-or-nothing process systematically leading to apoptosis^7,20^, was shown to be more nuanced due to the occurrence of partial MOMP that does not lead to cell death both in vivo^21–23^ and in cell culture^24,25^. Previous works in the *Drosophila* larval eye disc^26^ and in the pupal notum^27^ highlighted the role of modulation of the protein levels of Diap1, an inhibitor of apoptosis, which can tune cell sensitivity to apoptosis. The development of novel genetic sensors of effector caspase activation revealed quite systematic exposure of cells to sub-lethal effector caspase activity in vivo in *Drosophila*^28,29^ and in cancer cell lines^30,31^. These works clearly question the previously dominant view of caspase activation as the terminal point of engagement in apoptosis, and suggest that a decision process takes place downstream of effector caspase activation. Several alternative models have been proposed to redirect caspases to alternative functions, including the sequestration of active caspases to subcellular compartments^32,33^, or more generally the existence of a well-defined threshold of activity below which the cell will not enter apoptosis^34^. Accordingly, genetic evidence in *Drosophila* using a combination of caspase mutants and irradiation suggested that the susceptibility to engage in apoptosis could be set by procaspase levels^34^. However, more recent data combining single-cell quantification of caspase activity using a live sensor and optogenetic control of caspase found little correlation between caspase activity and the probability to enter apoptosis in HeLa cells^35^. Thus, what determines the decision between survival and death following effector caspase activation remains largely unclear, especially in vivo.

**Fig. 1:**
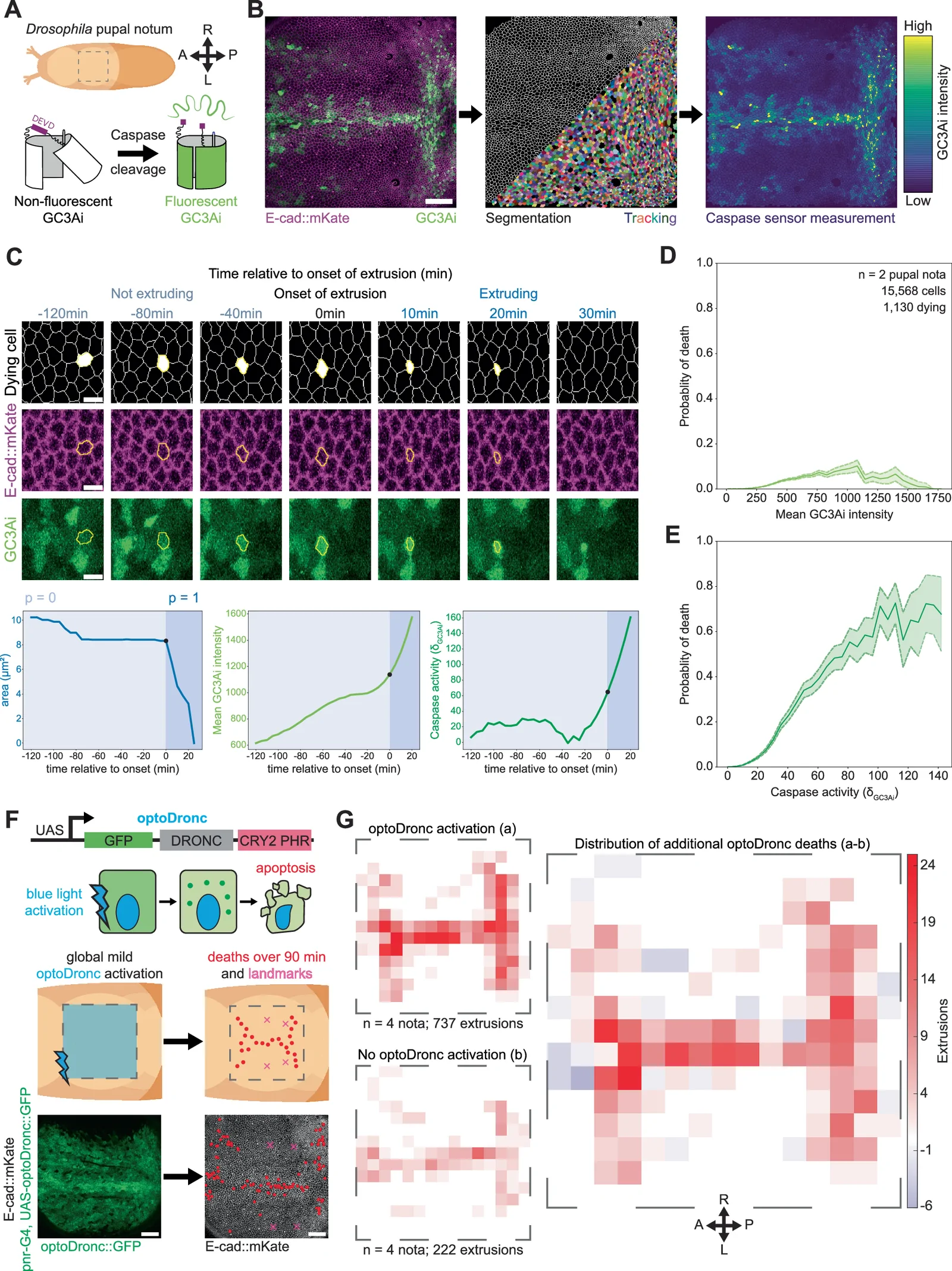
Cell-to-cell and spatial heterogeneity of caspase threshold associated with cell death (associated with Supplementary Figs. 1, 2 and 3). **A** Schematic of the *Drosophila* pupal notum (dotted square) and the GC3Ai effector caspase sensor. A: Anterior, P: Posterior, L: Left, R: Right. **B** Local z-projection of a live pupal notum expressing E-cad::mKate (magenta) and UAS-GC3Ai (green) in the pnr domain. Middle panel: cell contour upon segmentation, curation and cell tracking. Right panel: GC3Ai intensity measurement in single cells (blue: low, yellow: high). Scale bar=50 μm. **C** Snapshots of a dying cell before (left, negative time) and after the onset of extrusion (right, positive time), E-cad::mKate in magenta and GC3Ai in green. Yellow polygon: cell contour. Scale bars = 5 μm. Bottom graphs: corresponding apical area (left), GC3Ai intensity (middle) and estimated caspase activity (right, based on the GC3Ai intensity derivative). Dark blue zone: time when the cell is engaged in extrusion (after the area inflection point, black dots). Probability to be engaged in cell death (dark blue zone of the curves in **C**) as a function of the current GC3Ai intensity (**D**), or current effector caspase activity (GC3Ai intensity derivative) (**E**). *n* = 2 fully segmented pupal nota, 15,568 cells and 1130 dying cells. Thick lines: estimated probability, light colour areas: 95% confidence interval. **F** opto-Dronc construct, an optogenetic Caspase-9^40^, blue light-induced CRY2-PHR oligomerisation (green dots, middle scheme) induces Dronc cleavage. Middle scheme: zone of mild activation of optoDronc (blue) and pattern of cell death (red dots). Pink crosses show the macrochaetae used as landmarks for tissue alignment and scaling (see **Methods**). Bottom left: expression pattern of optoDronc::GFP driven by pnr-Gal4 (representative of *n* = 4 pupae). Bottom right: one example (representative of *n* = 4 pupae) of cell death distribution upon optoDronc mild activation (red dots, E-cad::mKate, grey). Scale bars=50 μm. **G** Summed map of the extrusions in nota over 90 min following mild optoDronc activation, *n* = 4 nota aligned and scaled, 737 extrusions. Bottom map: extrusions in control nota (same genotype, no blue pulse, 222 extrusions, 4 nota). Right map: distribution of additional cell death triggered by optoDronc (map (a) minus map (b)). The total local number of deaths is colour-coded (red high, blue low). A: Anterior, P: Posterior, L: Left, R: Right.

The *Drosophila* pupal notum, a single-layer epithelium, is an ideal system to study the quantitative regulation of apoptosis in epithelia in vivo. During metamorphosis, close to a thousand cells die in less than 24 hours in a highly stereotyped global pattern^36–41^, which can be visualised through long-term live imaging while combined with genetic modulation (Fig. 1A). Effector caspase activation systematically precedes and is required for cell extrusion toward the basal side^37,39–43^, which is in turn systematically followed by DNA compaction and apoptosis^41^. Using various live sensors of effector caspases, we previously outlined the large proportion of cells undergoing transient effector caspase activation without committing to cell extrusion and apoptosis^37,39,40^, which is also related to the reversion of caspase activity triggered by local pulses of EGFR/ERK activation near extruding cells^40^.

Here, we use the pupal notum to quantitatively characterise in vivo the relationship between effector caspase activity and commitment to apoptosis. Using a live sensor of effector caspases and a large dataset of tracked cells, we first confirmed that caspase activity provides good predictive power regarding the likelihood of cell death using machine learning (time series forest). However, we surprisingly found that cells commit to apoptosis at various levels of effector caspase activity. Using optogenetic sublethal activation of caspases, we also outlined the reproducible global tissue pattern of caspase sensitivity in the notum, thus confirming that commitment to apoptosis downstream of caspases can be developmentally regulated. Using correlative analysis combined with quantitative optogenetic perturbations, we revealed that past exposure to caspase can prime cells for apoptosis a few hours later. This contribution of past exposure can explain a significant proportion of the global spatial heterogeneity of caspase sensitivity and is sufficient to predict which cells will commit to apoptosis following a pulse of caspase activation. Finally, using machine learning and clonal perturbation, we confirm that past exposure to caspases can bias cell elimination relative to neighbouring cells and modulate clonal selection during development, hence describing an original mode of physiological cell competition.

## Results

### Spatial and cell-cell heterogeneity of caspase thresholds

We first characterised the effector caspase activity levels associated with engagement in apoptosis in the pupal notum. We segmented and tracked more than 15,000 cells using deep learning pipelines^44^ and a new plugin for segmentation curation and tracking^45^ to measure the temporal dynamics of caspase activity at the single-cell level using the GFP-based effector caspase sensor GC3Ai^46,47^ (Fig. 1A, B, Supplementary Movie 1). Given the stability of activated GC3Ai^47^, we used the intensity derivative (the rate of production of active GC3Ai) as a proxy for effector caspase activity^40^ (called “caspase activity” hereafter). Engagement in cell extrusion is systematically followed by DNA compaction and apoptosis^41^. We therefore used the initiation of extrusion as a proxy for engagement in apoptosis and systematically detected the onset of area reduction in extruding cells to define cell death engagement (see Methods and^42^, Fig. 1C, Supplementary Movie 2). We first used machine learning to identify relevant single-cell parameters predictive of engagement in apoptosis. To avoid bias coming from unbalanced data and the impact of global tissue patterning on cell shape and behaviour, we trained the models systematically using one dying cell and one of its direct surviving neighbours as a control. To integrate temporal features (mean, standard deviation, derivative), we used a time series forest classifier (TSF^48^, see **Methods**) on backward temporal windows of one hour starting at least 10 min prior to the onset of extrusion. We first confirmed that cell apical area and perimeter could provide a good prediction of cell death engagement prior to the onset of extrusion (Supplementary Fig. 1A, C, D), as previously demonstrated^41^. While cell eccentricity had little predictive value (Supplementary Fig. 1B), we found however, that caspase activity could accurately predict cell death (Supplementary Fig. 1E, F), especially in the hour preceding the initiation of extrusion. This suggested that instantaneous caspase activity is relevant to predict the engagement in apoptosis and that the decision to die or survive is taken close to the onset of cell extrusion. We therefore explored more extensively the quantitative behaviour of caspase activity close to the cell death engagement point.

We systematically measured the levels of caspase activity before and after the onset of cell extrusion in dying cells (Supplementary Fig 2A, B), as well as measuring caspase activity in non-dying cells (Supplementary Fig. 2D, E). Here, we define ‘caspase threshold’ as the caspase activity value at which individual cells engage in cell extrusion. Doing so, we obtained the probability of being in apoptosis for a given level of cumulative caspase activity (absolute GC3Ai intensity) or instantaneous caspase activity (derivative of GC3Ai signal, Fig. 1D, E). We first found that absolute GC3Ai signal on its own provides little predictive information on engagement in cell death, as exemplified by the large proportion of living cells with high intensity levels (Fig. 1D, Supplementary Fig. 1G, H, logistic fit Tjur’s R^2^ = 0.016) and as previously reported in HeLa cells^35^. We observed however a good logistic fit when plotting the proportion of cells engaged in apoptosis as a function of caspase activity (Fig. 1E, Supplementary Fig. 1G, I logistic fit Tjur’s R^2^ = 0.301). While this result suggests that effector caspase activity is partially predictive of cell death engagement, it also outlines the significant cell-to-cell variability in the caspase threshold associated with commitment to apoptosis/extrusion, as the range of caspase activity levels at which cells engage in apoptosis spans nearly two orders of magnitude (Fig. 1E, see Supplementary Fig. 2C for the distribution of caspase activity values at which cells engage in extrusion). We confirmed that the variability of caspase threshold does not correlate with cell-to-cell heterogeneity of Gal4 expression levels, as there was no correlation between caspase activity at the onset of extrusion and level of expression of another UAS-driven fluorescent sensor (Supplementary Fig 1J, K, Spearman correlation r = −0.124, *p* = 0.58).

We then tested whether this cell-to-cell heterogeneity in death-inducing caspase threshold had any developmental relevance. To assess the variability of caspase sensitivity in space, we used an optogenetic initiator caspase previously developed by our group (optoDronc^40^). optoDronc can be used to quantitatively modulate the levels of caspase activation and the proportion of dying cells by modulating blue light exposure time (Supplementary Fig. 3A–C). We therefore used global and short blue light exposure to trigger mild and uniform activation of caspases in a large band encompassing most of the notum (using the pnr-Gal4 driver, Fig. 1F). We then systematically tracked the occurrence of extrusion in the domain expressing optoDronc, and registered the position of macrochaetae (large bristles) from each mapped notum as spatial landmarks for a Procrustes transformation, allowing us to scale and align death events from several movies to a single spatial map (see **Methods** and Supplementary Fig. 3D). The same procedure was applied to pupae with the same genotype in the absence of blue light pulses as controls. We first revealed that the vast majority of additional deaths occur in the 90 min following mild activation of optoDronc (Supplementary Fig. 3E, Supplementary Movie 3). We therefore created maps of cell death using extrusions occurring in the first 90 min following blue light pulses. We subtracted the distribution of spontaneous death in the control map from the blue light-stimulated map to obtain the spatial distribution of additional deaths triggered by the activation of optoDronc (Fig. 1G, Supplementary Movie 3). This revealed a clear heterogenous pattern of death induction, with a higher susceptibility to caspase in the midline and in bands at the anterior and posterior edges of the tissue (Fig. 1G, Supplementary Movie 3). This heterogeneity is not explained by spatial variability of the driver (Fig. 1F**bottom**), nor by differences in the kinetics of effector caspase activity downstream of optoDronc (no spatial difference of GC3Ai dynamics upon prolonged blue light activation, Supplementary Fig. 3F, G, Supplementary Movie 4). Moreover, the comparison of the cumulative number of deaths over 3 h in the controls and the nota exposed to blue light clearly shows that optoDronc pulses trigger a net increase in the number of cell deaths (Supplementary Fig. 3E, 956 versus 482 extrusions, 4 pupae each). This confirmed that the pattern we observed shows “additional” death events and not a simple “acceleration” of the elimination of cells that would have died during normal development. Thus, we revealed a stereotypical spatial pattern of caspase sensitivity, which suggests that the engagement in apoptosis downstream of effector caspases can be developmentally regulated.

### Past caspase activity primes cells for apoptosis

**Fig. 2:**
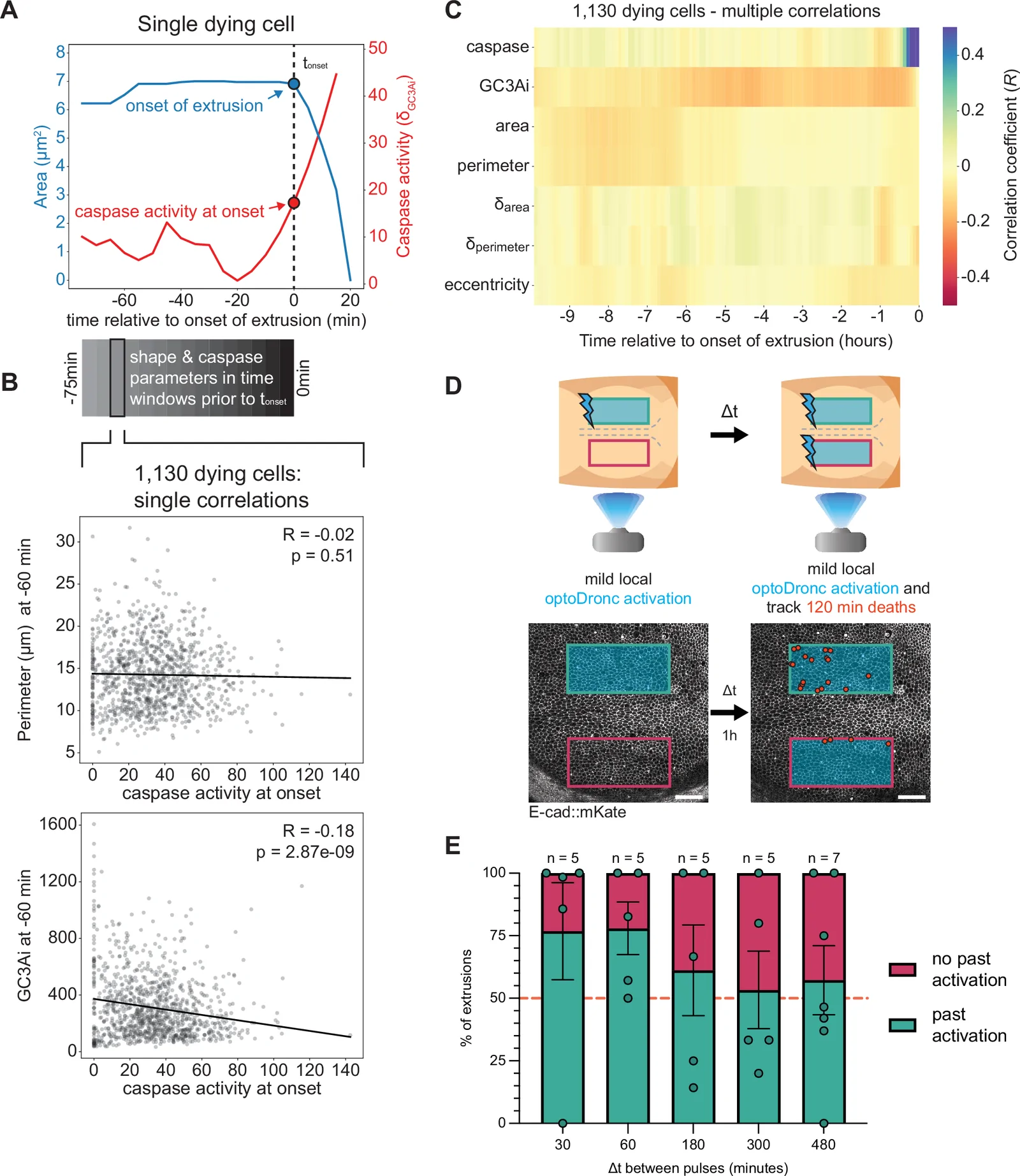
Past caspase activation primes cells for apoptosis (associated with Supplementary Fig. 4). **A** Cell area (blue) and caspase activity (GC3Ai intensity derivative, red) of a single dying cell. Black dotted line: onset of cell extrusion. Blue and red dots: area and caspase activity at the onset of apoptosis engagement. Multiple parameters were tracked backward in time (grey squares, 20 min sliding window shifted every 5 min) related to cell geometry and caspase on 1130 dying cells (2 nota). **B** Examples of Pearson correlations calculated between caspase activity at the onset of apoptosis engagement and perimeter (top) and GC3Ai intensity (bottom) 70 to 50 min prior to apoptosis engagement. *p*-values: Pearson correlation test, two-sided. **C** Matrix of correlations of geometrical (cell apical area, perimeter, rate of change of area, rate of change of perimeter, eccentricity) and caspase-related parameters (GC3Ai intensity, caspase: derivative of GC3Ai intensity) with caspase activity at the onset of apoptosis engagement. Correlations are calculated on 20 min time windows prior to the onset of cell extrusion (t = 0 h). Blue: positive correlation, Red: negative correlation. Note the clear global negative correlation between past GC3Ai intensity and caspase at the onset. *n* = 1130 dying cells, 2 nota. **D** Schematic of the memory experiment. Mild activation of *UAS-optoDonc* (*pnr-Gal4* driver) with a short blue light pulse in the lateral right part (top, regions with very limited spontaneous cell death). After different waiting times, the same region and the contralateral left region of the notum are exposed to blue light. The number of extrusions is then compared in the left (1 stimulation) and right region (2 stimulations) over 120 min. Bottom pictures: distribution of cell extrusions in the left and right regions over 120 min after the second light pulse on one notum (one red dot = one cell extrusion, grey, E-cad::mKate) Scale bars = 50 μm. **E** Proportion of cell extrusions in the right (twice-exposed) and left (once-exposed) regions, as a percentage of the total extrusions per notum over 120 min after the second blue light pulse, waiting time of 30, 60, 180, 300 or 480 min after the first blue light exposure (*n* = 5–7 nota per condition). One dot per notum. Error bars show s.e.m.

**Fig. 3:**
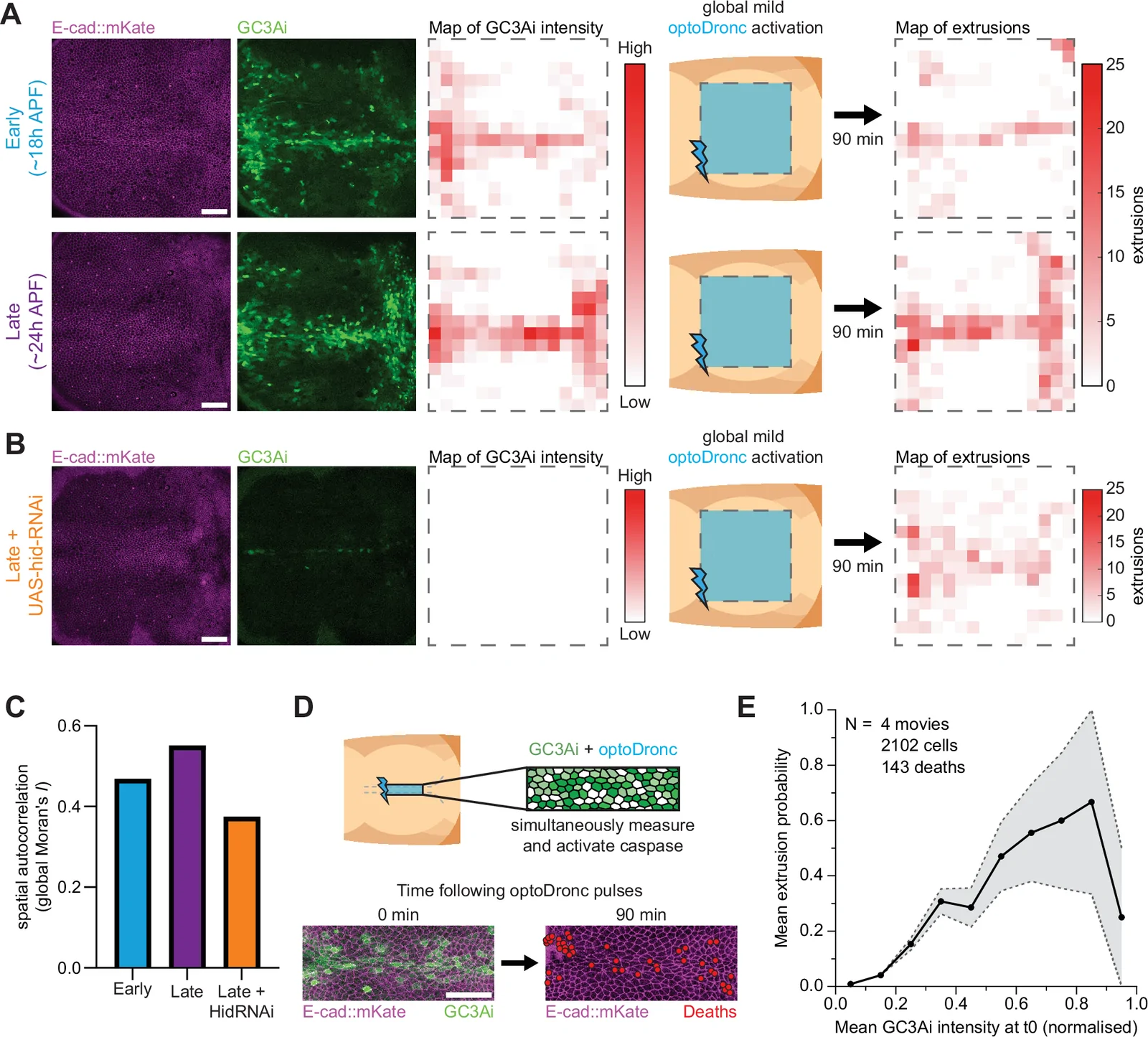
Past caspase activation is predictive of apoptosis spatial pattern and single-cell decision. Left, local projections of an early (top, ∼18 h APF), and late (middle, ∼24 h APF) pupal notum (**A**), or late pupal notum upon depletion of the pro-apoptotic gene *hid* by RNAi (bottom, **B**). E-cad::mKate (magenta), *pnr-Gal4 UAS-GC3Ai* (green, same intensity scale for all) and the spatial map of the GC3Ai intensity (right, white to dark red, same scale in the three conditions). Scale bars=50 μm. Middle: schematic of the global optoDronc activation performed and the tracking of cell death over 90 min. Right: Sum maps of cell death distribution observed in the 90 min following blue light pulse obtained after the rescaling/alignment of 3 young (top, **A**), 3 old (middle, **A**), and 3 old *UAS-hid dsRNA* pupae (bottom, **B**, note that hid dsRNA is triggered from earlier stage in the pnr domain), total extrusion number: 238 in the young, 644 in the old pupae, 345 in old *UAS-hid dsRNA* pupae. Colour scale (white to dark red, low to high number of extrusions) is the same in the 3 conditions. **C** Spatial autocorrelation of the extrusion pattern observed over 90 min after optoDronc activation in young, old, or *UAS-hid dsRNA* pupae (see **Methods**). **D** Top, schematic of the experiment: local activation of optoDronc in the midline is combined with the imaging of GC3Ai intensity at t0. Cell extrusion is then mapped in the next 90 min. Bottom, local projection of an early pupa at the onset of blue light exposure (GC3Ai signal, green, E-cad::mKate, magenta). The localisation of cell death in the next 90 min is shown with red dots. Scale bar = 50 μm. **E** Single cell probability of cell extrusion during the 90 min following optoDronc activation in the midline as a function of the normalised GC3Ai raw intensity at t0. Grey area is the standard error of the mean (note that probability measurement is poorly reliable above 0.7 due to the low number of cells showing these extreme values).

We next set out to identify parameters that could explain the heterogeneity of caspase sensitivity. We isolated the 1130 dying cells from our dataset, extracted the level of caspase activity at the onset of extrusion of each cell, and assessed the strength of the correlation between this value and various morphometric and caspase-related parameters in windows of time prior to extrusion (Fig. 2A, B, Supplementary Fig. 4). Doing so, we outlined an interesting negative correlation between GC3Ai intensity in the past (up to 4–5 h prior to extrusion) and caspase activity at the onset of extrusion (Fig. 2C). This suggested that previous exposure to caspase activity may decrease the instantaneous caspase threshold required to enter apoptosis, hence priming the cell for death. We therefore experimentally tested whether previous exposure to sublethal caspase activity could indeed prime cells for apoptosis. We used optoDronc to locally expose a subdomain of the notum (lateral to the midline where there is no physiological cell death^38,40,41^) to two pulses of caspase stimulation separated by 30 min, 1, 3, 5 or 8 h, and used as a control the contralateral region exposed only to the latter stimulation (Fig. 2D, Supplementary Movie 5). Note that the heminota should normally be perfectly symmetric in terms of cell death, as documented by the very symmetric pattern of cell death in physiological context^38,40^, and the symmetric pattern of additional death triggered by a global pulse of Dronc activation (Figs. 1G, 3A, B). We observed that cells were significantly more likely to die in the region with prior active caspase exposure compared to the single exposure region, with a more than 4-fold increase in cell death number after 30 min and 1 h, ~25% increase after 3 h, and no clear difference beyond 5 h (Fig. 2E, Supplementary Movie 5). This confirmed that past exposure to active caspase can indeed prime cells for apoptosis for a few hours.

### Integration of past caspase activity is predictive of the spatial pattern of death and single-cell engagement in apoptosis

So far our results suggest that past caspase activity provides relevant information to predict engagement in apoptosis upon further caspase activation. We therefore revisited our experiments triggering global mild caspase activation in the notum (Fig. 1F, G), and noticed that the pattern of apoptosis induction closely matched the pattern of absolute GC3Ai intensity (compare Supplementary Movie 1, Fig. 1B with Fig. 1F). We therefore checked whether the pattern of cumulative past caspase activity (using absolute GC3Ai intensity as a proxy) could indeed explain the preferential localisation of cell death following a pulse of optoDronc activation. The pattern of GC3Ai intensity changes during pupal development, with early pupae showing a strong signal in the anterior domain, mild signal in the midline and no signal in the posterior region (~18 h APF), while at late stage there is a strong increase in signal in the midline and the posterior region (~24 h APF, Supplementary Movie 1, Fig. 3A). We therefore used optoDronc to trigger mild global caspase activation in early and late pupae to compare the pattern of cell elimination and the pattern of GC3Ai intensity. Strikingly, caspase activation in the early pupae mostly triggers death in the anterior and midline domain and has little impact in the posterior region, while induction of caspase in late pupae triggered death in the anterior, midline and posterior regions, correlating well with the corresponding pattern of GC3Ai signal at the relevant developmental stage (Fig. 3A, Supplementary Movie 6, Pearson correlation comparing the spatial pattern of GC3Ai intensity and local extrusion number r = 0.31, *p* = 1.60.10^−6^ for early pupae and r = 0.65, *p* = 1.67.10^−28^ for late pupae). This suggested that the pattern of death induction by optoDronc may be largely explained by past exposure to caspase activation. To directly confirm the link between past activation and the spatial pattern of caspase sensitivity, we erased physiological caspase activity by depleting the pro-apoptotic gene *hid* using RNAi^39,42^ (see the near absence of GC3Ai signal Fig. 3B left, Supplementary Movie 7), and triggered a pulse of caspase activity in the *pnr* domain with optoDronc at late stage (Fig. 3B, Supplementary Movie 6). Epistatically, optoDronc can directly trigger caspase activation downstream of the pro-apoptotic genes *hid*, *grim* or *reaper*^5^ and bypass the depletion of *hid* by RNAi. We therefore characterised the pattern of death induction by optoDronc in the *hid* RNAi background. Interestingly, the pattern of cell elimination was much more homogeneous in the context of *hid* RNAi compared to optoDronc pulses in the WT background (Fig. 3B, compared to Fig. 3A). The diminution of spatial patterning is reflected by the considerable reduction in clustering of cell death distribution in the *hid* RNAi context compared to late controls (Fig. 3C, reduced global Moran’s *I*). This confirmed that past caspase activation (here regulated by *hid*) is essential to generate the spatial bias of cell elimination. We noticed, however, that there is still a slight increase in the probability to observe cell death near the midline and in the anterior domain; accordingly, the spatial autocorrelation is still higher than that expected from a random spatial distribution (Fig. 3C, global Moran’s *I* for a random distribution ≈ 0). This suggests that other unknown spatially patterned factors can also modulate the susceptibility to caspases in the notum independently of past caspase activity.

We then checked whether the history of cells and their previous exposure to caspase could also be predictive of the engagement in apoptosis at the single-cell level. Combining both optoDronc and GC3Ai under the expression of the notum-wide pnr driver, we used a pulse of blue light to simultaneously image the levels of GC3Ai at time 0 and globally activate caspase. We then assessed which cells would die in the midline, and found a striking correlation at the single-cell level between the probability to die and the levels of GC3Ai intensity at time 0 (Fig. 3D, E, 18 h APF pupae, Supplementary Movie 8). This suggests that cumulative past exposure to caspase is predictive of which cell will engage in apoptosis following a pulse of caspase activity. While we previously showed that absolute GC3Ai levels on their own are poorly predictive of apoptosis engagement (Fig. 1D), we show here that cumulative past caspase activation (GC3Ai levels) can significantly modulate cell elimination probability when caspase becomes active at a given time. Altogether, these results suggest that the decision to die is first dominated by the current status of caspase activity, which is then fine-tuned by cumulative past exposure.

### Relative past and current caspase activity can bias clonal elimination and the death probability relative to neighbours

**Fig. 4:**
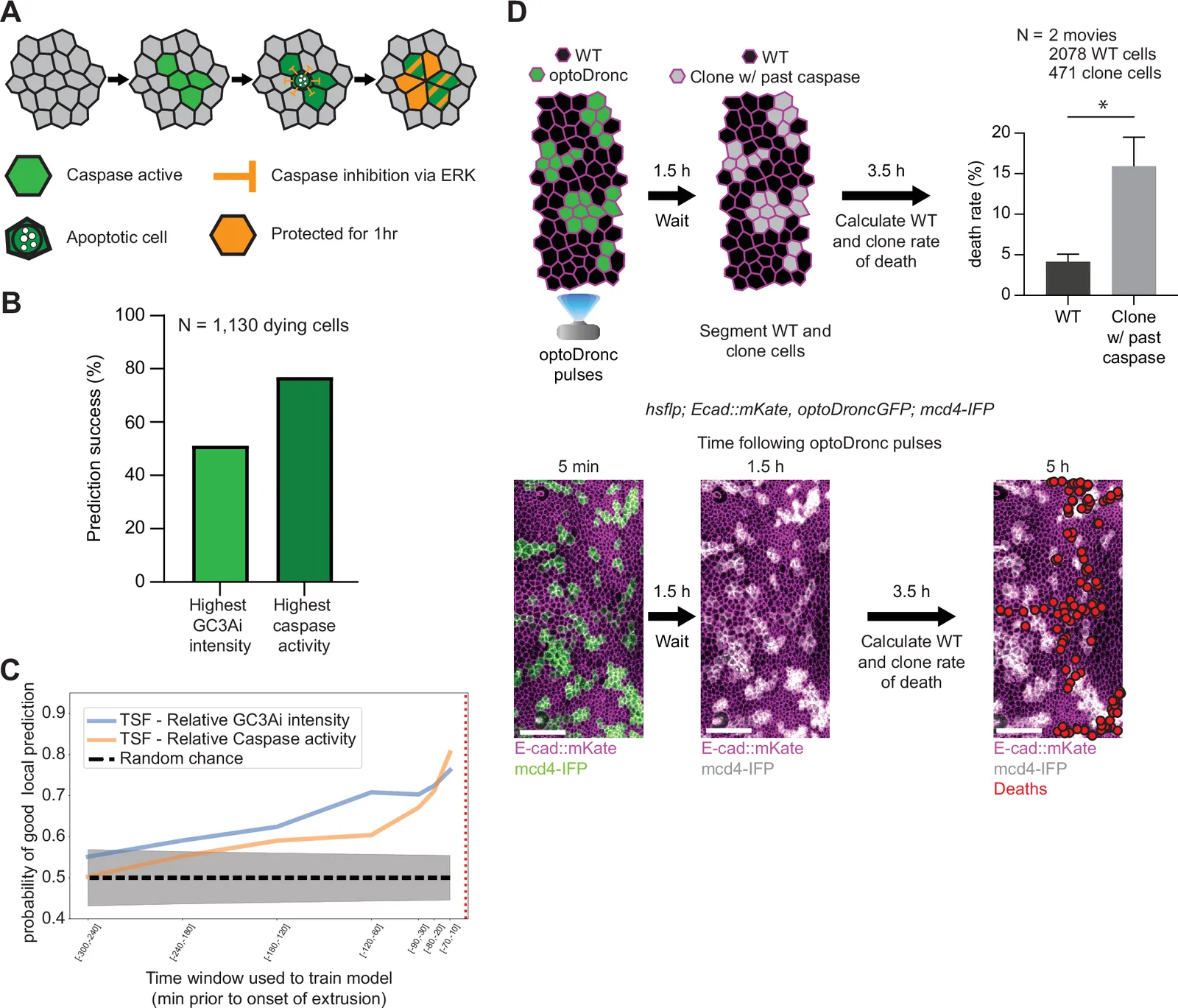
Past caspase activation biases single and clonal cell elimination. **A** Upon initiation of cell extrusion, activation of ERK in the neighbouring cells (orange) blocks caspase activity (green) and protect them from apoptosis for 1 h (see ref. ^40^). **B** Percentage of good prediction of which cell will die first among a group of neighbours. The first dying cell has the highest GC3Ai signal in 52% of cases, and the highest caspase activity in 77% of cases. *n* = 1130 groups of cells. **C** Proportion of correct prediction of the first cell dying among a group of neighbours based on a Time Series Forest model. The cell with the highest probability score in the group is predicted to be the first to die. TSF is trained either on relative GC3Ai intensity (blue) or on relative caspase activity (orange), comparing a dying cell and a surviving neighbour as control (see **Methods**). Black dotted line: expected score for random prediction; grey area: 95% confidence interval (prediction is tested on a balanced dataset of dying cells and surviving neighbours, random prediction is correct in 50% of cases). The time windows used for TSF prediction and training are shown on the x-axis (time in minutes prior to extrusion onset, starting at −10 min). **D** Clones expressing optoDronc (green) located in the posterior region of the pupal notum are exposed to a mild blue light pulse. The rate of death is then calculated in the clonal cells and outside the clones from 1.5 hrs to 5 hrs after blue light pulses. In this time window, cell death is mostly triggered by spontaneous developmental activation of caspase in the posterior region. Bottom panels: local projection of a pupal notum expressing E-cad::mKate (magenta) and UAS-mCD4::mIFP (green/grey) at the onset of blue light pulses, 1.5 hrs and 5 hrs later. Red dots: cell extrusion events counted from 1.5 hrs to 5 hrs. The death probability in control and optoDronc clones is shown in the bar graph (*n* = 471 clonal and 2078 WT cells, 2 nota, *p* = 2.196e^−17^, Fisher’s exact test, two-sided). Error bars are 95% confidence intervals around the estimated proportion of dying cells (cumulating all observed clonal and WT cells). Scale bars = 30 μm.

We previously showed that the initiation of cell extrusion triggers a pulse of ERK activity in the neighbouring cells, which shuts down caspase and protects them from apoptosis^40^. Accordingly, when neighbouring cells activate caspase simultaneously, the first cell to enter extrusion will inhibit the death of its neighbours^40^ (Fig. 4A), in a similar fashion to cell selection by lateral inhibition^49^. As a result, cell death decision-making is not fully cell autonomous and rather relies on which cells in the group will be the fastest to enter extrusion/apoptosis. We therefore tested whether relative differences in caspase activity and past exposure to caspase could bias the selection of the cell to die among neighbours. We compared dying cells and their neighbours and checked which parameter is predictive of the first cell to die. Among 1130 groups of individual dying cells and their neighbours, the first cell to die had the highest GC3Ai signal in 52% of cases (random assignment would be close to 15%) and had the highest caspase activity in 77% of cases (Fig. 4B, comparison at the onset of the first extrusion). This suggested that relative caspase activity and relative previous exposure to caspase could both bias cell elimination among neighbours. To confirm that our capacity to predict the engagement in death among a group of cells is not just restricted to the time point at the onset of extrusion, we again used TSF machine learning to test the predictive strength of relative differences in caspase between neighbouring cells over time. We first used the TSF model to assign a death score value for each cell in a group of neighbours, and assumed that the one with the relative highest score should be the one to die first (Fig. 4C. see **Methods**). We used the relative GC3Ai and caspase activity values between the dying cell and its neighbours for training the TSF model. This led to very good prediction of which cell will die first in a group when using the first hour window preceding cell extrusion onset with GC3Ai intensity or caspase activity (Fig. 4C, F1 scores>0.7, note that TSF of GC3Ai also includes information about the derivative), and stronger predictive capacity for raw GC3Ai starting 1 h prior to the onset of extrusion and lasting up to 3 h (Fig. 4C). Altogether, these results confirmed that the combination of the relative values of caspase activity and past exposure are sufficient to predict which cell will die first in a group of neighbours. They also confirmed that the instantaneous caspase activity close to the extrusion point remains the main driving factor, which is then modulated by previous caspase activation.

**Fig. 5:**
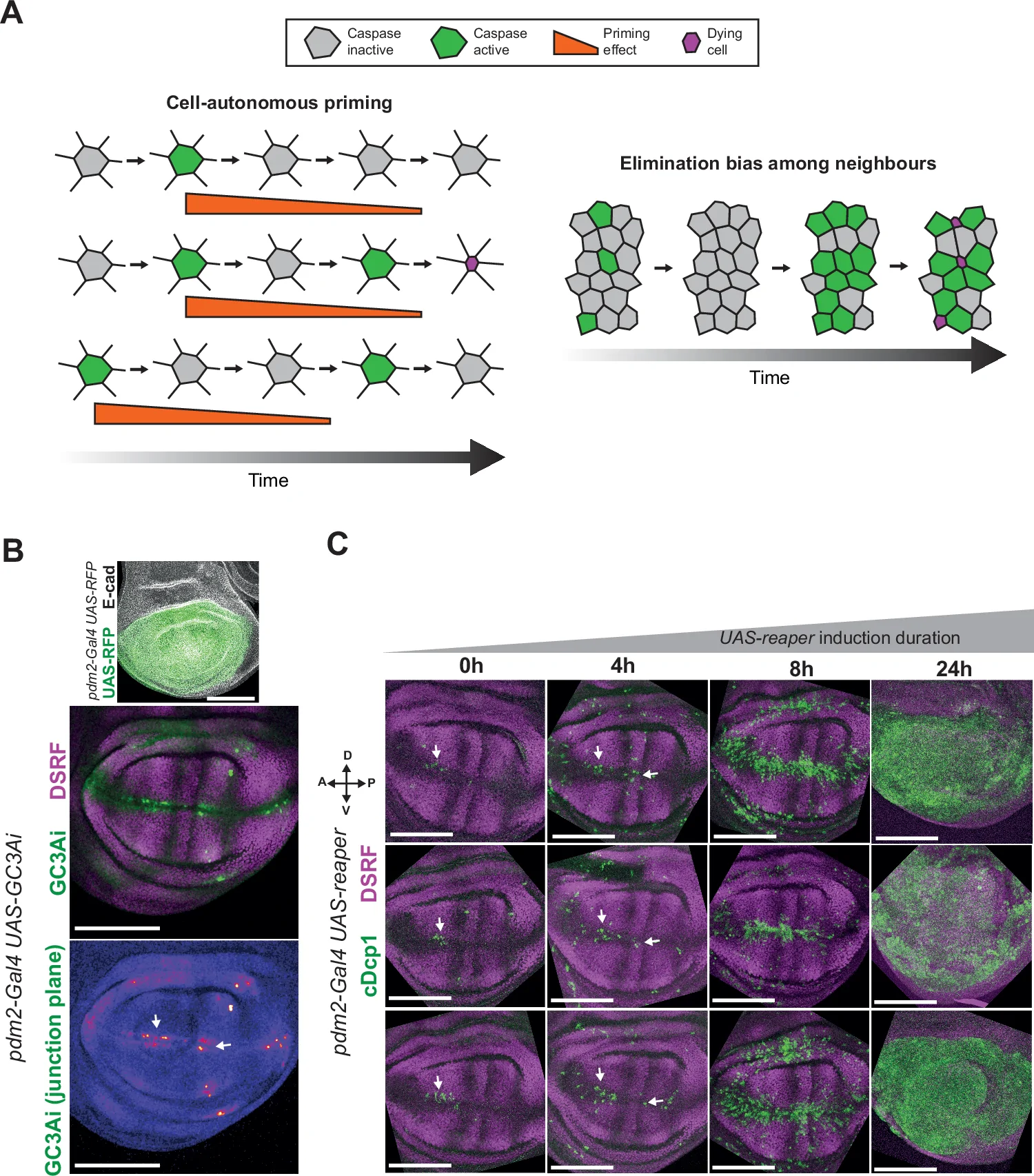
Working model and cross-validation in the larval wing imaginal disc (associated with Discussion). **A** Working model. Left: Cells exposed to a first pulse of caspase activity are primed for apoptosis for a few hours. If a second pulse occurs within this time window, the cell is more likely to die. Right: previous exposure to caspase activity can bias cell elimination among neighbours. The cells previously exposed are preferentially eliminated later despite global caspase activation. **B** Top, pattern of expression of the pdm2-Gal4 driver in the larval wing imaginal disc (green, UAS-RFP, grey, E-cad). Middle: local projection of DSRF staining (intervein regions, magenta), GC3Ai signal in the plane of adherens junctions (green). Bottom: Pseudo-colour of GC3Ai intensity in the adherens junction plane. White arrows show regions of high GC3Ai signal. Scale bars=100 μm. Representative of *n* = 29 wing discs, see ref. ^55^ for further characterisation. **C** Representative examples of cell death distribution (cleaved Dcp1, green) upon induction of Reaper in the *pdm2-Gal4* domain for different durations of activation (duration of 29 °C incubation in hours releasing Gal80^ts^ repression). Cell death is mostly located in the previously characterised apoptotic hotspots at 0 h (see ref. ^55^), is first enriched in regions already showing some cumulated GC3Ai signal after 4 and 8 h of activation, and eventually is distributed throughout the wing disc. *n* = 23, 24, 28 and 14 wing discs. Scale bars = 100 μm.

This non-cell autonomous death decision is reminiscent of cell competition^50,51^, a context-dependent cell elimination process which triggers the elimination of suboptimal cells by their neighbours through short-range communication. We therefore checked whether past exposure to caspase could indeed bias clonal selection during normal development. We triggered mild/sublethal activation of optoDronc in clones of the posterior domain of the notum and quantified the probability of cell death from 90 min to 5 h after blue light pulses (hence not counting deaths directly triggered by the first pulse of activation, see Supplementary Fig. 3E). We found a striking increase in death probability relative to neighbouring WT cells (Fig. 4D, Supplementary Movie 9, 3.5-fold increase of cell death probability), confirming that past exposure to caspase is sufficient to generate “loser”-like clones which are more likely to be eliminated in regions where caspases become physiologically activated later. Altogether, we showed that past exposure to caspase can bias single-cell elimination and clonal dynamics, suggesting that the history of caspase activation could be sufficient to generate physiological cell competition by priming a subset of cells for apoptosis (Fig. 5A). This mechanism of physiological cell competition would not require direct comparison between cells and could be sufficient to bias cell elimination.

## Discussion

We outlined here the large diversity and spatial heterogeneity of caspase sensitivity in vivo. The existence of reproducible large spatial patterns of caspase sensitivity suggests that the decision to engage in apoptosis downstream of effector caspases can be developmentally regulated. Using machine learning, optogenetics and correlative analysis, we demonstrated that past exposure to caspase activation can prime cells for apoptosis in the following hours. This “memory” of past activation explains a significant proportion of the spatial bias of caspase sensitivity and can predict which cell will engage in apoptosis following a pulse of caspase activation. Globally, our work demonstrates that while instantaneous caspase activity is the primary predictor of cell death engagement (Fig. 1E and Supplementary Fig. 1), information provided by past exposure generates a significant proportion of the heterogeneity that can bias cell elimination. Of note, we still found a significant spatial bias of cell death distribution upon optoDronc activation despite erasure of past caspase activity (Fig. 3B,C). This suggests that other parameters, yet to be identified, can also modulate the susceptibility to enter apoptosis following caspase activation in the notum. While our work outlined an important cell-to-cell heterogeneity of the caspase activity levels at which cells engage in apoptosis, it does not exclude the existence of a relevant single cell threshold for apoptosis induction. Moreover, our characterisation was based on a bulk sensor of caspase activity, and we cannot exclude that the apoptotic decision relies on a specific subcellular pool of active caspase where a well-defined deterministic threshold may still exist. Of note, we also find a slight inconsistency between the timescale revealed by correlation (up to 6 h, Fig. 2C) and the memory timescale revealed by optoDronc pulses (roughly 3 h, Fig. 2E). Subtle effects may be persisting beyond 5 h of delay and masked by the variability of the optoDronc experiment. Alternatively, the long-term correlation may be related to indirect effects (such as the probability to be exposed several times to caspase activity in a given domain of the tissue). Overall, these results match with a short-term memory of caspase activation lasting for a few hours.

Previously, quantitative characterisation of caspase dynamics in HeLa cells using the same GC3Ai sensor suggested that caspase activity was poorly predictive of apoptosis engagement^35^, in contrast with our system where the combination of present and past caspase activity is sufficient to predict cell death. This apparent discrepancy may be explained by various differences between the biological systems and the assays. For instance, we focus here on epithelial cells where cell death decision is intrinsically connected to cell extrusion. Moreover, while we can use the engagement in extrusion to temporally align dying cells, there is no trivial criteria to define the onset of apoptosis in HeLa cells and align the temporal profiles, which may have masked relevant information about the utility of caspase activity to predict cell death. Since our machine learning approach suggests that the information provided by caspase activity is mostly relevant in the few hours preceding cell extrusion, such temporal alignment is therefore essential to identify relevant signatures. Finally, the intrinsic resistance of HeLa cells to apoptosis^52–54^ may mask the instructive role of caspases. Accordingly, caspase signals become predictive in HeLa cells when they are primed by stress conditions^35^. Irrespective of these differences, these two studies clearly outline the variability of cell death decision-making following caspase activation and the existence of downstream parameters that can modulate apoptosis engagement. Of note, the influence of past caspase activity on the spatial pattern of caspase sensitivity does not seem to be a peculiarity of the pupal notum. Similar spatial bias can be observed in the wing imaginal disc upon homogeneous and transient pulses of pro-apoptotic genes, which preferentially trigger cell death in regions that were already physiologically exposed to caspases^55^ (Fig. 5B), suggesting that the same memory effect may exist in other tissues. This study relies on the relationship between engagement in extrusion and engagement in apoptosis. Interestingly, we observed a systematic exponential increase in caspase activity as individual cells progress in extrusion (Ref. ^40^ and Fig. 1C), which may be compatible with a positive feedback loop whereby extrusion remodelling promotes caspase activity, which then promotes extrusion progression. This amplification loop could explain the binary decision associated with extrusion and apoptosis engagement. Future work will help to identify the factors promoting this rapid increase of caspase activity and its relationship with the well-described process of anoikis, whereby loss of adhesion drives cells to irreversibly commit to die^56^.

What, then, are the molecular mechanisms encoding the memory of past caspase activation, and what sets this characteristic timescale of a few hours? This memory is unlikely to be explained by the perdurance of active caspases, which were previously shown to be very short-lived^9,57–59^, in agreement with our previous measurement of the short time delay between pulses of ERK and inflection of caspase activity (averaged delay of 15 min^40^), which is not compatible with long-lived activated caspases. Furthermore, the transient increase in extrusions following a pulse of optoDronc (returning to physiological levels after 50 min, Supplementary Fig. 3E, with extrusion lasting on average 20 min) is compatible with relatively short-lived (<30 min) active caspases. Alternatively, partial cleavage of caspase substrates facilitating the engagement in extrusion may explain this apoptotic priming. In this scenario, the timescale of memory should be related to the characteristic turnover of the targeted proteins. Accordingly, we tested different pathways known to be modulated downstream of caspases that could feed back on apoptosis susceptibility. For instance, JNK pathway activity can be tuned by caspase^60^ and directly impact apoptosis engagement, which can generate a positive feedback loop in dying cells^61^. However, while a fluorescent reporter of JNK (TRE-RFP^62^) shows activation in the midline, there was absolutely no sign of activation in the posterior domain, which is exposed to caspase activity for several hours during development of the notum (Supplementary Fig. 5A, Supplementary Movie 10). Alternatively, reactive oxygen species (ROS) can be triggered by caspases^33,43,63–66^ and could modulate apoptosis by increasing cellular toxicity. However, we could not detect clear activation of ROS in the posterior region using a transcriptional reporter (GstD-GFP, Supplementary Fig. 5B, Supplementary Movie 11), nor any induction of ROS following a sublethal pulse of optoDronc in one heminotum (Supplementary Fig. 5C). Proteasome inhibition was also shown to facilitate apoptosis engagement through the stabilisation of active caspases^9^, and several proteasome components were shown to be cleaved by caspases in S2 cells^67^. However, a live sensor of proteasome activity (proteoflux^68^) did not reveal distinctive change of protein degradation rate in the posterior domain (Supplementary Fig. 5D, Supplementary Movie 12). Sublethal caspase activity can also modulate cytoskeleton organisation^13^, hence modulating the probability to enter extrusion. Accordingly, sublethal Dronc activity was shown to downregulate MyoII in the pupal notum^69^, and global tension reduction was recently shown to be permissive for cell extrusion^70^. However, we could not detect clear modulation of MyoII intensity following sublethal pulses of optoDronc (Supplementary Fig. 5E, Supplementary Movie 13). We also did not notice any clear change of cell shape in the zone exposed to sublethal caspases, suggesting that cell mechanics is unlikely to be significantly altered. We also previously showed that disassembly of microtubules by caspases promotes cell deformability and the initiation of cell extrusion^42^. However, we could not detect any clear change in microtubule organisation upon sublethal optoDronc activation (Supplementary Fig. 5F, Supplementary Movie 14). Finally, we tested whether the pattern of Diap1 (the orthologue of XIAP1, a core inhibitor of apoptosis which can directly tune effector caspase activity^71–73^) could partially explain the spatial variability of optoDronc sensitivity. We designed a knock-in line of Diap1 tagged with fluorescent mScarlet to assess the variation of protein levels (since Diap1 can be regulated by protein degradation^72^). We observed a narrow band in the midline with low Diap1, as previously reported using a reporter of Diap1 degradation^27^, and a decrease in Diap1 levels at the posterior edge of the tissue at late stage of development (Supplementary Fig. 6A, Supplementary Movie 15). Yet, a more detailed spatial correlation quantification revealed a poor match between the pattern of cell death triggered by optoDronc in early and late pupae and the corresponding pattern of Diap1 (Supplementary Fig. 6C-F). Moreover, we saw no change in Diap1 levels following a sublethal pulse of optoDronc (Supplementary Fig. 6B, Supplementary Movie 16). Altogether, this suggests that the pattern of Diap1 protein level is unlikely to explain the priming. Accordingly, while Diap1 RNAi was sufficient to trigger ectopic caspase activation in clones (Supplementary Fig. 6G, Supplementary Movie 17), it did not change significantly the effector caspase activity threshold at which cells engage in apoptosis (Supplementary Fig. 6H) suggesting that Diap1 levels are unlikely to change effector caspase sensitivity (with the limit of the sensitivity of our sensor). However, in good agreement with previous results showing that Diap1 levels modulate sensitivity to pro-apoptotic signals^26,27^ and that Diap1 can modulate effector caspase activity downstream of Dronc^74^, we found that Diap1 overexpression in the notum drastically reduces the number of cell deaths triggered by a pulse of optoDronc activation (Supplementary Fig. 6I–K, Supplementary Movie 18). Altogether, these results confirm that Diap1 can modulate apoptosis entry downstream of Dronc, but is unlikely to modulate the effector caspase activity threshold at which cells engage in apoptosis and to explain the priming. Caspase activity was also shown to trigger DNA damage^25,75^ which could sensitise cells for apoptosis over long timescales, however, we could not detect any change of γH2A spots in the midline despite positive GC3Ai signals, while late apoptotic cells were, as expected, positively stained (Supplementary Fig. 5G). Thus, we could not identify a single obvious candidate explaining how past caspase activation sensitises cells for apoptosis, which may be compatible with a subtle and combinatorial contribution of the modulation of many caspase targets.

Interestingly, this work outlined the numerous cells which transiently activate effector caspases and yet survive. It remains unclear at this point whether such transient activations represent a failure to engage in apoptosis, a process akin to anastasis^29,76,77^, or whether they may also have a relevant physiological function in regulating cytoskeleton/shape or other cellular properties. What, then, could be the physiological relevance of the heterogeneity of caspase sensitivity? At the population level, the existence of heterogeneity and an almost linear correlation between probability to enter extrusion and caspase activity (Fig. 1E) suggests that caspase acts like a rheostat rather than a switch at the population level. The heterogeneity of caspase threshold would therefore be physiologically relevant as it would avoid massive simultaneous elimination of cells passing a threshold of caspase activity (hence leading to tissue rupture^40,78^) and instead progressively eliminate increasing proportions of cells.

Our work also suggests that the engagement in apoptosis is not totally cell-autonomous and relies on which cells among neighbours will engage in extrusion first. In this context, cells that were previously exposed to caspases and/or have relatively higher caspase activity are more likely to die first, either in a physiological context or upon mild caspase activation in clones. This non-autonomous decision is reminiscent of the process of cell competition whereby neighbouring cells trigger the elimination of suboptimal cells^79^. Interestingly, previous work in mouse embryonic stem cells already suggested that an increased susceptibility to apoptosis is sufficient to generate so-called “loser” cells that will be preferentially eliminated in a developmental context with a high apoptosis rate^80,81^. While *Drosophila* has been a central system to decipher the concepts and mechanisms of cell competition^82^, up to now there has been very little characterisation of spontaneous cell competition taking place during normal development. We propose that the relative modulation of caspase sensitivity by past exposure could constitute one example of such physiological cell competition which could bias clonal selection and the genetic make-up of adult tissues. Future work will help to characterise the contribution of such bias in pre-tumoural clone expansion and cancer progression.

## Methods

### Resource availability

#### Lead contact

Further information and requests for resources and reagents should be directed to and will be fulfilled by the lead contact, Romain Levayer (romain.levayer@pasteur.fr).

#### Material availability

All the reagents generated in this study will be shared upon request to the lead contact without any restrictions.

### Experimental model and subject details

#### Drosophila melanogaster husbandry

All the experiments were performed with *Drosophila melanogaster* fly lines with regular husbandry techniques. The fly food used contains agar-agar (7.6 g/l), saccharose (53 g/l), dry yeast (48 g/l), maize flour (38.4 g/l), propionic acid (3.8 ml/l), Nipagin 10% (23.9 ml/l), all mixed in one litre of distilled water. Flies were raised at 25 °C in plastic vials with a 12 h/12 h dark/light cycle at 60% moisture unless specified in the legends and in the table below (alternatively raised at 18 °C or 29 °C). Females and males were used without distinction for all the experiments. We did not determine the health/immune status of pupae, adults, embryos, and larvae; they were not involved in previous procedures, and they were all drug- and test-naïve.

#### Drosophila melanogaster strains

The strains used in this study and their origin are listed in Table 1.

**Table 1:** Drosophila *strains* used in the manuscript

Fly line	Chromosome location	Origin (citation)	Source
*E-cad::3XmKate (KI)*	II	^92^	BDSC_58789
*E-cad::GFP (KI)*	II	^92^	BDSC_60584
*sqh-Sqh:3XmKate2*	II	^93^	Yohanns Bellaïche
*UAS-his3.3 mIFP-T2A-H01*	III	^94^	BDSC_64184
*UAS-hid dsRNA*	III	VDRC	VDRC:v8269
*UAS-GC3Ai*	II	^46^	BDCS_84346
*UAS-GC3Ai*	III	^46^	BDCS_84343
*UAS-optoDronc::GFP*	II	^40^	^40^
*UAS-optoDronc*	II	This study	N/A
*UAS-Cd4-mIFP-T2A-HO1*	III	Bloomington	BDSC_64182
*hs-flp22; E-cad::3xmKate; act<cd2<gal4,UAS-Cd4-mIFP-T2A-HO1*	I; II; III	This study	This study
*GMR11F02-gal4 (pdm2-Gal4)*	III	Bloomington	BDCS_48928
*UAS-reaper; tub-Gal80*^*ts*^*/Cyo*	X; II	Eduardo Moreno	N/A
*hs-CL1::GFP*	II	^68^	^68^
*TRE-dsRed-40*	II	Dirk Bohman^62^	^62^
*GstD-GFP*	III	Bloomington	BDCS_26926
*Diap1::mScarlet-I (KI)*	III	This study	N/A
*UAS-αtub::mCherry*	II	^42^	^42^
*UAS-diap1 dsRNA*	III	Bloomington	BDCS_33597
*UAS-diap1*	III	Bloomington	BDSC_6657
*lexAOP-GC3Ai*	II	Our group^55^	Our group^55^
*Act-lexA*	II	Bloomington	BDCS_62567

The exact genotype used for each experiment is listed in Table 2.

**Table 2:** Genotype of every experiment associated by figure panel

**Figure**	**Genotype**	**Heat shock 37 °C**	**Stage**
1A-E	*w; E-cad::3xmKate (KI)/UAS-GC3Ai; pnr-Gal4/+*	N/A	16 h APF
1 F,G	*w; E-cad:: 3x mKate (KI)/UAS-optoDronc::GFP; pnr-Gal4/+*	N/A	20 h APF
S1A-H	*w; E-cad:: 3x mKate (KI)/UAS-GC3Ai; pnr-Gal4/+*	N/A	16 h APF
S1J	*w; E-cad:: 3x mKate (KI)/UAS-GC3Ai; pnr-Gal4/UAS-His3::mIFP-T2A-HO1*	N/A	16 h APF
S3A,B	*w; E-cad:: 3x mKate (KI)/UAS-optoDronc; pnr-Gal4/UAS-GC3Ai*	N/A	24 h APF
S3D,E	*w; E-cad:: 3x mKate (KI)/UAS-optoDronc::GFP; pnr-Gal4/+*	N/A	20 h APF
S3F,G	*w; E-cad:: 3x mKate (KI)/UAS-optoDronc; pnr-Gal4/UAS-GC3Ai*	N/A	20 h APF
2A-C	*w; E-cad:: 3x mKate (KI)/UAS-GC3Ai; pnr-Gal4/+*	N/A	16 h APF
2D,E	*w; E-cad:: 3x mKate (KI)/UAS-optoDronc::GFP; pnr-Gal4/+*	N/A	20 h APF
3 A	*w; E-cad:: 3x mKate (KI)/UAS-GC3Ai; pnr-Gal4/+*	N/A	18 h and 24 h APF
3 A,C	*w; E-cad:: 3x mKate (KI)/UAS-optoDronc::GFP; pnr-Gal4/+*	N/A	18 h and 24 h APF
3B	*w; E-cad:: 3x mKate (KI)/UAS-GC3Ai; pnr-Gal4/UAS-hid dsRNA*	N/A	18 h APF
3B,C	*w; E-cad:: 3x mKate (KI)/UAS-optoDronc::GFP; pnr-Gal4/UAS-hid dsRNA*	N/A	24 h APF
3D,E	*w; E-cad:: 3x mKate (KI)/UAS-optoDronc; pnr-Gal4/UAS-GC3Ai*	N/A	18 h APF
4B,C	*w; E-cad:: 3x mKate (KI)/UAS-GC3Ai; pnr-Gal4/+*	N/A	16 h APF
4D	*hs-flp22; E-cad:: 3x mKate (KI)/UAS-optoDronc::GFP; act<cd2<Gal4, UAS-Cd4::mIFP-T2A-HO1/+*	48 h ACI	24 h APF
S4A-F	*w; E-cad:: 3x mKate (KI)/UAS-GC3Ai; pnr-Gal4/+*	N/A	16 h APF
5B,C	*UAS-reaper/+; tub-Gal80*^*ts*^*/+; pdm2-Gal4/+*	N/A	96 h AEL
S5A	*w; E-cad::GFP (KI)/TRE-dsRed*	N/A	16 h APF
S5B	*w; E-cad::GFP (KI)/+; GstD-GFP/+*	N/A	16 h APF
S5C	*w; E-cad:: 3x mKate (KI)/UAS-optoDronc; pnr-Gal4, UAS-Cd4::mIFP-T2A-HO1/GstD-GFP*	N/A	20 h APF
S5D	*w; E-cad:: 3x mKate (KI)/hs-CL1::GFP*	N/A	16 h APF
S5E	*w; sqh:: 3x mKate /UAS-optoDronc; pnr-Gal4, UAS-Cd4::mIFP-T2A-HO1/+*	N/A	20 h APF
S5F	*w; UAS-αtub::mCherry/UAS-optoDronc; pnr-Gal4, UAS-Cd4::mIFP-T2A-HO1/+*	N/A	20 h APF
S5G	*w; UAS-GC3Ai/+; pnr-Gal4 /+*	N/A	20 h APF
S6A	*w; E-cad::GFP (KI)/+; diap1::mScarletI (KI)/+*	N/A	16h-31h APF
S6B	*w; UAS-optoDronc/+; pnr-Gal4, UAS-Cd4::mIFP-T2A-HO1/diap1::mScarletI (KI)*	N/A	20 h APF
S6C,D,F	*w; E-cad::GFP (KI)/+; diap1::mScarletI (KI)/+*	N/A	18 h, 24 h APF
S6E,F	*w; E-cad:: 3x mKate (KI)/UAS-optoDronc::GFP; pnr-Gal4/+*	N/A	18 h and 24 h APF
S6G,H	*hs-flp22; E-cad:: 3x mKate (KI)/act-lexA, lexAOP-GC3Ai; act<cd2<Gal4,UAS-Cd4::mIFP-T2A-HO1/UAS-diap1 dsRNA*	48 h ACI	24 h APF
S6I-K	*w; E-cad:: 3x mKate (KI)/UAS-optoDronc::GFP; pnr-Gal4/UAS-diap1*	N/A	18 h and 24 h APF

*ACI* time After Clone Induction, *APF* After Pupal Formation, *AEL* After Egg Laying.

### Generation of fly lines

#### Knock-In Diap1::mScarlet-I

CRSPR-mediated homologous recombination was achieved by injecting three plasmids, one corresponding to the donor template and two encoding the guide RNAs (gRNAs), into Cas9-expressing embryos (BL55821) by BestGene Inc and screened for 3xP3-DsRed insertion. The following gRNAs were selected using the Target Finder tool (https://flycrispr.org/target-finder/). The guide sequences (with the PAM sequence underlined) were:

gRNA1, 5′-GGCTCCGTCCGTAGCTCCCACGG −3′

gRNA2, 5′- GGTTGATGAGTAAGCTACATGGG −3 ′.

Corresponding oligonucleotides containing the guide were annealed and cloned into BsbI-digested pU6.3-gRNA

(DGRC Stock 1362; https://dgrc.bio.indiana.edu//stock/1362; RRID:DGRC_1362).

To produce the homologous repair construct pScarlessHD-Diap1-mScarlet-DsRed, all cloning was performed by using NEBuilder HiFi DNA Assembly Method. Linear DNA fragments were amplified by PCR and inserted into pScarlessHD-DsRed (DGRC #1364) see https://flycrispr.org/scarless-gene-editing/.

The final construct was checked by sequencing.

The homology arms (1 kb long) flanking the target sites were amplified from genomic DNA extracted from line BL55821, the mScarlet-I was amplified from pmScarlet-i_C1 (Addgene 85044). The candidate lines were sequenced to confirm successful editing. The -Dsred lines were obtained by crossing to line PBac-transposase (BL32070) and the removal of the DsRed cassette was confirmed by sequencing.

#### Design of UAS-optoDronc without GFP

The p3xUAS-DRONC-linker-CRY2PHR was generated by amplifying DRONC-linker-CRY2PHR from UAS-optoDronc::GFP plasmid, generated in ref. ^40^, by PCR and inserted in pJFRC4-3XUAS-IVS-mCD8::GFP (Addgene 26217) cut by NotI and XbaI (to excise mCD8::GFP) using NEBuilder HiFi DNA Assembly Method to generate p3xUAS-DRONC-linker-CRY2PHR. The construct was checked by sequencing and inserted at the attp site attp40A after injection by Bestgene.

The following primers were used for amplification:

#### DRONC-Linker-CRY2PHR F

TAACCCTAATTCTTATCCTTTACTTCAGGCGGCCGCAACATGCAGCCGCCGGAGCTCGA

#### DRONC-Linker-CRY2PHR R

CCACAGAAGTAAGGTTCC

### Dissection and immunostaining of pupae and larval wing disc

Dissection and immunostaining of nota were performed as indicated in ref. ^83^ with standard formaldehyde fixation and permeabilisation/washes in PBT 0.4% Triton. Briefly, the nota were pinched on small plates containing silicone, dissected in PBS, fixed in 4% Formaldehyde solution, washed in PBT 0.4% Triton, stained with primary antibody solution, washed and then stained with secondary antibody solution. The staining was performed on pupae expressing GC3Ai in the pnr-Gal4 domain. The following antibodies/markers were used: rat anti-E-cad (DCAD2 concentrate, DSHB, 1/50 dilution, monoclonal), rabbit anti-gamma H2AvD pS137 (1/500 dilution, Rockland 600-401-914). Secondary antibodies were ultrapurified Donkey anti-rabbit Cy3 (1/200, Jackson ImmunoResearch/711-165-152, lot 168268) and ultrapurified Donkey anti rat alexa 647 (1/200, Jackson ImmunoResearch/712-605-153, lot 135094). Dissected nota were mounted in Vectashield with DAPI (Vectorlab). Dissections of larval wing imaginal discs were performed in PBS on ice. Dissected discs were fixed for 20 min in 4 % formaldehyde (SIGMA F8775), rinsed 3 times in PBT (PBS 0.4% Triton), followed by 10 min permeabilisation in PBT. Primary and secondary antibodies were incubated for 2 h at room temperature (or 12 h at 4 °C) under rocking agitation. After each antibody incubation, discs were rinsed 3 times in PBT, followed by 3 washes of 30 min. Discs were mounted in Vectashield® (EUROBIO SCIENTIFIC/H-1000) and imaged with an LSM880 equipped with a fast Airyscan using a 40X oil objective. The following primary antibodies were used: rat anti E-cadherin (1/100, DCAD2 concentrated DSHB, monoclonal), mouse anti DSRF (1/500, gift of Seth Blair), rabbit anti RFP (1/500, abcam 62341, lot GR3221308-3), rabbit anti cleaved DCP-1 (1/100, Cell Signalling 9578S, lot 5). Secondary antibodies were: ultrapurified Donkey anti rabbit Cy3 (1/200, Jackson ImmunoResearch/711-165-152, lot 168268), ultrapurified anti mouse 488 (1/500, Jackson ImmunoResearch/715-476-151, lot 158851), ultrapurified anti rat alexa 647 (1/500, Jackson ImmunoResearch/712-605-153, lot 135094).

### Live imaging and movie preparation

Notum live imaging was performed as followed: the pupae were collected at the fluid stage (0–10 h after pupal formation), aged at 22 °C or 29 °C, glued on double-sided tape on a slide and surrounded by two home-made steel spacers (thickness: 0.64 mm, width: 20 x 20mm). The pupal case was opened up to the abdomen using forceps and mounted with a 20x40mm #1.5 coverslip buttered with halocarbon oil 10S. The coverslip was then attached to spacers and the slide with two pieces of tape. Pupae were collected 48 after clone induction and dissected usually at 16 to 18 h APF (after pupal formation). The time of imaging for each experiment is provided in the table above. Pupae were dissected and imaged on a confocal spinning-disc microscope (Gataca Systems) with a 40X oil objective (Nikon Plan Fluor, N.A. 1.30), a Zeiss LSM880 (Zen Black software) equipped with a fast Airyscan using an oil 40X objective (N.A. 1.3), or a Zeiss LSM980 (Zen Blue software) equipped with a fast Airyscan 2 using an oil 40X objective (N.A. 1.3). Z-stacks (0.5 or 1 µm/slice) were captured every 1, 2, 5 or 10 min using autofocus at 22 °C. The autofocus was performed using the plane of E-cad or another apical membrane marker as a reference, using a Zen Macro developed by the Jan Ellenberg laboratory (MyPic) on the LSM880, the built-in autofocus function of Zen Blue on the LSM980, or a custom-made Metamorph journal on the spinning disc. Movies were performed in the nota close to the scutellum region containing the midline and the aDC and pDC macrochaetae. Movies shown are adaptive local Z-projections. The plane of E-cad or other apical membrane marker was used as a reference to locate the surface of interest on spatial windows of the image (using the maximum in Z of average intensity or the maximum of the standard deviation). Other channels were then projected with a maximum intensity z-projection using an offset and projection stack size appropriate for the localisation of the signal of interest. This procedure was either applied using a custom Matlab routine^39,84^. On some occasions where this local Z-projection pipeline could not accurately resolve folds in the tissue or would project the signal of the cuticle above the apical cell surface of interest, the Zellige^85^ Fiji plugin was used to locate and project the surface channel, and the resulting z-height maps were saved and imported into the custom local Z-projector Matlab routine as a reference surface from which other channels could be projected if necessary. Automation of the Zellige plugin to run on multiple timepoints was achieved with a custom ImageJ macro.

### Optogenetic control of caspase with optoDronc

Crosses involving expression of optoDronc were maintained in the dark at 25 °C. Collection of pupae and dissection were performed on a binocular with LEDs covered by a home-made red filter (Lee colour filter set, primary red) after checking that blue light was effectively cut using a spectrometer.

#### Graded activation of optoDronc

For experiments involving graded or ‘ramp’ activation of optoDronc, blue light pulses were administered by acquiring single z-stacks (10 planes of variable interval to cover the apical surface of the notum) in regions of the tissue at variable exposure times. optoDronc pulse images were acquired on a Zeiss LSM880 in fast AiryScan imaging mode using an oil 40X objective with 488 nm laser power at 2.8% and pixel dwell of 0, 2.048, 4.096, or 8.192 μsec/pixel depending on the position in the ramp.

#### Mild induction of optoDronc

For experiments involving mild induction of optoDronc, either globally, in regions of the tissue or in clones, blue light pulses were administered by acquiring images using the 488 nm laser either as a short time series (5 or 10 single planes within 5 min) or a single z-stack (10 planes of variable interval to cover the apical surface of the notum). optoDronc pulse images were acquired on a Zeiss LSM880 in fast airyscan imaging mode using an oil 40X objective with 488 nm laser power 2.8% and pixel dwell of 1.08µsec (for global activation of optoDronc) or 1.02µsec (for local and clonal activation).

#### Full activation of optoDronc

To assess the response of the effector caspase sensor GC3Ai to full and prolonged activation of caspase with optoDronc, z-stacks of 22 slices (1 µm/slice) were captured on a Zeiss LSM880 in fast airyscan mode every 2 mins using the 488 nm laser (2.8% power, pixel dwell 0.54µsec) to excite optoDronc and image GC3Ai intensity, and the 561 nm laser to image E-cad::mKate at the cell apical junctions.

### Image analysis

#### Cell segmentation

In order to extract quantitative data from individual cells over time, cells were segmented using the EPySeg Python package on the Z-projected E-cad surface. The model used for segmentation was either the built-in v2 model from EPySeg^44^, or a version of this model after re-training on our own previously corrected segmentation masks. Correction of segmentation masks was performed either using the Fiji plugin TissueAnalyzer or the Napari plugin EpiCure^45^. Corrected segmentation masks were then exported for cell tracking.

#### Tracking using skimage and trackpy

For each of the large segmented GC3Ai movies, the input is an array of segmented images $({{{{\rm{I}}}}}_{{{{\rm{k}}}}})_{{{{\rm{k}}}}=1,...,{{{\rm{N}}}}}$, where ${{{\rm{k}}}}$ is a time frame, and each image ${I}_{k}$ is a snapshot of a tissue at time ${{{\rm{t}}}}={{{\rm{k}}}}\times 5\min$. Each image is a binary mask, where the value 1 (or 255) represents the boundary between two cells, and 0 is either intracellular space or background. The ‘skimage’ library of Python was used to detect and label cells as connected regions in each image. Furthermore, the x,y position of each connected region in the image was saved, as well as its geometric properties such as area, perimeter, eccentricity, solidity, orientation, and the coordinates of each pixel belonging to that connected region. All this was saved in one dataframe structure of the’pandas’ Python library.

The ‘trackpy’ library of Python was then used to track the connected regions over time. A pixel range of 15 was used with the adaptive step 0.95. Additionally, it was permitted for a cell to ‘disappear’ from the tracking during one time frame. This was done in order to compensate for some errors that occurred in the segmentation, such as when a real boundary between two cells was not detected in one image. The result was a dataframe as before, with a new label named ‘particle’, that maintains the same value for different time frames for the same connected region.

We eventually removed border cells, sensory organ precursor cells (SOP), and too short-lived cells from the analysis. Border cells were identified by pixels touching the edges of the image and were flagged as background. Then, using the ‘skimage’ library, the background regions were dilated with 3 × 3 kernels, and cells that overlapped with this dilated background were then flagged as border cells. Sensory organ precursor (SOP) cells are specialized mechanosensory cells that behave differently from their epidermal neighbours in the notum. Since SOPs grow to a larger size than their epidermal neighbours at late developmental stage, any cell that in any moment reached an area of 3000 pixels (89.79 μm^2^) was removed from analysis. Any particle that lived less than 15 time frames (75 min) was also removed from the analysis.

Following this curation, the remaining tracked particles were those that lived long enough, were not too large (not SOPs) and were never at the edge of the tissue. These relevant cells were used for all further analysis.

#### Detecting dying cells using morphological features

The identification of dying cells was based on the identification of cells that do not appear in the final time frame and that undergo apical constriction. The raw signal of apical area was smoothed to remove noise. As some tracked cells disappeared over one time frame, the ‘numpy’ library was used to interpolate the signal over those time frames that lack information about the cell. Then, a median filter of kernel size 15, from ‘scipy.signal’ library in Python, was used to smooth the signal. Cells whose smoothed area shrank at least 50% over the last 75 min of their life were tagged as dying cells. Their raw area was then assessed to ensure that the area was in progressive decline in the last 4 time points, i.e. 20 min prior to disappearance.

#### Detection of area inflection point as the onset of cell extrusion

The onset of extrusion was defined as the moment when a dying cell starts reducing in size before its full delamination and disappearance from the tissue. A ‘two-line fit’ procedure was used to detect this moment automatically for each dying cell.

A given dying cell lives on a time interval $[{{{{\rm{t}}}}}_{0},{{{{\rm{t}}}}}_{{{{\rm{end}}}}}]$, and its area is recorded on every 5-min time step in this interval. At times $\{{{{{\rm{t}}}}}_{0},{{{{\rm{t}}}}}_{1},{{{{\rm{t}}}}}_{2},...,{{{{\rm{t}}}}}_{{{{\rm{end}}}}}\}$, it has a recorded area, $\{{{{{\rm{a}}}}}_{0},{{{{\rm{a}}}}}_{1},{{{{\rm{a}}}}}_{2},...,{{{{\rm{a}}}}}_{{{{\rm{end}}}}}\}$. As the cell does not exist after ${t}_{{end}}$, one more time step is defined: ${t}_{{end}+1}={t}_{{end}}+5\min$, and the final size of the cell ${a}_{{end}+1}=0$. The time observations were then split in two sets $\{{{{{\rm{t}}}}}_{0},...,{{{{\rm{t}}}}}_{{{{\rm{k}}}}}\}$ and $\{{{{{\rm{t}}}}}_{{{{\rm{k}}}}+1},...,{{{{\rm{t}}}}}_{{{{\rm{end}}}}+1}\}$, and using the Python library ‘statsmodels.api’, two separate ordinary least squares models were fit to the time-area points. From both models, the sum of all residuals was extracted, and then the residuals from the left-line and right-line fit were summed. This value was saved as ${r}_{k}$.

This process was repeated for every ${{{\rm{k}}}}=1,...,{{{{\rm{t}}}}}_{{{{\rm{end}}}}}$, producing a set of residuals $\{{{{{\rm{r}}}}}_{1},...,{{{{\rm{r}}}}}_{{{{\rm{end}}}}}\}$. At the minimum value in this set, ${r}_{m}$, the corresponding ${t}_{m}$ was defined as the inflection point of the cell area and the onset of extrusion for the given cell, ${t}_{{onset}}={t}_{m}$.

Some dying cells divide in the course of their life, and this method could wrongly attribute the moment of division as the onset of extrusion. For this reason, the inflection point analysis was restricted to the last hour (last 12 recorded points) of a dying cell life, so the time interval of interest was in fact $[{{{{\rm{t}}}}}_{{{{\rm{end}}}}}-60\min,{{{{\rm{t}}}}}_{{{{\rm{end}}}}}]$.

#### Interpolation and smoothing of signals

It is worth noting that raw recordings of signals (area, perimeter, GFP levels) are noisy. Even though the quality of imaging is high, the raw signals will always have some amount of technical error. Parameters such as area, perimeter, eccentricity were smoothed using a median filter with kernel size 15, which was chosen preferentially over a mean filter due to the fact that it shortens the signal for the size of its kernel.

The GC3Ai signal (mean GFP intensity) was smoothed using a Savitzky-Golay filter, which fits successive subsets of adjacent data points with a low-degree polynomial using linear least squares. This was implemented using the ‘scipy.signal’ library of Python with kernel size 15 and the 3rd-degree polynomial. Furthermore, such smoothing enables for an easy calculation of the derivative of the signal, in this case, the caspase levels. The derivative of the signal would be the derivative of the polynomial, and in practice, we only need to specify the parameters of the function.

While GC3Ai signal should cumulate in the cell, signal can also fluctuate due to adaptive z-projection which can generate some negative values in the GC3Ai derivative remaining close to 0. We decided to set all these values to 0.

After running the full image analysis pipeline on both movies separately and confirming that the same relationships and conclusions were drawn from each, the two datasets were pooled for greater statistical power and more information for machine learning after rescaling raw GC3Ai signal distribution using the median.

### Caspase threshold search and logistic regression

To investigate the existence of an overall threshold that determines if a cell in the tissue will extrude, all values of smoothed GC3Ai intensity and caspase activity from all cells, dying and non-dying, at all time points, were taken into account. This analysis was performed on more than 15,000 cells, where each cell lives at least 15 time frames and at most 192 time frames.

For a cell, c, we have values of a signal $x=\{{x}_{k}\}_{k={t}_{0},..,{t}_{{end}}}$, where ${x}_{k}$ is a value of a signal (GC3Ai or caspase) at time frame ${{{\rm{k}}}},{t}_{0}$ is a time frame when the cell c is first observed, and ${t}_{{end}}$ is its last time frame.

Each ${x}_{k}$ of every cell is assigned a value, labelled with

$${y}_{k}=\left\{\begin{array}{c}0,{if\,cell\;is\;extruding},\\ 1,{otherwise}.\end{array}\right.$$

If a cell does not die during the movie, all values $\{{y}_{k}\}_{k={t}_{0},..,{t}_{{end}}}$ are equal to 0.

If a cell dies during the movie, we identify its extrusion time ${t}_{{onset}}$, then

$${y}_{k}=\left\{\begin{array}{c}0,k < {t}_{{onset}},\\ 1,k\ge {t}_{{onset}}.\end{array}\right.$$

A logistic regression was performed on $({{{{\rm{x}}}}}_{{{{\rm{k}}}}},{{{{\rm{y}}}}}_{{{{\rm{k}}}}})$ pairs of values, fitting a logistic model (sigmoid function) to the data. This function takes real values and outputs a value between 0 and 1. We interpret it as probability to have a certain value of ${x}_{k}$ to be labelled with 0 or 1. A good fit of the model and a sharp transition between the two states (not extruding and extruding) would indicate the existence of a threshold of the signal (either GC3Ai or caspase activity) that determines if the cell is extruding or not.

The goodness of fit of each logistic model to the real data was assessed by calculating the Tjur pseudo R-squared (Tjur’s R^2^, also known as the coefficient of discrimination). Tjur’s R^2^ compares the average fitted probability of the two outcomes, and is calculated as followsdrosophil

1 $${R}_{{Tjur}}^{2}=|\hat{\pi }\left(y=1\right)-\hat{\pi }\left(y=0\right)|,$$

where $\hat{\pi }\left(y=1\right)$ is the average predicted outcome from the model when y = 1, and $\hat{\pi }\left(y=0\right)$ is the average predicted outcome when y = 0. The value of Tjur’s R^2^ can be interpreted as follows (see Supplementary Fig. 1 for more details):

${R}_{{Tjur}}^{2}\approx 1$: The model has absolute predictive power, values of the y variable are segregated perfectly by the x variable.

${R}_{{Tjur}}^{2}\approx 0$: The model has no predictive power, values of the y variable are not segregated by the x variable.

$0 < {R}_{{Tjur}}^{2} < 1$: The model has some predictive power, values of the y variable are somewhat segregated by the x variable.

### Testing correlation between caspase activity at the onset of extrusion and GAL4 expression

To assess whether cell-to-cell variability in measured caspase activity at the point of commitment to death could actually be explained by differences in expression of the caspase sensor under the control of the GAL4-UAS system, both the UAS-GC3Ai caspase sensor and the constitutively expressed UAS-His3-IFP far-red histone marker were expressed in the same notum under the control of the notum-wide *pnr-G4* driver. Extrusion events were automatically detected using DeXtrusion^86^ and segmented using the TissueAnalyzer Fiji plugin^87^, and ROIs of segmented extruding cells were used to measure the evolution of perimeter and GC3Ai intensity over time. Cell perimeter was smoothed using a mean filter of kernel size 5 datapoints, and a custom MATLAB routine was used to automatically detect the perimeter inflection point using a two-line fit and assign this as the onset of extrusion (using the same logic as the approach detailed in methods section “Detection of area inflection point as the onset of cell extrusion”). Raw GC3Ai intensity of extruding cells was smoothed over 3 datapoints, the derivative of this signal was calculated to quantify the rate of change, and this derivative was smoothed over 3 datapoints (hereafter “caspase activity”). The cytoplasmic GC3Ai signal was used to follow the cell shape of extruding cells in z from their apical to nuclear planes and identify their respective His3-IFP-marked nuclei, maximum intensity local z-projections of which were segmented using the StarDist Fiji plugin^88^. Caspase at the onset of extrusion was plotted against His3-IFP mean intensity at the onset of extrusion, and the correlation was calculated using Spearman’s R.

### Measuring caspase threshold in Diap1 RNAi clones

Dying cells were manually detected in WT and UAS-diap1 RNAi clones and segmented using EPySeg and EpiCure. Caspase activity was detected using the act-lexA driven lexAOP-GC3Ai. The same procedure as for large WT movies was applied for extrusion onset detection and caspase activity estimation.

### Correlation between cell parameters and caspase activity at the onset of extrusion

To assess the strength of the correlation between caspase activity at the onset of extrusion and other features of dying cells over time, the area inflection point of each dying cell was detected as previously described, and the cell’s value of caspase activity (smoothed derivative of mean GC3Ai intensity) at that time point was taken as its caspase activity at the onset of extrusion. All dying cells were aligned in time by their onset of extrusion (t_onset_), and their values of various morphological and caspase features in windows of time prior to the onset of extrusion were extracted. Specifically, for each dying cell, values of caspase activity, GC3Ai intensity, area, perimeter, derivative of area, derivative of perimeter and eccentricity were averaged in a 20-minute sliding window moved in 5-minute increments over the period covering −10hrs to 0hrs relative to the onset of extrusion. Using Python, linear correlations were then calculated between the values of each parameter in each time window and caspase activity at the onset of extrusion, such that the correlation coefficient R could be computed for each relationship and used to populate a matrix showing the strength of the association between the level of active caspase at the onset of extrusion and the value of each parameter across time prior to extrusion.

### Measuring response to graded optoDronc activation

To assess the effect of increasing optoDronc activation on resulting effector caspase activation and death, a non-GFP-tagged version of optoDronc was combined with the live effector caspase activity sensor GC3Ai in pupae in which cell apical junctions were labelled with E-cad::mKate. A ramp of graded optoDronc activation was performed by locally exposing regions of the lateral domains of a single notum to 488 laser excitation for increasing amounts of time. Cell deaths within the exposed regions were tracked by imaging E-cad::mKate for 1 h following optoDronc activation, and manually placing a point ROI at the coordinates of closure of an extruding cell in Fiji. 1 hour after optoDronc activation, a single z-stack was captured in both red and green across the whole notum to image both E-cad::mKate and GC3Ai. The proportion of total extrusions was calculated for each region of each notum by dividing the number of extrusions within that region by the total number of extrusions from all regions in the notum, and multiplying by 100. Normalised mean GC3Ai intensity was calculated for each region of each notum by measuring the mean intensity of GC3Ai within that region, then dividing the value by that measured for the unexposed region (exposure time 0 μsec/pixel).

### Mapping deaths following optoDronc activation

Coordinates of cell death events (x,y,t) following global mild optoDronc activation were registered manually by placing a point ROI at the coordinates of closure of an extruding cell in Fiji. The coordinates of the 4 anterior and posterior dorsocentral macrochaetae (aDC and pDC) in each notum were also manually registered to be used as landmarks for Procrustes transformation. The same process was carried out for control nota which expressed optoDronc but received no blue light pulses. Procrustes transformation was carried out using the built-in procrustes function in MATLAB to take the landmark positions from each mapped notum, translate them to the same origin, scale them to the same size and rotate them until the coordinates of corresponding landmark positions aligned as closely as possible. This allowed the Procrustes-transformed coordinates of extrusions from each notum to be superimposed on a common spatial map of the notum. The number of extrusions in discrete spatial windows was calculated by sorting points into bins by their Procrustes-transformed coordinates on a 15 × 15 density heatmap (covering roughly 5 x 5cells). A single density heatmap was generated by summing all values for each group of nota. In the case of the density histograms shown in Fig. 1G, the control map was then subtracted from the blue light pulses map through simple matrix subtraction to generate a map showing the spatial distribution of additional optoDronc-induced deaths.

To compare the spatial distribution of optoDronc-induced deaths at different developmental stages with the pattern of GC3Ai at the relevant stage, single images of GC3Ai distribution from the same notum at early and late stage of development were used to generate maps by averaging the fluorescent intensity of the sensor in spatial windows on a 15×15 grid (each box in the grid has width, height = 23.45 µm). Maps of optoDronc-induced deaths from early and late pupae were generated as described above, this time using Procrustes transformation to align the coordinates of extrusions to the notum from which the GC3Ai map was generated as a reference. Furthermore, the limits of the extrusion density map were this time restricted to the limits of the GC3Ai reference image, to ensure a more like-for-like comparison of spatial windows between the two maps. Simple pairwise correlation between bins on the heatmaps of GC3Ai and extrusions at the relevant developmental stage was then calculated using MATLAB to assess the strength of the relationship between the two classes of spatial map.

The extent to which optoDronc-induced deaths were clustered in the case of early, late, and *hid* RNAi nota was assessed by measuring spatial autocorrelation of the summed maps of extrusion events from all nota in each group. Spatial autocorrelation was measured by calculating global Moran’s *I* using the moran.test function as implemented in the ‘spdep’ R package^89^. Global Moran’s *I* reports the extent to which the value of each position in the grid is related to the value of the neighbouring positions (here considering ‘rook’ neighbours, i.e. adjacent neighbours but not diagonal ones), and therefore provides an index of clustering/dispersion. The value can be interpreted as follows:

0<I<1: data are more spatially clustered than expected from a random distribution

I≈1: data are randomly spatially distributed

−1 < I < 0: data are more spatially dispersed than expected from a random distribution

### Calculating death probability following optoDronc pulse as a function of GC3Ai at t0

To assess the probability of cells in regions of high endogenous caspase to undergo cell death following mild optoDronc activation as a function of their cumulative caspase activity at t0, the GC3Ai sensor was combined with a non-GFP-tagged version of optoDronc. A single z-stack was captured at t0 to image E-cad::mKate and GC3Ai, and simultaneously trigger mild optoDronc activation (see methods section ‘mild induction of optoDronc’ for precise conditions of optogenetic activation). Cell contour was then imaged and projected over the following 90 min, and extrusions were manually tracked by placing a point ROI in the centre of each dying cell at the start of the movie. The image of E-cad::mKate and GC3Ai at t0 was segmented using EPySeg and corrected using the Fiji plugin TissueAnalyzer. The segmentation mask was then exported as a set of Fiji ROIs, which were used to measure the mean intensity of GC3Ai within each cell. Extrusions were assigned by taking the coordinates of each manually tracked extrusion and measuring the Euclidean distance between this point and the centroid of each cell in the mask. The cell with the minimum distance between its centroid and this point was considered to be the dying cell. Normalised GC3Ai values were calculated by dividing all GC3Ai intensity measurements by the maximum GC3Ai value from their respective movie, to allow data from multiple movies to be pooled. Values of normalised GC3Ai intensity were binned and the proportion of extruding over total number of cells of this bin was calculated to show the relationship between cumulative caspase activity at the onset of optoDronc activation and probability of death in the following 90 min.

### Triggering past caspase activity in clones and calculating rate of death

To assess the difference in rate of death between WT clones and clones with past caspase activity within the same tissue, optoDronc was expressed in clones. Virgin female flies with the genotype *hsflp; E-cad::mKate/CyO; act>cd2* > *GAL4,UAS-cd4-mIFP* were crossed with males of the genotype *UAS-optoDroncGFP/CyO; MKRS/TM6B*. Clones were induced through a 15 min heat shock in a 37 °C water bath, and tubes were then maintained in the dark at 25 °C. Fluid-stage pupae were collected around 32 hours after clone induction and placed at 22 °C, then imaged at 48 hours after clone induction. Mild optoDronc activation in clones was induced by capturing a single z-stack exposing the notum to blue light (see methods section ‘mild induction of optoDronc’ for precise conditions of activation), then the cell contour and clones were imaged for the following 5 h using the E-cad::mKate and mcd4-IFP markers, respectively. All extrusions in the period of 1.5 hrs–5 hrs following optoDronc activation were tracked manually by detecting the closure of delaminating cells, and each event was scored as either a WT or clonal death. We focused on the posterior region where caspase gets developmentaly activated later (see movie S1). The image of cell contour at 1.5 hrs following optoDronc pulses was segmented using the Python package EPySeg, and corrected using the napari plugin EpiCure. Cells at the border of the image were removed from the segmented cell mask; the number of clone cells was detected manually by counting cells in the mask expressing the mcd4-IFP marker, and the number of WT cells was calculated by subtracting the number of clone cells from the total number of cells in the mask. Data from two movies were pooled, and the death rate was calculated for both WT and clonal populations by dividing the number of deaths in each population by the cell number at 1.5 h, then multiplying by 100 to be expressed as a percentage.

### Assessing rate of protein turnover with Proteoflux

To investigate any difference in the rate of protein turnover in distinct regions of the pupal notum, a pulse-chase experiment was carried out with the Proteoflux tool. Fluid-stage pupae expressing the Proteoflux tool under the control of a heat-shock promoter were selected, aged overnight at 22 °C, then mounted on double-sided tape on a slide and expression of Proteoflux was induced by placing the slide in an incubator at 37 °C for either 60 or 90 min. The pupae were then removed from the incubator, immediately dissected and imaged on a confocal spinning disc microscope (Gataca systems) with a 40X oil objective (Nikon plan fluor, N.A. 1.30) to capture the decay of the fluorescent signal.

### Machine learning analysis

#### Defining cell groups

To define cell groups for death prediction analysis, all cells touching a dying cell at its onset of extrusion (t_onset_) were designated as neighbours, and the collection of neighbour and dying cell was designated as a group. With 1130 dying cells in the segmented dataset, as many groups were defined. The groups contain between 5 and 11 neighbours.

To define groups of neighbours, for a dying cell ${c}^{D}$:

1. Find the time of the onset of extrusion, ${t}_{{onset}}^{D}$
2. From the time frame at ${t}_{{onset}}^{D}$
  I. Get coordinates of all pixels of the dying cell ${c}^{D}$, and create a mask ${M}^{D}$, such that ${M}^{D}$ is a matrix of the full image size, where each element of a matrix corresponds to a given pixel in the image and ${M}_{{ij}}^{D}=1$ if a pixel $({{{\rm{i}}}},{{{\rm{j}}}})$ belongs to ${c}^{D}$, and 0 otherwise.
  II. Dilate the mask ${M}^{D}$ and find all cells in the same time frame that have some intersection with ${M}^{D}$.
  III. Verify if the intersecting cells are in the set of relevant cells, if yes - add them to the list of neighbours of ${c}^{D}$.

#### Predicting dying cells using instant GC3Ai and caspase levels

Among groups containing a dying cell and all of its neighbours, we compared absolute GC3Ai intensity and caspase activity at the onset of the first extrusion and counted the proportion of groups where the first dying cell has the highest relative GC3Ai or caspase activity.

#### Time series forest (TSF)

The question of predicting which cell in the group of cells will be the first to die can be formulated as a time series classification problem, i.e., a machine learning problem. Due to the temporal structure of the input data, standard machine learning algorithms that are designed for structured data are usually not well suited. In particular, the order of the values is an essential part of time series, and consecutive time points are likely to be highly correlated.

Time series forest^48^ is a relatively intuitive algorithm. It derives summary features for all time series by dividing them into intervals and summarising each interval by its mean, standard deviation and gradient. Then a Random Forest-like strategy is employed to select from a random subset of these features at each node in each of an ensemble of trees. A novel selection criterion is used that considers both entropy gain and the margin by which a feature separates the classes. This continues until the entropy gain ceases to improve, at which stage the node is defined as a leaf. TSF has been shown to be an accurate classifier while being computationally efficient. Of note, the random forest algorithm (including TSF) has an out-of-bag (OOB) error estimate, which can be used instead of cross-validation. By construction, random forest uses a bootstrapping method - creates subsets of the training dataset and trains one tree on each subset. The final prediction is created by aggregating all predictions. For every subset, there are some missing data in the training set that are then used for model validation, also called out-of-bag validation. The out-of-bag score is evaluated by aggregating predictions for all samples from the trees that did not use them in the training.

#### Training and testing dataset

We want to investigate if the past behaviour of some cell variable could be informative in predicting which cell in a given group of cells is going to die first.

The groups of neighbours are defined as above - one dying cell and its neighbours at the time of onset. Now we look at the past behaviour of these cells. We have opted to use a sliding-window strategy to investigate if and which signal is predictive of a dying cell. We define a time interval ${{{{\rm{I}}}}}_{{{{\rm{w}}}},{{{\rm{z}}}}}$, with window size, ${{{\rm{w}}}}$ and ending position with respect to the onset of extrusion of a dying cell of a group, ${t}_{{onset}}-z$, so that

$${I}_{w,z}=[{t}_{{onset}}-z-w,{t}_{{onset}}-z].$$

For example, for a time window of 1 h that start 90 min prior to the onset of extrusion and ends 30 min prior to the onset, the interval we will look at is ${I}_{w,z}=[{t}_{{onset}}-90\min,{t}_{{onset}}-30\min ]$. Now, if the dying cell of a group entered extrusion at, for example ${t}_{{onset}}=100\min$, the time interval of interest for us will be ${I}_{w,z}=[10\min,70\min ]$, i.e. in time frames [2,14]. In absolute terms, each group of neighbours is analyzed based its own time of onset, so the actual time interval is group-specific, so the analysis is given in terms of ‘how far are we from the onset’.

For training TSF models, we use only the information from one signal on the given interval. The algorithm to train one TSF model for given parameters ${{{\rm{w}}}}$ and ${{{\rm{z}}}}$ is as follows:

for each group of neighbours

find the onset of extrusion of the dying cell, ${t}_{{onset}}$

define $${I}_{w,z}$$

for each cell ${{{\rm{c}}}}$ in the group

${X}_{c}=$ the signal from cell c on ${I}_{w,z}.$

if a cell is a dying cell of the group

$${Y}_{c}=1$$

else

$${Y}_{c}=0$$

The full dataset is a list of all time series, $X=\{{X}_{c}\}$, for all cells from all groups of neighbours, with corresponding classifications $Y=\{{Y}_{c}\}$. If no other transformation is made, the neighbouring cells are related to each other through the definition of the time interval used for the extraction of the signal. Note that for every new set of parameters ${{{\rm{w}}}}$ and ${{{\rm{z}}}}$ we train different TSF model.

We use 70% of randomly picked data for training and the remaining 30% of data for testing. This means that if a given dying cell is selected to be used for training, all of its neighbours will be used for training.

#### Hyperparameters

We use the implemented version of the TSF algorithm in Python from **the pyts** library [4]. There are a few hyperparameters that need to be set for good model fitting. Most notably, we need to set n_estimators, the number of trees in the forest. Following an example given in^90^, we use the *out-of-bag* error in order to estimate a suitable value for n_estimators at which the error stabilises. Finally, we use n_estimators = 125.

#### Training

We first trained TSF models using all neighbours in the training datasets. For each TSF_w,z_ model, which is defined by the window size ${{{\rm{w}}}}$ and the final time point prior to the onset of extrusion ${{{\rm{z}}}}$, we train models based on the:

- GC3Ai signal, caspase activity, area, perimeter and eccentricity.

We vary parameters w and z as follows:

$$-w\in [1{{{\rm{hr}}}},2{{{\rm{hr}}}},3{{{\rm{hr}}}},4{{{\rm{hr}}}}],$$

$$-z\in [10\min,20\min,30\min,1{{{\rm{hr}}}},2{{{\rm{hr}}}},3{{{\rm{hr}}}},4{{{\rm{hr}}}}].$$

Note that for larger values of parameters ${{{\rm{w}}}}$ and ${{{\rm{z}}}}$ we have fewer cells in the datasets.

#### Balanced datasets

In a movie 1 we detected 514 dying cells. In a movie 2 we detected 616 dying cells. Each of these dying cells has between 5 and 11 neighbours at the onset of extrusion. For an easier interpretation we can use balanced datasets - for one dying, take only one non-dying neighbour. For balanced datasets we add one layer of randomisation of our dataset as we choose one random neighbour from each group of neighbours.

#### Scores

For testing how well the trained model performs on a new, never-seen, dataset we use previously selected test dataset. Then we ask a model to predict if the given cell in a test dataset is dying or living, i.e. the model is classifying the given time series into two classes, 0 or 1. The success can be measured with different scores. Firstly, we can count how many times the model predicted a positive outcome (class 1, i.e., a dying cell) correctly - true positives TP, or wrongly - false positives FP. Then we count how many times the model predicted a negative outcome (class 0, i.e., non-dying cell) correctly - true negatives TN, or wrongly - false negatives FN. Then the scores are defined as follows:

1. Accuracy = $\frac{{TP}+{TN}}{N}$, tells us how many true predictions have been made overall.
2. Precision = $\frac{{TP}}{{TP}+{FP}}$, tells us how many true predictions have been made among all positive predictions.
3. Recall = $\frac{{TP}}{{TP}+{FN}}$, tells us how many true predictions have been made among all cases where the prediction should have been positive.
4. F1 score = 2 × precision × recall/(precision + recall), tells us how effectively a model makes a trade-off between precision and recall. Notice that F1 score goes to zero if any of the components goes to zero.

#### TSF prediction in a group of neighbouring cells using relative values

Another way to test and score a given TSF model is to ask it to predict the dying cell among a given group of cells. Rather than the overall classification of the time series, we are in fact interested to know which of the given cells (typically neighbouring cells) is the most likely to die first. Our metric will take a group of cells and instead of asking a model which class each cell belongs to, we ask the model which cell has the highest probability to be a dying cell.

**for** each group of neighbours in **test dataset**

for each cell in the group

${P}_{{cell}}$ = probability that the cell is a dying cell

${c}_{{best}}$ = the cell with the highest ${P}_{{cell}}$ in the group

**if** ${c}_{{best}}$ is the dying cell of the neighbourhood

good_predictions += 1

The maximum value for good_predictions is the total number of groups we have in the test dataset. The score is then defined as

local accuracy = good_predictions/total number of groups in the dataset.

For this task, we used relative GC3Ai and caspase activity values between neighbours for training the TSF model. The modified datasets are created as follows,

for each group of neighbours

find the onset of extrusion of the dying cell, ${t}_{{onset}}$

define $${I}_{w,z}$$

${{{\rm{MEAN}}}}$*=*mean signal from all cells in the group on ${I}_{w,z}$

for each cell ${{{\rm{c}}}}$ in the group

${X}_{c}$ = the signal from cell c on ${I}_{w,z}$

$${X}_{c,{modified}}={MEAN}-{X}_{c}.$$

if a cell is a dying cell of the group

$${Y}_{c}=1$$

else

$${Y}_{c}=0$$

Then the procedure is the same as described above. We train TSF models on balanced datasets, using various signals, using ${{{\rm{w}}}}=1{{{\rm{hr}}}}$ and various values of parameter z. We calculate the scores and do local prediction on the test datasets. We validated the reproducibility of the prediction by comparing three independent TSF predictions (showing almost no variation in the F1 score at all time windows).

### Statistics

Data were not analysed blindly. No specific method was used to predetermine the number of samples. The definition of n and the number of samples are given in each figure legend and in the table of the Experimental model section. Error bars are the standard error of the mean (s.e.m.) or 95% confidence interval. *p*-values are calculated through a t-test if the data passed the normality test (Shapiro-Wilk test), or a Mann-Whitney test/Rank sum test if the distribution was not normal. Statistical tests were performed on GraphPad Prism 8 or MATLAB.

### Reporting summary

Further information on research design is available in the Nature Portfolio Reporting Summary linked to this article.

## Supplementary information

Supplementary Information

Description of Additional Supplementary Files

Supplementary Movie 1

Supplementary Movie 2

Supplementary Movie 3

Supplementary Movie 4

Supplementary Movie 5

Supplementary Movie 6

Supplementary Movie 7

Supplementary Movie 8

Supplementary Movie 9

Supplementary Movie 10

Supplementary Movie 11

Supplementary Movie 12

Supplementary Movie 13

Supplementary Movie 14

Supplementary Movie 15

Supplementary Movie 16

Supplementary Movie 17

Supplementary Movie 18

Reporting Summary

Transparent Peer Review file

## Data Availability

All the raw data corresponding to each figure panel (including images and local projection of movies) are accessible on the following repository^91^. https://doi.org/10.5281/zenodo.20733887. A root file on the repository also describes where to find each raw data and each associated code in the repository.

## Code availability

All the code corresponding to each figure panel (including images and local projection of movies) is accessible in the following repository^91^. https://doi.org/10.5281/zenodo.20733887.
