# Supplementary material for: Short-term memory of past caspase activity biases cell elimination in vivo
Source: Nat Commun. 2026 Sep 22;17:11031. doi: 10.1038/s41467-026-77685-9 (PMC13687027; doi:10.1038/s41467-026-77685-9)
Supplement: Supplementary file 1 [file 41467_2026_77685_MOESM1_ESM.pdf]

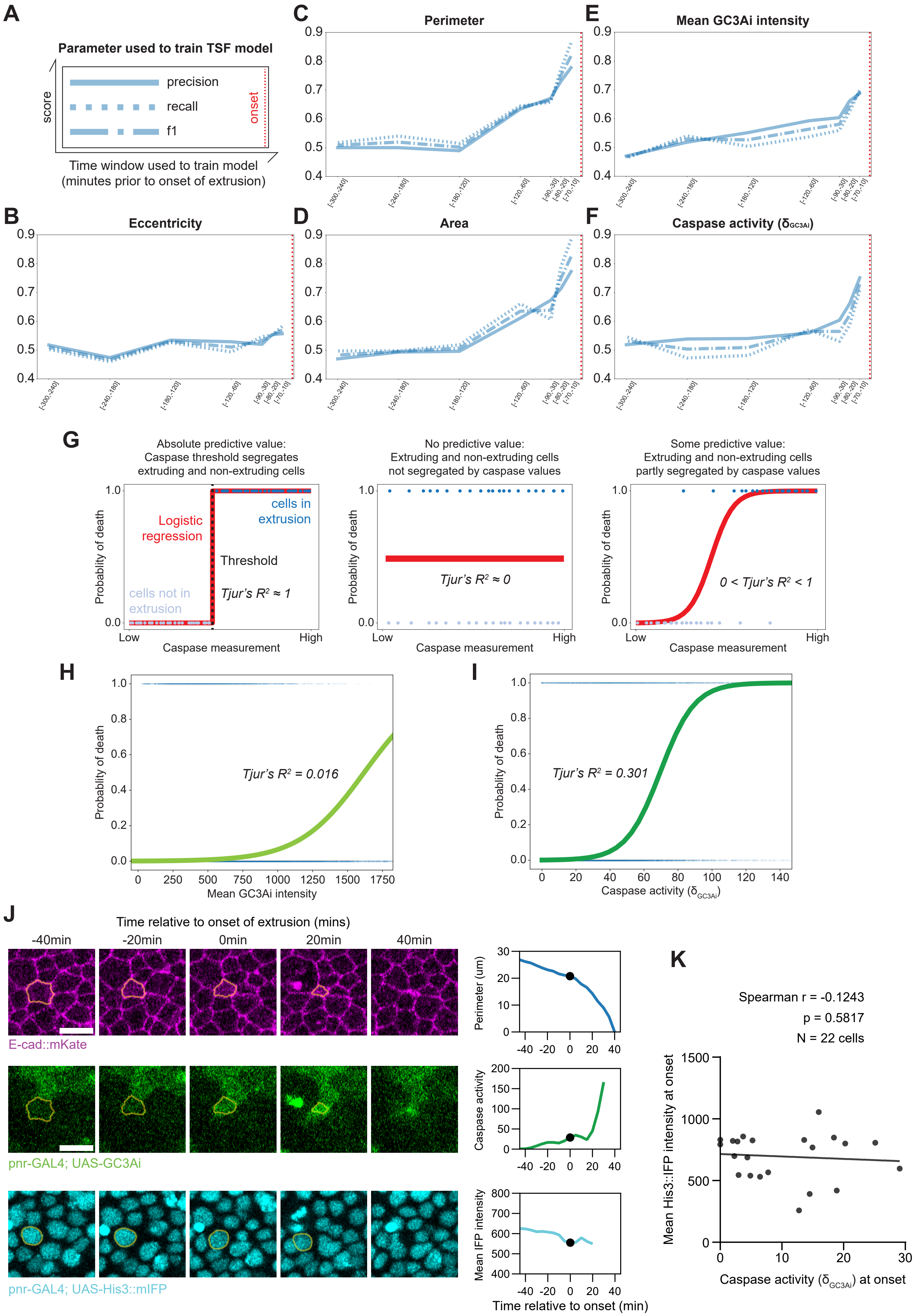

A
Parameter used to train TSF model
precision
recall
f1
onset
score
Time window used to train model
(minutes prior to onset of extrusion)
B
Eccentricity
C
Perimeter
D
Area
E
Mean GC3Ai intensity
F
Caspase activity (δGC3Ai)
G
Absolute predictive value:
Caspase threshold segregates
extruding and non-extruding cells
Logistic regression
cells in extrusion
Threshold
cells not in extrusion
Tjur's R² ≈ 1
No predictive value:
Extruding and non-extruding cells
not segregated by caspase values
Tjur's R² ≈ 0
Some predictive value:
Extruding and non-extruding cells
partly segregated by caspase values
0 < Tjur's R² < 1
Probablity of death
Caspase measurement
Low
High
H
Tjur's R² = 0.016
Mean GC3Ai intensity
I
Tjur's R² = 0.301
Caspase activity (δGC3Ai)
J
Time relative to onset of extrusion (mins)
-40min
-20min
0min
20min
40min
E-cad::mKate
pnr-GAL4; UAS-GC3Ai
pnr-GAL4; UAS-His3::mIFP
Perimeter (um)
Caspase activity
Mean IFP intensity
Time relative to onset (min)
K
Spearman r = -0.1243
p = 0.5817
N = 22 cells
Mean His3::IFP intensity at onset
Caspase activity (δGC3Ai) at onset

Supplementary Figure 1: Identification of parameters and time windows relevant for cell death prediction (associated with Figure 1).

A: Summary of the metrics shown in each graph of the Time Series Forest (TSF) prediction. Solid curve, precision, dotted curve, recall, dotted-dash curve, F1 score on the y axis (see **Methods** for explanations). x axis shows the time windows used to train the model (in minutes prior to extrusion onset, red dotted line). **B-F:** Precision, recall and F1 score of the prediction of dying cells in the pupal notum using TSF models trained on the corresponding time window (x-axis) using a dying cell and one of its neighbours as control measuring cell eccentricity (**B**), cell apical perimeter (**C**), cell apical area (**D**), GC3Ai raw intensity (**E**) and caspase activity (GC3Ai intensity derivative, **F**). The baseline (random prediction) is at 0.5. **G:** Schematic of the segregation of data based on logistic regression and sorting of the dying and non-dying cells based on caspase activity. Left shows a perfect prediction (step-like function), middle the absence of predictive value, and right a partial predictive value. **H,I:** Logistic regression of the probability of cell death as a function of raw GC3Ai intensity **(H)** and caspase activity **(I)** based on the curves shown in **Fig. 1 D** and **E**. The discrimination power is estimated using Tjur's R^2. **J:** Snapshots of a single cell undergoing cell extrusion showing E-cad::mKate (top, magenta), UAS-GC3Ai signal (middle, green) and UAS-His3::mIFP (bottom, cyan) both driven by the pnr-Gal4 driver. Time 0 is the onset of cell extrusion. Scale bars=10μm. Blue curve shows the perimeter over time, green curve the caspase activity (GC3Ai derivative) and cyan curve the His3::mIFP intensity. **K:** Correlation between caspase activity at the onset of extrusion (x-axis) and His3::mIFP intensity (y-axis). n=22 extruding cells (one dot=one extruding cell). Note the absence of correlation between the far-red signal (a proxy of Gal4 transcriptional activity) and the caspase activity at the onset, r=-0.12, p=0.58 (Pearson correlation test, two sided).

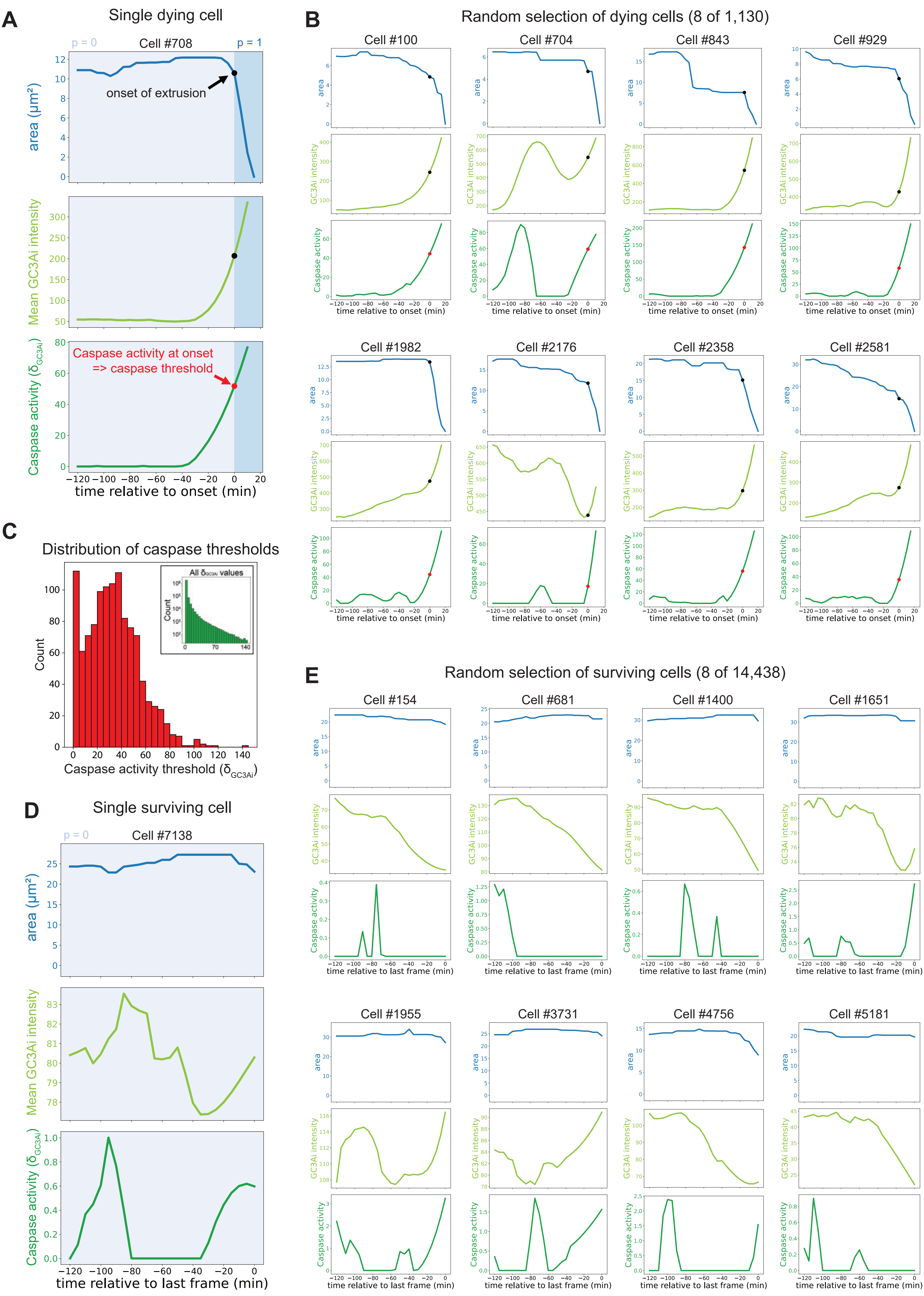
A
Single dying cell
p = 0
Cell #708
p = 1
area (μm²)
onset of extrusion
Mean GC3Ai intensity
Caspase activity (δ_{GC3Ai})
Caspase activity at onset
=> caspase threshold
time relative to onset (min)
B
Random selection of dying cells (8 of 1,130)
Cell #100
Cell #704
Cell #843
Cell #929
Cell #1982
Cell #2176
Cell #2358
Cell #2581
area
GC3Ai intensity
Caspase activity
time relative to onset (min)
C
Distribution of caspase thresholds
All δ_{GC3Ai} values
Count
Caspase activity threshold (δ_{GC3Ai})
D
Single surviving cell
p = 0
Cell #7138
area (μm²)
Mean GC3Ai intensity
Caspase activity (δ_{GC3Ai})
time relative to last frame (min)
E
Random selection of surviving cells (8 of 14,438)
Cell #154
Cell #681
Cell #1400
Cell #1651
Cell #1955
Cell #3731
Cell #4756
Cell #5181
area
GC3Ai intensity
Caspase activity
time relative to last frame (min)

Supplementary Figure 2: Systematic tracking of cell area and GC3Ai intensity allows extraction of caspase activity at the onset of extrusion for all dying cells (associated with Figure 1).

A: Line plots showing the area (top), mean GC3Ai intensity (middle) and caspase activity (δ_{GC3Ai}; bottom) of a single dying cell over time relative to the onset of extrusion. The onset of extrusion is automatically detected from the area curve (top, black dot at time = 0) using a two-line fit model (see **Methods**), and an extrusion state of p=1 is assigned to the cell from this timepoint until its apical closure, while p=0 is assigned to all prior timepoints. All measurements of GC3Ai and caspase activity, and their associated values of p, are used to populate the curves in **Fig. 1D** and **Fig. 1E**, respectively. Caspase activity at the onset of extrusion (bottom, red dot at time = 0) is assigned as the "caspase threshold" of the dying cell. **B:** Line plots showing the area (top), mean GC3Ai intensity (middle) and caspase activity (δ_{GC3Ai}; bottom) over time relative to the onset of extrusion of 8 randomly selected dying cells (from the larger dataset of n=1,130 dying cells. **C:** Histogram showing the distribution of caspase thresholds of all dying cells (n=1,130 dying cells). Inset histogram shows the distribution of all caspase activity values measured across all timepoints for all cells (dying or not dying, n=15,568 cells; 1,929,322 values of caspase activity). Note the logarithmic axis and abundance of values close to 0. **D:** Line plots showing the area (top), mean GC3Ai intensity (middle) and caspase activity (δ_{GC3Ai}; bottom) of a single surviving cell over time relative to the last timeframe of the dataset. Note that the extrusion state of the cell p is equal to 0 at all timeframes, as there is no onset of extrusion. **E:** Line plots showing the area (top), mean GC3Ai intensity (middle) and caspase activity (δ_{GC3Ai}; bottom) over time relative to the last timeframe of the dataset of 8 randomly selected surviving cells (from the larger dataset of n=14,438 non-dying cells). Note that the y-axis scale of each plot is different (much more narrow for GC3Ai/caspase activity of non-dying cells).

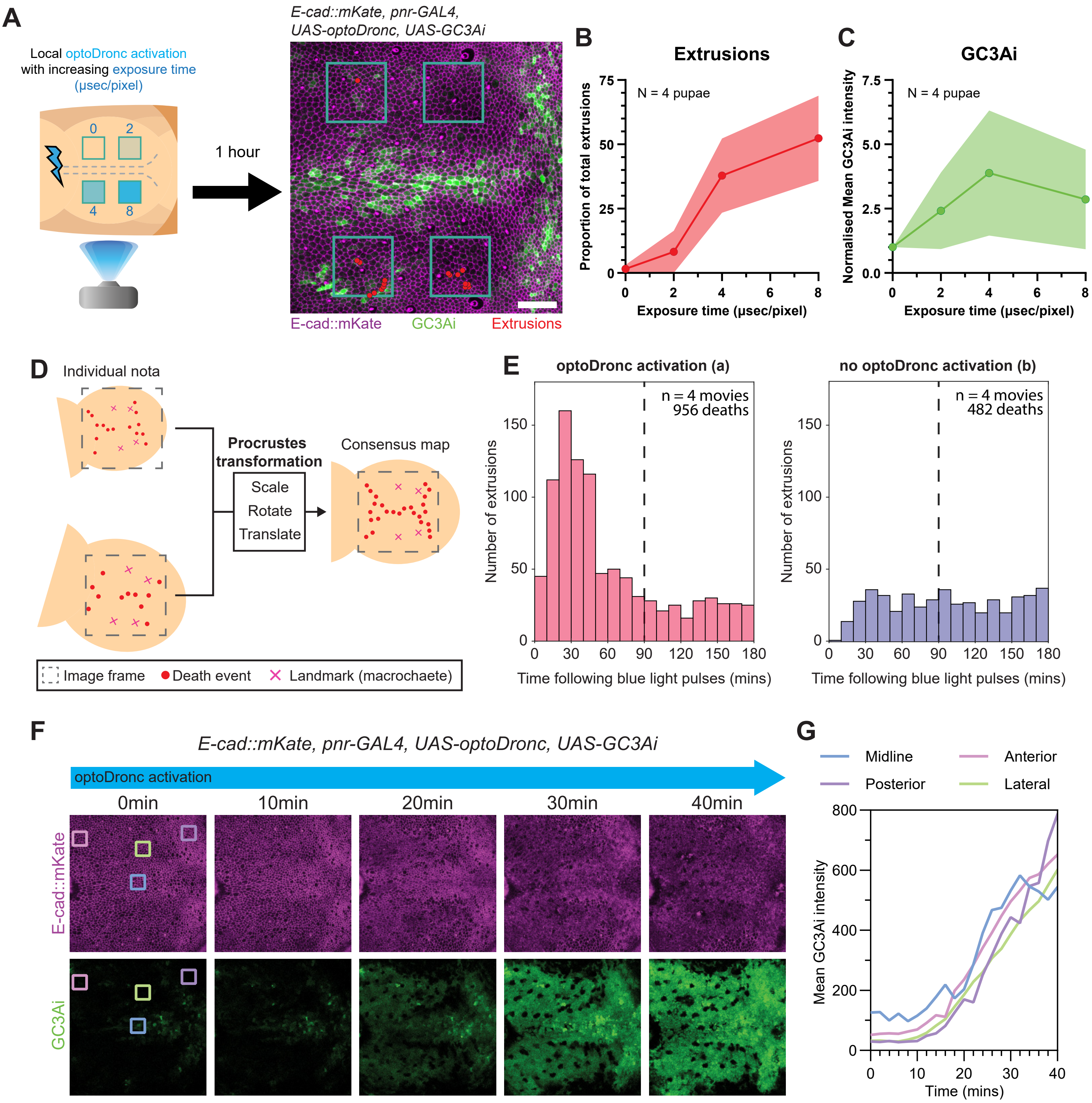

A
Local optoDronc activation
with increasing exposure time
(μsec/pixel)
0
2
4
8
1 hour
E-cad::mKate, pnr-GAL4,
UAS-optoDronc, UAS-GC3Ai
E-cad::mKate
GC3Ai
Extrusions
B
Extrusions
N = 4 pupae
Proportion of total extrusions
Exposure time (μsec/pixel)
C
GC3Ai
N = 4 pupae
Normalised Mean GC3Ai intensity
Exposure time (μsec/pixel)
D
Individual nota
Procrustes transformation
Scale
Rotate
Translate
Consensus map
Image frame
Death event
Landmark (macrochaete)
E
optoDronc activation (a)
n = 4 movies
956 deaths
Number of extrusions
Time following blue light pulses (mins)
no optoDronc activation (b)
n = 4 movies
482 deaths
Number of extrusions
Time following blue light pulses (mins)
F
E-cad::mKate, pnr-GAL4, UAS-optoDronc, UAS-GC3Ai
optoDronc activation
0min
10min
20min
30min
40min
E-cad::mKate
GC3Ai
G
Midline
Anterior
Posterior
Lateral
Mean GC3Ai intensity
Time (mins)

Supplementary Figure 3: Susceptibility to caspase is spatially patterned (associated with Figure 1 and Figure 2).

A: Left, graded activation of optoDronc in the pupal notum by exposing regions of the lateral domains (blue boxes) to blue light for increasing amounts of time (0, 2, 4 and 8 μsec/pixel). Right, snapshot of a notum expressing *UAS-optoDronc* (no GFP tag) and *UAS-GC3Ai* with *pnr-Gal4* 1 hour following initial optoDronc activation showing cumulative caspase activity via GC3Ai intensity, and extrusions which occurred in the intervening hour marked by red dots. n=4 pupae, scale bar=50μm **B:** Proportion of total extrusions of the notum (%) occurring in 1 hour following optoDronc activation in each regions subjected to increasing blue light exposure. Shaded region: s.e.m.. n=4 pupae. **C:** Normalised mean GC3Ai intensity 1 hour following optoDronc activation in regions subjected to increasing blue light exposure. Shaded region: s.e.m.. n=4 pupae. **D:** Process used to produce a consensus spatial map of optoDronc-induced deaths. Procrustes transformation is used to align, scale and rotate pupae and their corresponding mapped extrusions using 4 posterior macrochaetae (aDC and pDC macrochaetae) as spatial landmarks (pink crosses). **E:** Histogram of the total number of cell extrusions counted at various time windows (minutes post-blue light activation) in *pnr-Gal4 UAS-optoDronc* pupae following mild and global activation of optoDronc (left, (a)) or in absence of blue light (right, (b)) (associated with **Fig. 1F**,**G**). n=4 pupae, 956 cell death after blue light activation, 482 in the controls. Additional deaths observed in the blue light-activated pupae mostly occur in the first 90 minutes (black doted lines). **F:** Snapshots of a pupal notum expressing *UAS-optoDronc* (no GFP tag) and *UAS-GC3Ai* with *pnr-Gal4* upon global and persistent blue light exposure. E-cad::mKate in magenta, GC3Ai in green. Note the global and homogeneous increase of GC3Ai signal (despite the initial heterogeneity corresponding to the normal pattern of caspase activation). The coloured squares show the regions of intensity measurement quantified in **G**. Scale bars=50μm. See also **Supplementary Movie 4**. Anterior, left, posterior, right. **G:** Time evolution of GC3Ai intensity in different ROIs of the pupae shown in **F**, blue: midline, pink: anterior, purple: posterior, green: lateral.

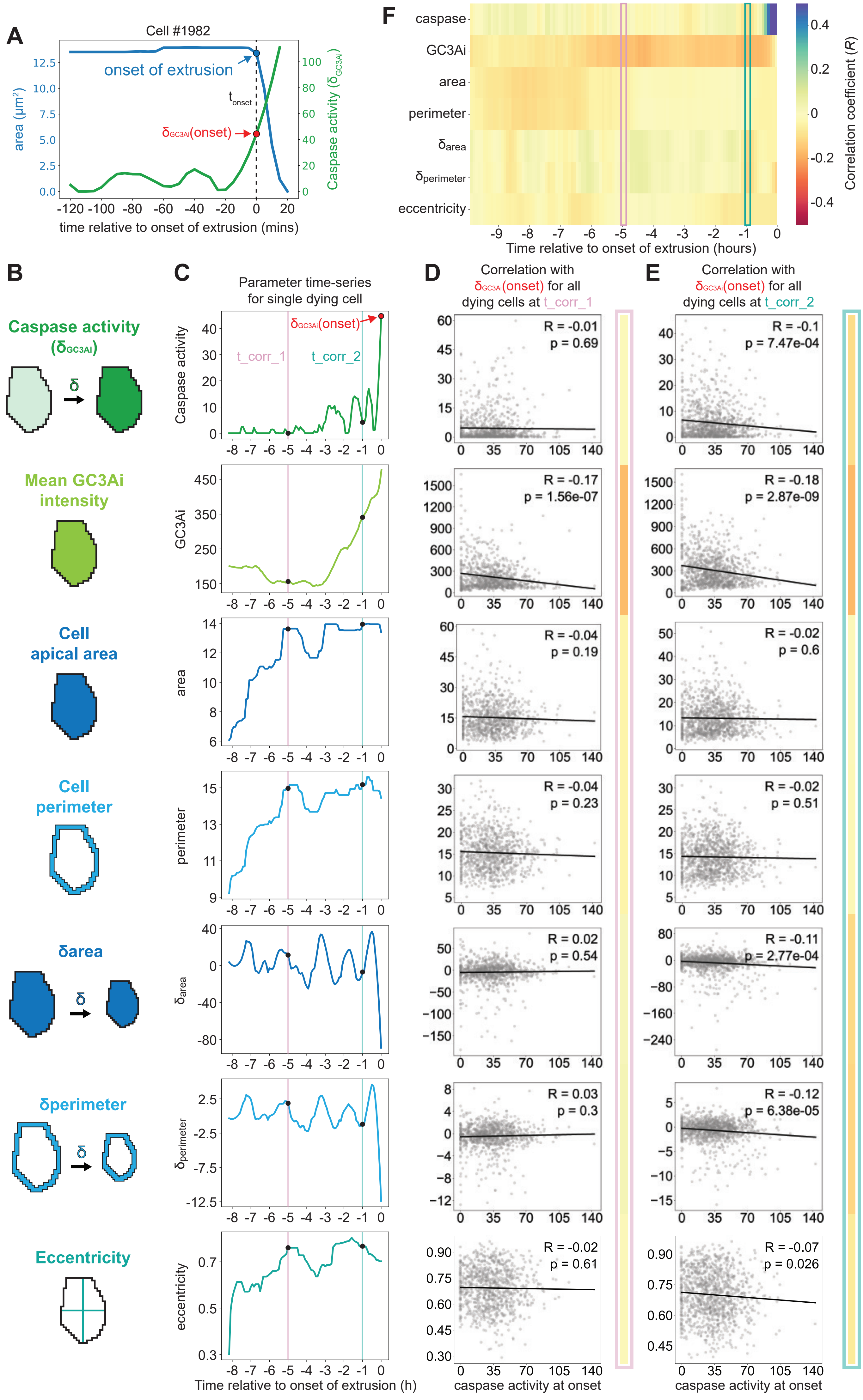

A
Cell #1982
onset of extrusion
t_onset
δGC3Ai(onset)
area (μm²)
Caspase activity (δGC3Ai)
time relative to onset of extrusion (mins)
F
caspase
GC3Ai
area
perimeter
δarea
δperimeter
eccentricity
Time relative to onset of extrusion (hours)
Correlation coefficient (R)
B
Caspase activity (δGC3Ai)
Mean GC3Ai intensity
Cell apical area
Cell perimeter
δarea
δperimeter
Eccentricity
C
Parameter time-series for single dying cell
δGC3Ai(onset)
t_corr_1
t_corr_2
Caspase activity
GC3Ai
area
perimeter
δarea
δperimeter
eccentricity
Time relative to onset of extrusion (h)
D
Correlation with δGC3Ai(onset) for all dying cells at t_corr_1
R = -0.01 p = 0.69
R = -0.17 p = 1.56e-07
R = -0.04 p = 0.19
R = -0.04 p = 0.23
R = 0.02 p = 0.54
R = 0.03 p = 0.3
R = -0.02 p = 0.61
caspase activity at onset
E
Correlation with δGC3Ai(onset) for all dying cells at t_corr_2
R = -0.1 p = 7.47e-04
R = -0.18 p = 2.87e-09
R = -0.02 p = 0.6
R = -0.02 p = 0.51
R = -0.11 p = 2.77e-04
R = -0.12 p = 6.38e-05
R = -0.07 p = 0.026
caspase activity at onset

Supplementary Figure 4: Correlation analysis of all dying cells highlights a negative association between caspase δ_{GC3Ai}(onset) and past GC3Ai intensity (associated with Figure 2).

A: Area (blue) and caspase activity (δ_{GC3Ai}; green) of a single dying cell over time relative to the onset of extrusion (t_{onset}; black dotted line). The onset of apical constriction (blue dot) is automatically detected from the area curve and used to define δ_{GC3Ai}(onset), the caspase threshold of the cell (red dot). **B:** Morphometric and caspase-related parameters measured for all dying cells prior to the onset of extrusion, and used for the correlation analysis shown in **F**. **C:** Examples of time course of each parameter shown in **B** for a single dying cell (cell #1982 shown in **A**). For caspase activity (δ_{GC3Ai}; top), the caspase threshold of the cell, δ_{GC3Ai}(onset), is shown with a red dot. In all plots, vertical coloured lines are used to highlight two time windows used to extract the cloud plots of **D** and **E** (t_corr_1, pink line at t = -5h; t_corr_2, cyan line at t = -1h). **D,E:** Cloud plots of δ_{GC3Ai}(onset) of all dying cells against the values of each parameter shown in **B** at t_corr_1 (5h prior to the onset of extrusion) (**D**) or t_corr_2 (1h prior to the onset) (**E**). Linear correlation is plotted with a black line, Pearson correlation coefficient (R) and associated p-value (two sided Pearson correlation test) are shown in the top right of the plot. The strength of the correlation measured from each cloud plot is illustrated by coloured bars to the right of the plots which are used to populate the matrix in **F** (colour bar scale also shown in **F**). n=927 dying cells for **D** and 1,094 for **E** , 2 nota. **F:** Matrix from **Fig. 2C** showing correlations between δ_{GC3Ai}(onset) and the parameters illustrated in **B** in windows of time prior to the onset of extrusion. Blue: positive correlation, Red: negative correlation. Pink and cyan vertical boxes highlight the values of the matrix which are populated by Pearson correlation coefficients generated using parameter measurements at t_corr_1 (5h prior to the onset of extrusion, **D**) and t_corr_2 (1h prior to the onset of extrusion, **E**), respectively. n=1,130 dying cells, 2 nota.

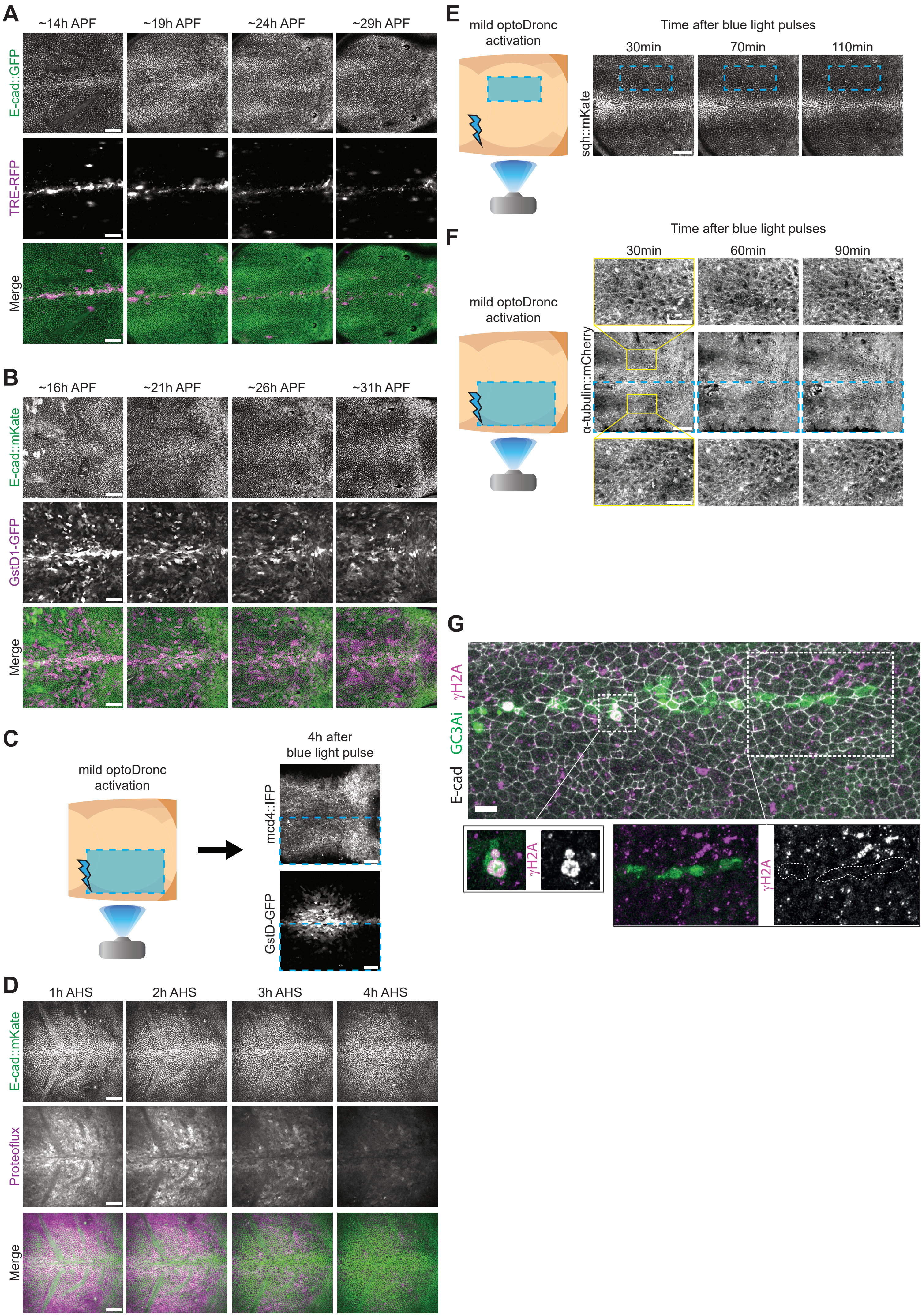
A
~14h APF
~19h APF
~24h APF
~29h APF
E-cad::GFP
TRE-RFP
Merge
B
~16h APF
~21h APF
~26h APF
~31h APF
E-cad::mKate
GstD1-GFP
Merge
C
mild optoDronc activation
4h after blue light pulse
mcd4::IFP
GstD-GFP
D
1h AHS
2h AHS
3h AHS
4h AHS
E-cad::mKate
Proteoflux
Merge
E
mild optoDronc activation
Time after blue light pulses
30min
70min
110min
sqh::mKate
F
mild optoDronc activation
Time after blue light pulses
30min
60min
90min
α-tubulin::mCherry
G
E-cad GC3Ai γH2A
γH2A
γH2A

Supplementary Figure 5: Testing putative regulators of the memory of past caspase activation (associated with Discussion).

A: Pupal notum expressing JNK reporter TRE-RFP (middle, magenta) and E-cad::GFP (top, green) at different developmental times (hours After Pupal Formation, APF). Note the absence of signal in the posterior region. Scale bar=50μm. Representative of 2 pupae. **B:** Pupal notum expressing the ROS reporter GstD1-GFP (middle, magenta) and E-cad::GFP (top, green) at different developmental times (hours After Pupal Formation, APF). No clear signal upregulation in the posterior region. Scale bar=50μm. Representative of 3 pupae. **C:** Pupal notum expressing GstD1-GFP (greyscale) and *pnr-Gal4 UAS-optoDronc* 4 hours after blue light activation in the left region (schematic, doted blue rectangle). Note that GstD1-GFP transcriptional reporter should perdure for hours upon activation. Scale bar=50μm. Representative of 2 pupae. **D:** Pupal notum expressing the proteasome activity reporter (ProteoFLUX, hs-CL1::GFP, magenta) and E-cad::mKate (top, green) over time after heat shock induction of the proteasome reporter (hours After Heat Shock, AHS). Note the absence of pattern of intensity decrease (a proxy of proteasome activity), especially in the posterior region. Scale bar=50μm. Representative of 7 pupae. **E:** Pupal notum expressing sqh::mKate (greyscale) and *pnr-Gal4 UAS-optoDronc* upon mild activation of caspase in the right region of the notum (see left schematic, doted blue rectangle) over time after blue light activation (minutes). Scale bar=50μm. Representative of 4 pupae. **F:** Pupal notum expressing UAS-α-tub::mCherry (greyscale) and *pnr-Gal4 UAS-optoDronc* upon mild activation of caspase in the left region of the notum (see left schematic, doted blue rectangle) over time after blue light activation (minutes). Yellow rectangles are shown above and below. Scale bar=50μm in main image, 20μm in insets. Representative of 2 pupae. **G:** z-projection of an immunostaining of a pupal notum expressing GC3Ai in the pnr domain (green, native fluorescence) showing the midline region (line of GC3Ai positive cells) after staining for γ-H2AX (DNA damage, magenta) and E-cad (white). Left bottom inset: basal apoptotic debris positive for DNA damage. No change of γ-H2AX in the rest of the midline, even in the cells positive for GC3Ai still in the epithelial layer (bottom right inset). Scale bar=10μm. Representative of 5 pupae. For all panels, Anterior, left, posterior, right.

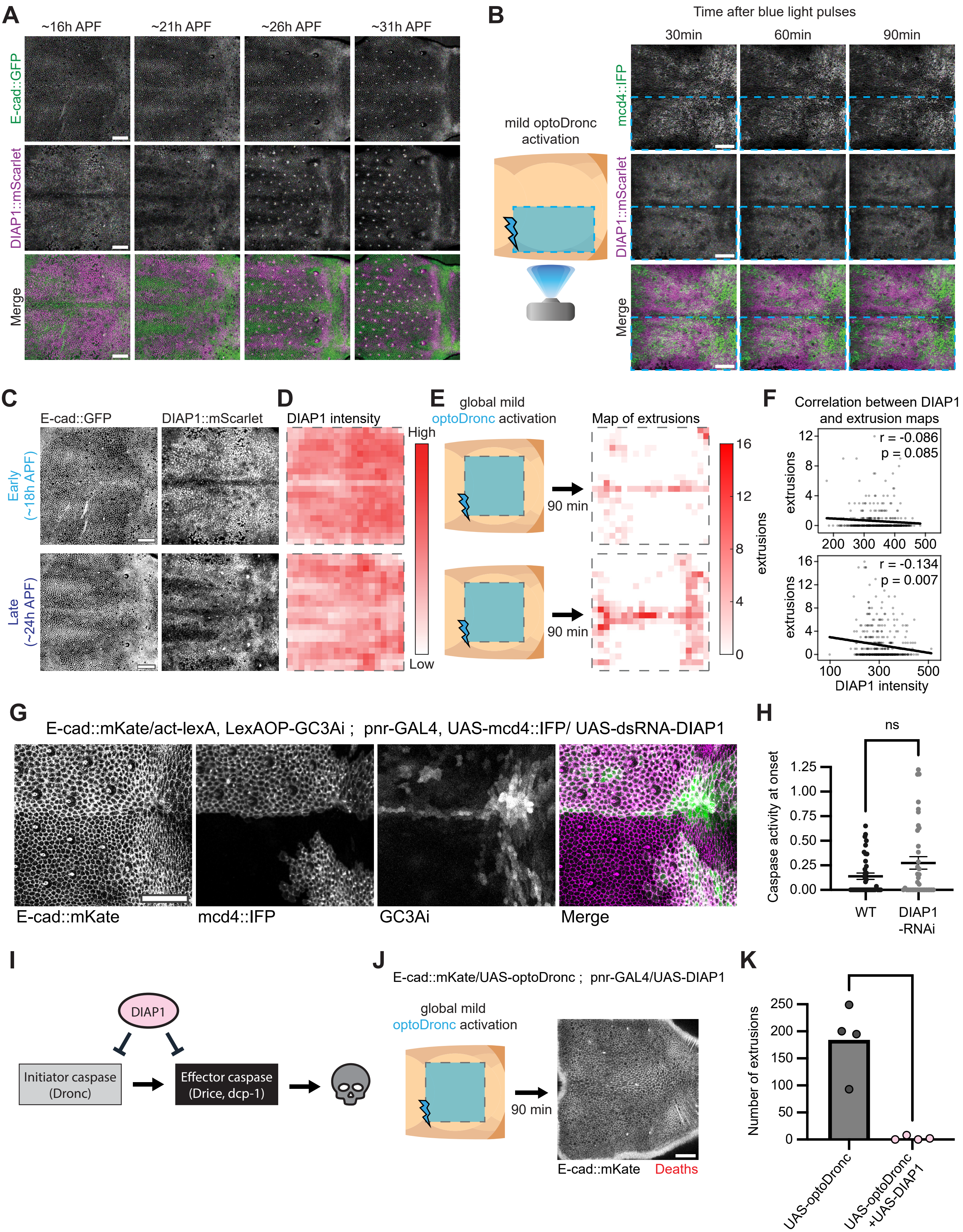
A
~16h APF
~21h APF
~26h APF
~31h APF
E-cad::GFP
DIAP1::mScarlet
Merge
B
Time after blue light pulses
30min
60min
90min
mild optoDronc activation
mcd4::IFP
DIAP1::mScarlet
Merge
C
E-cad::GFP
DIAP1::mScarlet
Early (~18h APF)
Late (~24h APF)
D
DIAP1 intensity
High
Low
E
global mild optoDronc activation
90 min
Map of extrusions
16
12
8
4
0
extrusions
F
Correlation between DIAP1 and extrusion maps
r = -0.086
p = 0.085
r = -0.134
p = 0.007
extrusions
DIAP1 intensity
G
E-cad::mKate/act-lexA, LexAOP-GC3Ai ; pnr-GAL4, UAS-mcd4::IFP/ UAS-dsRNA-DIAP1
E-cad::mKate
mcd4::IFP
GC3Ai
Merge
H
ns
Caspase activity at onset
WT
DIAP1 -RNAi
I
DIAP1
Initiator caspase (Dronc)
Effector caspase (Drice, dcp-1)
J
E-cad::mKate/UAS-optoDronc ; pnr-GAL4/UAS-DIAP1
global mild optoDronc activation
90 min
E-cad::mKate
Deaths
K
Number of extrusions
UAS-optoDronc
UAS-optoDronc +UAS-DIAP1

Supplementary Figure 6: Diap1 protein level is not sufficient to explain the caspase memory effect (associated with Discussion).

A: Pupal notum expressing the knock-in of Diap1::mScarlet (magenta, middle) and E-cad::GFP (top, green) at different developmental times (hours After Pupal Formation, APF). Scale bar=50μm. Representative of 2 pupae. **B:** Diap1::mScarlet (middle, magenta), *pnr-Gal4 UAS-optoDronc* and UAS-mCd4::mIFP (top, green) at different time (minutes) after mild activation of caspase in the left region (dotted blue rectangle). Scale bar=50μm. Representative of 2 pupae. **C:** E-cad::GFP (left) and Diap1::mScarlet (right) at early (~18h APF, top) and late (~24h APF, bottom) developmental stages. Scale bars=50μm. Representative of 2 pupae. **D:** Map of Diap1::mScarlet intensity in early (top) and late (bottom) nota from **C**. **E:** Averaged maps of extrusions over 90 minutes at early (top) and late (bottom) developmental stage after mild global optoDronc activation and Procrustes alignment with the notum used for DIAP1 intensity map. n=3 pupae and 238 total extrusions for early and n=3 pupae and 644 total extrusions for late stages. **F:** Spatial correlation cloud plots of local DIAP1 intensity and local rate of extrusion induced by optoDronc in early (top) and late (bottom) pupae. One point=one xy box. Black lines: linear correlation plots, Pearson correlation coefficient (r) and p-value (Pearson test, two sided) shown on the top right. **G:** Pupal notum expressing E-cad::mKate (magenta), ubiquitous GC3Ai (act-lexA, lexAOP-GC3Ai, green), dsRNA against diap1 and mcd4::IFP in mitotic clones (white). Note the ectopic caspase activation in the clones (top left). Scale bar = 50μm. **H:** Caspase activity at the onset of extrusion of WT and DIAP1-depleted cells. One point=one dying cell, central bar: mean, error bars: s.e.m.. Mann-Whitney test, p-value=0.2331. n=2 movies; 38 WT and 37 DIAP1-RNAi dying cells. **I:** DIAP1 epistasis. **J:** Global mild optoDronc activation in a *pnr-Gal4 UAS-diap1* background. Red dots: cell death occurring over 90min after blue light pulse. Scale bar = 50μm. **K:** Total number of extrusions over 90 minutes after mild optoDronc activation in a control (left) and DIAP1-overexpression context (right). One point= one notum. Bars: mean, error bars: s.e.m.., Mann-Whitney Test, p-value = 0.0286. n=4 UAS-optoDronc nota, 4 UAS-optoDronc + UAS-DIAP1 nota. For all panels, anterior left, posterior right.
