# Supplementary material for: Short-term memory of past caspase activity biases cell elimination in vivo
Source: Nat Commun. 2026 Sep 22;17:11031. doi: 10.1038/s41467-026-77685-9 (PMC13687027; doi:10.1038/s41467-026-77685-9)
Supplement: Supplementary file 3 [file 41467_2026_77685_MOESM22_ESM.pdf]

nature portfolio

Peer Review File

Short-term memory of past caspase activity biases cell elimination in vivo

Corresponding Author: Dr Romain Levayer

This file contains all reviewer reports in order by version, followed by all author rebuttals in order by version.

Version 0:

Reviewer comments:

Reviewer #1

(Remarks to the Author)
This work by Levayer and colleagues investigates how cells decide to live or die following caspase activation in the Drosophila pupal notum epithelium. Traditionally, effector caspase activation was considered a point of no return in apoptosis. However, evidence provided in the last 10 years has suggested that sublethal caspase activity is common and may serve non-apoptotic roles. The authors here aim to understand how cells integrate caspase activity over time to make life-or-death decisions.

Using a combination of live imaging and machine learning, the authors performed a quantitative single-cell analysis in a live tissue context, the pupal notum. Using this approach, the authors demonstrate that there is a heterogeneity in caspase thresholds for apoptosis across cells in the pupal notum. Machine learning showed that caspase activity is predictive of apoptosis, but with significant variability, which suggests additional regulatory layers. They then showed that prior sublethal caspase activation lowers the threshold for future apoptosis. Optogenetic induction of caspase activity (optoDronc) confirms that cells that were previously exposed to caspases, are more likely to die upon reactivation. Furthermore, they showed that spatial patterns of apoptosis susceptibility correlate with past caspase activity. Depleting the pro-apoptotic gene hid flattens these spatial patterns (although does not completely erase it), confirming the role of past caspase exposure. Finally, the authors demonstrate that cells with a history of caspase activation are more likely to be eliminated when competing with neighbors. This introduces a non-autonomous, history-dependent mechanism of cell competition, which could influence clonal selection during development and potentially in tumorigenesis.

This study identifies a novel regulatory layer in apoptosis decision-making whereby past sublethal caspase activity primes cells for future elimination. This finding redefines caspase activity as a dynamic and memory-encoded input that contributes to tissue patterning and cell competition. The figures clearly support the major claims. I think these findings have important implications for our understanding of apoptosis regulation in developmental biology, cell competition and disease.

While this work is very compelling, I still have a few comments, concerns and questions.

1. A major weakness of the work is that the molecular mechanism underlying the memory of past caspase activity remains unidentified. I recognize that the authors have examined some possibilities, without success. I hope it is a long-term goal of the authors to address this question.

2. Some conclusions rely on correlation (e.g., high GC3Ai signal correlates with future apoptosis) rather than direct causation. It is desirable to directly test causality of caspase priming. Use optoDronc to deliver a sublethal caspase pulse, then block caspase activity genetically or pharmacologically and apply a second pulse. This experiment may also determine whether the priming effect is reversible and whether it requires sustained caspase activity or downstream signaling.

3. How long is optoDronc stable and active after the pulse?

4. GC3Ai may not perfectly reflect real-time caspase activity due to its accumulation and stability, which may over-represent low, transient caspase activity and potentially mask fast dynamics. In fact, GC3Ai's cumulative nature may obscure the precise timing of caspase activation. A sensor with faster turnover or cleavage specificity might improve temporal resolution. If available, use of a faster-turnover caspase sensor might be better suited to capture rapid fluctuations of caspase activity

and thus may better define the duration and decay of the caspase "memory."

5. Along those lines, is there a caspase sensor with reversible activation kinetics to differentiate between transient and sustained caspase signaling which could be used here?

6. The manuscript would benefit from a clear schematic model summarizing the proposed model of how past and current caspase activities are integrated to determine cell fate.

7. Do caspase-primed cells exhibit changes in mechanical properties (e.g., tension, stiffness) that might influence extrusion?

8. Does apoptotic memory affect epithelial repair, regeneration, or barrier function?

9. In the experiment in Figure 2D, the authors exposed the lateral domain of the midline to optoDronc twice and the contralateral region as a control. Could the authors perform the reverse experiment - exposing the contralateral region to optoDronc twice while using the lateral domain of the midline as a control - to rule out potential position effects?

10. Although hid is the primary apoptosis-inducing gene in the pupal notum, hid RNAi fails to completely eliminate caspase activity. This failure might simply reflect inefficient knockdown of hid. Alternatively, reaper and grim may also be (minor) contributors to apoptosis induction. To test this, the authors could simultaneously target all three genes using an available RNAi line that targets hid, reaper, and grim together, then assess whether this produces a stronger effect on caspase activity.

11. In the Discussion, lines 360-365: Shlevkov and Morata (2012) discussed a positive feedback loop in dying cells. Maybe the authors can follow up on their work and further examine this loop.

12. The authors frequently use the phrase "for the first time," which is problematic for several reasons. First, most scientific findings are by definition novel, making this phrase redundant. Second, it's difficult to definitively claim “for the first time” given the vast body of existing literature. For instance, while the authors claim to have demonstrated heterogeneity in caspase thresholds for apoptosis across cells for the first time (Figure 1), Fan and Bergmann (2014) made similar observations in the eye imaginal disc, and M. Miura's group made comparable findings in Koto et al (2009). The authors should moderate their language and acknowledge these prior studies in their discussion.

13. Along these lines, both Fan and bErgmann and Koto et al. identified varying Diap1 protein levels as the basis for differential cellular sensitivity to apoptotic signals. Looking at Suppl fig S4H, there is variation in Diap1 levels. Interestingly, reduced Diap1 expression is reduced in regions of high apoptosis within the pupal notum. This could account for the heterogeneous cellular responses to uniform caspase input (optoDronc) and may also explain the effects of past caspase activity. The authors should investigate this further by experimentally manipulating Diap1 levels.

Reviewer #2

(Remarks to the Author)
In this manuscript, Cumming at al. investigate whether past sublethal caspase activity can influence the likelihood of a cell to undergo apoptosis in the Drosophila pupal notum epithelium. Through live imaging and machine learning, they reveal large variability in caspase sensitivity across cells, and identify past caspase activation as a correlated factor potentially contributing to this heterogeneity. Optogenetic activation of caspases increased cell extrusion events, supporting the role of past caspase activation in determining cell extrusion.

While the manuscript presents an interesting hypothesis regarding the role of past caspase activity in priming cells for apoptosis, the major claims are not sufficiently supported by the data presented. Specifically, two major claims: 1) that caspase thresholds vary across cells, and 2) that cells integrate past caspase activity to modulate sensitivity to apoptosis, are neither clearly defined nor experimentally validated. Several conclusions rely on correlative observations without demonstrating causality. Moreover, the study does not distinguish its proposed model from simpler alternative explanations (see Point #3). In its current form, the manuscript lacks necessary evidence and mechanistic insights needed to support its major claims. For these reasons, we cannot recommend the manuscript for publication.

1. The authors are encouraged to improve their data presentation. The manuscript presents processed metrics (e.g., correlation and probabilities) without showing the accompanying raw data, which limits reader’s ability to independently evaluate the validity of the claims.
1.1 To support the claim of heterogeneous caspase thresholds, the quantification of area, mean GC3Ai intensity, and caspase activity (as shown in the lower panel of Fig. 1C) should be provided for multiple cells. Ideally, this analysis should include all the cells quantified to generate Figures 1D & 1E to convincingly demonstrate heterogeneity.
1.2 In Figure 2C, the accompanying time-series data of various morphometric and caspase-related parameters need to be shown to clarify the basis of the reported correlations.
1.3 The study only reports standard error to indicate confidence in the results. The standard error is expected to be small due to the large number of cells. To demonstrate and support caspase heterogeneity, the authors should report the standard deviation instead, or in addition to standard error.

2. The central claim of the study that prior induction of caspase activity lowers the caspase threshold for cell extrusion and prime cells for apoptosis (lines 199-200) is not adequately support by data:

2.1 Caspase thresholds were not defined or quantified in the results.
2.2 The experiments in Figure 2D and Figure 3 do not establish a causal link between past GC3Ai levels and subsequent cell extrusion. To demonstrate causality, GC3Ai levels need to be monitored in real-time from optoDronc activation until cell extrusion. Importantly, applying different levels of optoDronc activation can test whether modulating GC3Ai levels can change the caspase threshold, if there is indeed a causal relationship.

3. The study does not distinguish its proposed model, where past caspase activity is integrated to modulate cell extrusion caspase threshold, from a simpler alternative model where initial sublethal activation induces a persistent state of unresolved cellular damage or stress, which sensitizes cells to subsequent apoptosis. Prior studies have shown that sublethal caspase activity can lead to altered cell fates and phenotypes. The current data do not differentiate between regulated past caspase integration and persistent cellular stress or damage. These two models can be distinguished by monitoring GC3Ai levels in real-time after optoDronc activation and measurements of markers of cellular stress or damage (e.g., γH2AX) in the several hours preceding cell extrusion.

4. The data shown in Figure 2E are inconsistent with results in Figure 2C. GC3Ai levels show negative correlation with caspase activity up to 6 hours prior cell extrusion (Fig. 2C). However, two pulses of caspase stimulation separated by 5 hours do not result in an increased % of extrusions.

5. The paper lacks comprehensive validation of the GC3Ai sensor's sensitivity and dynamic range in the specific context of the Drosophila pupal notum. Without specific validation, it's unclear whether the observed variability in caspase activity reflects biological differences.

6. The authors present a TSF model to classify dying vs. non-dying cells. However, the results partially conflict with the claim that past activation of GC3Ai primes cells for death. TSF performance is lower than 60% for 1 hour before onset (Figure S1E). If past GC3Ai intensity is predictive of apoptosis, the model should be able to capture this or else raising doubts about the model. To clarify this, the authors should (1) compare to simpler baseline models (e.g., logistic regression or decision trees at individual time points) and (2) test performance on time-shuffled data.
Moreover, key aspects of model development are missing. The authors should: (1) include cross-validation to assess generalizability; (2) conduct repeats for random sampling of non-dying cells to check model robustness; and (3) report ‘specificity’ to evaluate false positive rates, which complements their focus on identifying dying cells.

7. The term “probability” is used inconsistently throughout the manuscript, referring at different sections to the proportion of dying cells, probability from the TSF model or logistic regression, or death rates. The authors should use method-specific terminology.

Reviewer #3

(Remarks to the Author)
I co-reviewed this manuscript with one of the reviewers who provided the listed reports. This is part of the Nature Communications initiative to facilitate training in peer review and to provide appropriate recognition for Early Career Researchers who co-review manuscripts.

Reviewer #4

(Remarks to the Author)

Summary

The manuscript of Cummiong et al. presents an intriguing hypothesis that past caspase activity in otherwise viable cells, biases the induction of cell death at future timepoints in vivo. This manuscript provides a critical test of the caspase activity threshold and demonstrates that activity alone is not a sole arbiter of apoptosis, rather is a factor along with past activity itself, which primes developing cells for eventually apoptotic/programmed cell death. This manuscript makes use of state of the art imaging and real time reporter systems in an in vivo setting (drosophila) to establish the role of caspase activity ‘history’ as a death determinant, a set of observations that will be compelling to a large number of cell and developmental biologists, as well as those biochemists that are interested in the temporal control over caspase activity and cell death onset.

The GC3Ai sensor combined with machine learning is a powerful method to track in vivo caspase activity exposure. The curation of caspase activity bursts using this approach is a major advance over prior protease activity assays. Figure 1E shows a relatively narrow band of death ‘probability’ which fits with the tightly regulated process of apoptosis, which is further validation for the assay. The use of extrusion as a proxy for late stage death is also a valid measure and aligns quite well with reporter activity across a subset of the caspase activated cell population. Moreover, the real time videos provide compelling data support for the hypothesis as articulated by the authors. Other individual experiments are quite compelling, for example the data within Figure 3A and 3B provide strong support for past caspase activation and eventual cell death (although 3B could be buttressed by more supporting observations, see below). Figure 4 is equally compelling in terms of ‘neighbor’ effects. Prior to publication the authors should address a number of points as noted below.

Corrections

Regarding the abstract. The abstract is somewhat obtuse when explaining the dynamics of caspase activity and cell death decision making. The authors introduce the threshold concept, yet the abstract walks the reader away from this notion entirely. Despite the focus on past sublethal capase activation as the bias for cell death/elimination, the authors data in Figure 4A,B in fact suggest that a caspase activity threshold may exist along with past exposure bias. Given this issue the authors should acknowledge this nuance in the abstract, as the current version indicates to the reader that activity thresholds are not a contingent factor at all.

Regarding comments in the introduction. The authors state that nonapoptotic links between MOMP, mitochondrial activation of caspase cascades is a recent phenomenon over the past decade, linked largely to caspase 3 activity in resistant cancer cells. The authors should actually acknowledge the work of the labs that predate these observations considerably which include the following papers showing nonapoptotic outcomes for mitochondrial involvement in nonapoptotic caspase function across a number of cell phenomenon, i.e. Arama et al 2003, Weber and Menko 2005, Putinski et al. 2013.

Regarding Supp Figure 1J. The aligned image does not appear to highlight an area of measurable caspase activity or an intact cell per se. The authors should provide images that align with the modeling predictions.

Regarding Figure 1. While I do agree that the gross variability is caspase activity is not predictive, this does not exclude the possibility that variation in regional caspase activity may be determinative, i.e. subcellular regions where protease activity is a threshold signal for cell fate/apoptosis. That is not really addressed in the manuscript. Given the novelty of the observations I am of the opinion that this should be addressed in future research efforts, however the authors should temper their interpretations somewhat or acknowledge this issue, perhaps in the discussion.

Regarding Figure 2D,E. This data is essentially the heart of the argument, i.e. the authors suggest that the use of the optoDronc activatable construct is consistent with recent past caspase activity being determinant for death. At this stage the variability in the data in Figure 2E is suggestive but certainly not definitive to support the 'past activity means death' hypothesis. Given the variability in the dataset, and to support the core argument the authors should provide time points on either side of the 1-5 hour window, 30 mins past activation 10 hours past activation. If the hypothesis is correct one would anticipate a dramatic difference in the bar graphs from what is observed in Figure 2D,E.

Regarding Figure 3B,C, I do agree with the authors that the data is generally supportive of the hypothesis, however the limitation here is a single point in time with the hid-RNAi experiment. Why did the authors opt for a late stage interference experiment and not an early experiment as well at 18hrs? An earlier ablation of hid would strengthen the conclusions to a significant degree. The data in Figure 3D,E may actually suffice for this request, however it is not clear at what point the combined optoDronc and GC3Ai experiment was initiated in the developmental window? Some clarity here could diminish the need for another timepoint as noted above. A slightly modified version of movie S8 would also be helpful here (slower track) to visualize the overlay in signals which move to quickly.

Regarding Suppl Figure 3. The authors should consider placing this as a main figure (stand alone figure) as this is strong support for the generality of their model (another cell type) regarding caspase induced death and susceptibility to past caspase activity.

Regarding Suppl Figure 4. I do not understand the rationale for including this data. All of the assays and their targets do not appear to modify the past caspase activity and links to follow on death induction. Certainly a discussion of these targets is warranted by inclusion of a number of individual experiments do not provide definitive support or exclusion for these various modifiers. Indeed the authors themselves note that these candidates could operate in tandem or combination to effect the caspase activity sensitization step. Unless the authors want to actually address this issue directly with additional experimentation, they would be better served not presenting this preliminary data, which does not really allow for firm conclusions in its present state.

Version 1:

Reviewer comments:

Reviewer #1

(Remarks to the Author)
the revised manuscript has clearly improved. The authors have added new experimental data, provided better quantifications of timing and reversibility of caspase priming, improved the validation of the GC3Ai sensor, and also added additional negative data (DNA damage, ROS, protasome activity, cytoskeleton) that strengthen the conclusion that priming is not simply explained by known pathways. These revisions meaningfully strengthen the central claims and address several concerns raised during review.

However, regarding the last point raised in my first review, I appreciate the efforts of the authors to address my comment regarding a potential role of Diap1 in heterogeneity of the apoptosis threshold, including the generation and analysis of the Diap1::mScarlet knock-in line. These data provide useful insight into the spatial patterning of Diap1 protein levels in the

pupal notum and clarify that Diap1 does not appear to encode the short-term memory of past caspase activity. However, my original point was that cell-intrinsic differences in Diap1 protein levels could contribute to the heterogeneous caspase thresholds observed across epithelial cells, rather than to caspase memory per se. While the current data are correlative, a direct test of causality would require experimental manipulation of Diap1 levels. I therefore encourage the authors to consider reducing Diap1 dose heterozygously and, conversely, overexpressing Diap1 to directly assess whether Diap1 dosage modulates the caspase threshold for apoptosis in the pupa notum.

(Remarks on code availability)

Reviewer #2

(Remarks to the Author)
We appreciate and acknowledge the authors' detailed responses, particularly their clarification of the system's limitations and how these may have constrained the measurements they were able to perform. Based on the revised manuscript, we believe the results largely support their conclusions, and we are supportive of the publication of their work.

(Remarks on code availability)

Reviewer #3

(Remarks to the Author)
I co-reviewed this manuscript with one of the reviewers who provided the listed reports. This is part of the Nature Communications initiative to facilitate training in peer review and to provide appropriate recognition for Early Career Researchers who co-review manuscripts.

(Remarks on code availability)

Reviewer #4

(Remarks to the Author)
The authors have provided an extensive revision addressing my main points of concern. The additional experiments/data largely support the core hypothesis, and I anticipate that the field will benefit from the publication of this work.

(Remarks on code availability)

Version 2:

Reviewer comments:

Reviewer #1

(Remarks to the Author)
I would like to thank the authors for the thorough revision of this manuscript. The authors have addressed all my comments and I have no further concerns regarding publication of this work. I think it is beautiful work!

(Remarks on code availability)

Open Access This Peer Review File is licensed under a Creative Commons Attribution 4.0 International License, which permits use, sharing, adaptation, distribution and reproduction in any medium or format, as long as you give appropriate credit to the original author(s) and the source, provide a link to the Creative Commons license, and indicate if changes were made.
In cases where reviewers are anonymous, credit should be given to 'Anonymous Referee' and the source.
The images or other third party material in this Peer Review File are included in the article's Creative Commons license, unless indicated otherwise in a credit line to the material. If material is not included in the article's Creative Commons license and your intended use is not permitted by statutory regulation or exceeds the permitted use, you will need to obtain permission directly from the copyright holder.
To view a copy of this license, visit https://creativecommons.org/licenses/by/4.0/

Point by point answer to reviewers.

Integration of past caspase activity biases cell elimination *in vivo*

General answer:

We would like to thank the reviewers for their constructive comments and suggestions as well as the appreciation of the novelty of our work. While the reviewers globally appreciate the originality of our approaches and the importance of the model we propose, they raised some concerns on some interpretations and made a number of suggestions that we will summarise below:

- They ask for further validation of the accuracy of the GC3Ai sensor
- They propose to explore an alternative memory effect based on DNA damage
- They suggest validating the priming using direct measurement of caspase following a pulse of optoDronc activation (which is not possible with the current tools, see below)
- Further characterization of the TSF machine learning predictions
- Provide more raw data

To answer these points, we provide now the following novel experiments and analysis either in figures for reviewers or in the article:

- Perform a ramp of optoDronc and GC3Ai measurement (article)
- Show GC3Ai levels upon miRNA depletion of all three RHG genes (reviewer figure)
- Characterise the pattern of DNA damage to see if this matches the pattern of caspase priming and/or test the impact of a sublethal pulse of caspase on DNA damage (article)
- Provide more single curves and the raw dataset as well as codes (article and repository)

The code and raw data can be found here:

https://gitlab.pasteur.fr/adavidov/caspase_activity_for_cell_death

The two movies full dataset (so called dataframes) can be found here:

https://drive.pasteur.fr/s/yqS8ZH5pSw6rJ3Q

(password: Cummingetal2026!)

- Measure the impact of two optoDronc pulses separated by 30min or 8h on cell death (article).
- Perform new tests on the TSF prediction, including time shuffling, comparison with logistic regression on single time point and cross validation (reviewer figure)

We would like to stress that many of the experiments provided in this study (combination of optogenetic caspase with live tracking of cells *in vivo* and quantitative readout of cell death) are far from trivial and quite tedious in terms of dissection, optimization of microscopy and downstream quantification. Actually, we are not aware of studies pushing that far the combination of optogenetics, live sensors, and quantitative long term live-imaging for understanding cell fate decision *in vivo*. For instance, the characterization of caspase threshold relies on tedious weeks of curation of segmentation to obtain a full movie (despite the latest deep learning-based segmentation), since a full notum represents close to 2,000,000 objects to segment (10,000 cells x 200 time points). We are basically at the limit of what can be done *in vivo* with the tools currently available.

To try addressing some points raised by the referees, we have also tried to use a novel non-published red caspase sensor that we generated recently (RCS1, based on the complementation of mScarlet3, the brightest red fluorescent protein). This would allow the combination of optoDronc stimulation (blue light) while imaging the dynamics of caspase activity (currently not possible with GC3Ai sensor, which is excited by blue light as well). While our preliminary data suggested that the sensor works and has no clear time delay compared to GC3Ai, we faced difficulties due to the quite low signal-to-noise compared to GC3Ai (e.g.: 3 to 5 fold increase of intensity in dying cells compared to control for GC3Ai, only 15% increase for RCS1) (see **Figure R1** and **R2** below). We rapidly realized that RCS1 was not sensitive enough to detect sublethal caspase activity (comparing the dynamics upon full optoDronc activation, see below). We have documented below our tests with this tool to demonstrate that we tried our utmost to develop and implement a way of combining optogenetic caspase activation with live measurement of caspase dynamics. Further optimization would be required to develop a rapid and more sensitive red caspase sensor, which remains a non-trivial technical challenge and goes far beyond this study.

Yet, we have other ways to address the points raised by referees and we believe we provide further argument/data/quantifications supporting our conclusions. We hope the reviewers will find our answers satisfactory.

Point by point answers

All the answers can be found in blue.

Reviewer #1 (Remarks to the Author):

This work by Levayer and colleagues investigates how cells decide to live or die following caspase activation in the Drosophila pupal notum epithelium. Traditionally, effector caspase activation was considered a point of no return in apoptosis. However, evidence provided in the last 10 years has suggested that sublethal caspase activity is common and may serve non-apoptotic roles. The authors here aim to understand how cells integrate caspase activity over time to make life-or-death decisions.

Using a combination of live imaging and machine learning, the authors performed a quantitative single-cell analysis in a live tissue context, the pupal notum. Using this approach, the authors demonstrate that there is a heterogeneity in caspase thresholds for apoptosis across cells in the pupal notum. Machine learning showed that caspase activity is predictive of apoptosis, but with significant variability, which suggests additional regulatory layers. They then showed that prior sublethal caspase activation lowers the threshold for future apoptosis. Optogenetic induction of caspase activity (optoDronc) confirms that cells that were previously exposed to caspases, are more likely to die upon reactivation. Furthermore, they showed that spatial patterns of apoptosis susceptibility correlate with past caspase activity. Depleting the pro-apoptotic gene hid flattens these spatial patterns (although does not completely erase it), confirming the role of past caspase exposure. Finally, the authors demonstrate that cells with a history of caspase activation are more likely to be eliminated when competing with neighbors. This introduces a non-autonomous, history-dependent mechanism of cell competition, which could influence clonal selection during development and potentially in tumorigenesis.

This study identifies a novel regulatory layer in apoptosis decision-making whereby past sublethal caspase activity primes cells for future elimination. This finding redefines caspase activity as a dynamic and memory-encoded input that contributes to tissue patterning and cell competition. The figures clearly support the major claims. I think these findings have important implications for our understanding of apoptosis regulation in developmental biology, cell competition and disease.

While this work is very compelling, I still have a few comments, concerns and questions.

1. A major weakness of the work is that the molecular mechanism underlying the memory of past caspase activity remains unidentified. I recognize that the authors have examined some possibilities, without success. I hope it is a long-term goal of the authors to address this question.

Answer: We totally agree that this is an essential question, but as highlighted in our discussion we failed so far to find a clear mechanism, mostly relying on the fact that this priming is probably multifactorial and is unlikely to boil down to a single factor. We are currently starting a more thorough screen to identify factors modulating the sensitivity to caspases, but this is a complicated screen involving optogenetics that will take several years. We hope the reviewer will understand our perspective on this.

2. Some conclusions rely on correlation (e.g., high GC3Ai signal correlates with future apoptosis) rather than direct causation. It is desirable to directly test causality of caspase priming. Use optoDronc to deliver a sublethal caspase pulse, then block caspase activity genetically or pharmacologically and apply a second pulse. This

experiment may also determine whether the priming effect is reversible and whether it requires sustained caspase activity or downstream signaling.

Answer: This is an interesting suggestion which mostly aims at testing the relative contribution of initiator and effector caspases. However, there is currently no experimental way to address the point of the reviewer. What they suggest would require the capacity to block transiently and with very acute time control effector caspase in less than 1 hour. There is virtually no way to do this genetically, nor with a drug (we can inject in the notum but cannot do wash out and in our hands drug injections require hours to be effective in the notum). In principle, this could be done with some optogenetic tool (that would still need to be designed), but even if we managed to develop an optogenetic tool to inhibit effector caspases, all the available optogenetic systems that are genetically encoded rely on blue light stimulation, like optoDronc, which means that we would not be able to separate the timing of induction of optoDronc and the timing of induction of the effector caspase inhibitor.

Of note, we already previously showed that the combination of optoDronc activation with overexpression of p35 (an effector caspase inhibitor, expressed since 48h) is sufficient to prevent extrusion (see Valon et al Dev Cell 2021 and Villars et al Nat Comm 2022). We are therefore confident that effector caspase activity is required for the engagement in apoptosis.

Finally, we would like to stress that we already outlined the reversibility of the priming as we could hardly see an increase of death following a second pulse of optoDronc activation when cells were exposed to a first pulse more than 5 hours before (**Fig. 2D,E**). We complement this with even longer waiting time (8 hours) to show that there is definitely no impact beyond this time point (see answer to referee 4).

As for the perdurance of caspase activation following a pulse of optoDronc, we now provide estimation on former data and novel data suggesting that effector caspase must remain active for no more than 30 minutes (see below).

3. How long is optoDronc stable and active after the pulse?

Answer: This is a very relevant point. We don't have a proper tool for fast and accurate measurement of the pool of active Dronc live. There is a live sensor which is based on the relocalisation of a fluorophore from the membrane to the nucleus upon cleavage by active Dronc (Baena-Lopez et al. Development 2018), however bear in mind that to our knowledge the precise kinetics/delay of this sensor was never characterized (for instance dynamics will also be influence by import/export rate in the nucleus, as well as degradation rate). Moreover, the only available tool is based on a Histone tagged with GFP, which prevents us from doing time lapse combined with optoDronc (the imaging will fully activate optoDronc and kill all the cells in 15 min). Note that we cannot use GC3Ai for a similar reason, as we need to get the intensity variation over time to

calculate the derivative (the proxy for caspase activity), which requires sustained blue light imaging and will therefore fully activate optoDronc.

We have however several indirect ways to estimate the duration of caspase activity following a pulse of Dronc activation. First, it was previously suggested that active caspase are relatively short lived (see Albeck et al., Mol Cell 2008, Tawa et al. Cell Death Diff. 2004, Suzuki et al PNAS 2001, Choi et al, J Biol Chem 2009). Accordingly we previously found that a well-defined pulse of ERK activity could inhibit caspase within 15 min, which is only compatible with fast turnover (below 15min) of active caspases (see Valon et al Dev Cell 2021, Figure 4C). Along the same line, we showed here that a pulse of optoDronc will increase the rate of cell death for roughly 50min before going back to steady state rate (see **Fig. S3E**). Considering that extrusion takes on average 20 to 30 min (Villars et al. Nat. comm. 2021), this suggests that Dronc activity is unlikely to perdure at significant levels beyond 30 min after activation. While this does not exclude persistent low/sublethal activity level, it suggests that the vast majority of active Dronc disappears in less than 30 min.

Finally, to complement these observations, we have tried to use a new red caspase sensor (RCS1) based on the same principle as GC3Ai starting from the structure of mScarlet3 (**Figure R1A,** one of the brightest and most stable red fluorophores). This tool was never published before. The sensor reports caspase activity with the same kinetics as GC3Ai (see **Figure R1A-D)** but with much lower signal to noise (5 to 10 fold increase of intensity in dying cells for GC3Ai, 15-20% increase for RCS1). We tried to use this tool to assess the perdurance of caspase activity. Unfortunately, the tool was not sensitive enough to detect sublethal caspase levels. This is exemplified by the comparison of caspase kinetics upon full optoDronc activation. While GC3Ai shows a clear and rapid global increase after 15 minutes (see **Figure S3F,G** and **Figure R2A** below), we could not detect any clear increase of intensity of RCS1 except in patches of rapidly dying cells (**Figure R2B**). We tried our best, but currently lack sensitive and fast caspase sensors allowing the precise measurement of the point raised by the referee. We now mention in the main text the estimation of caspase activation perdurance in the discussion line 401:

*"This memory is unlikely to be explained by the perdurance of active caspases which were previously shown to be very short-lived[1-4], in agreement with our previous measurement of the short time delay between pulses of ERK and inflection of caspase activity (averaged delay of 15min[5]) which is not compatible with long-lived activated caspases. Furthermore, the transient increase in extrusions following a pulse of optoDronc (returning to physiological levels after 50min, **Fig. S3E**, with extrusion lasting on average 20 minutes) is compatible with relatively short-lived (<30min) active caspases."*

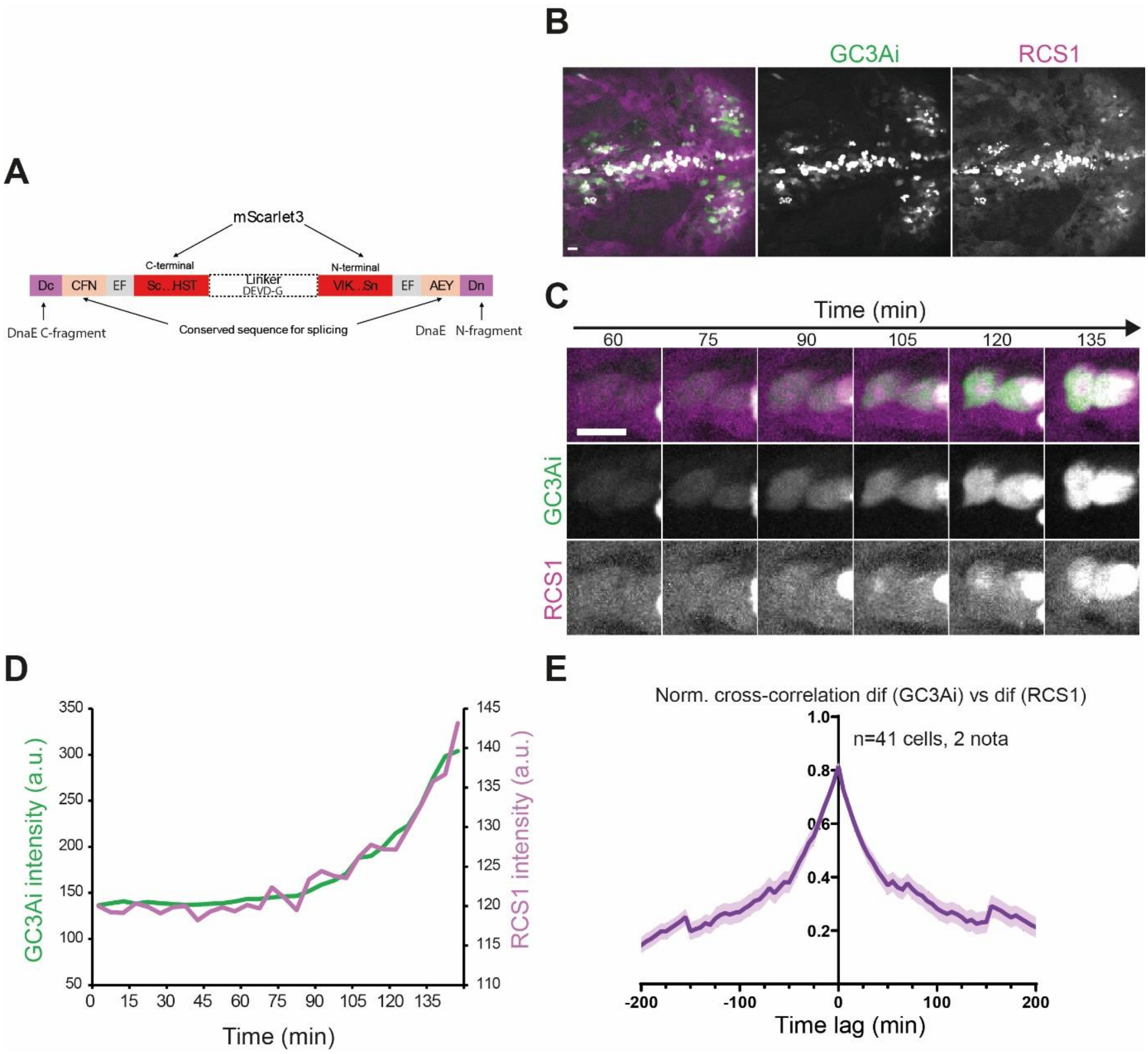

Figure R1: A novel red caspase sensor: RCS1.

A: Design of the construct based on [6] using mScarlet3, DnaE C and N fragments that will be used upon protein folding, and by linking the native Nter and Cter with a DEVD cleavage site. The construct was first tested in S2 cells and optimized by removing first amino-acid from Nter and Cter, and tested for activation upon apoptosis induction. **B:** Pattern of UAS-RCS1 signal expressed in the pupal notum (magenta) driven by the pnr-Gal4 driver and compared to UAS-GC3Ai. Note the similarity of the pattern, but also the higher baseline in the lateral domains for RCS1. Scale bar is 10μm. **C:** Snapshot of a single cell dying showing GC3Ai and RCS1 signal. **D:** Single curve of GC3Ai and RCS1 raw intensity from the example shown in C. Note that while the dynamics are very similar, the intensity range of variation is very different (from 150 to 300 for GC3Ai, 120 to 140 for RCS1, illustrating the low signal to noise and dynamic range). **E:** Averaged normalized cross-correlation between GC3Ai and RCS1 from 41 dying cells. The curves are centered on 0, suggesting there is no significant lag in global dynamics between GC3Ai and RCS1.

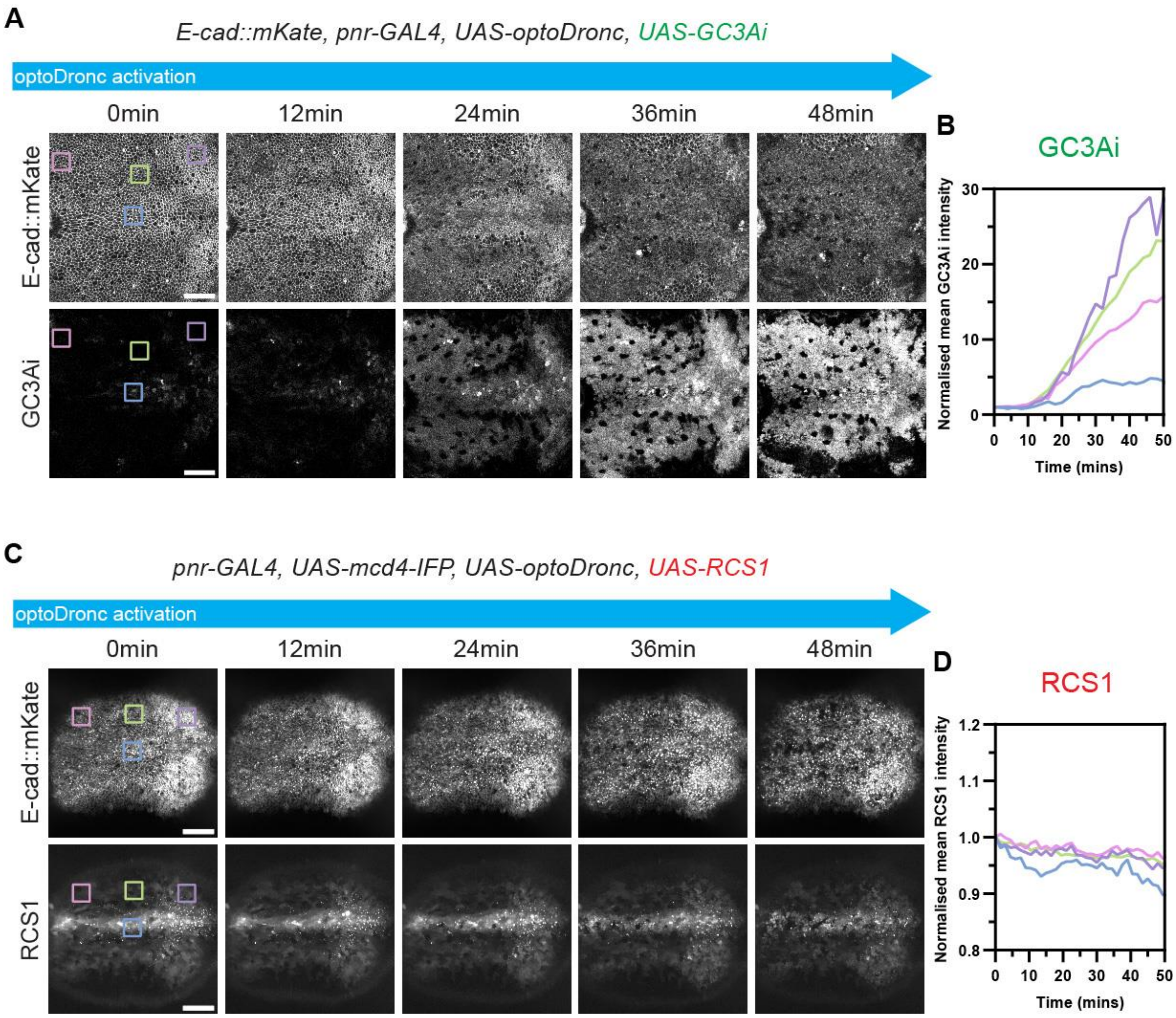

Figure R2: RCS1 is not sensitive enough to detect sublethal caspase activation.

A: Snapshots of a pupae expressing GC3Ai (bottom), E-cad::mKate (top) and optoDronc in the pnr domain upon full and constant activation of optoDronc (data from **Fig. S3F,G**). Scale bars 50μm. The coloured squares show the ROI used for GC3Ai intensity measurement shown in **B. C:** Snapshots of a pupae expressing RCS1 (bottom), E-cad::mKate (top) and optoDronc in the pnr domain upon full and constant activation of optoDronc. Scale bars 50μm. The coloured squares show the ROI used for RCS1 intensity measurement shown in **D.** Scale bars 50μm.

4. GC3Ai may not perfectly reflect real-time caspase activity due to its accumulation and stability, which may over-represent low, transient caspase activity and potentially mask fast dynamics. In fact, GC3Ai's cumulative nature may obscure the precise timing of caspase activation. A sensor with faster turnover or cleavage specificity might improve temporal resolution. If available, use of a faster-turnover caspase sensor might be better suited to capture rapid fluctuations of caspase activity and thus may better define the duration and decay of the caspase "memory."

Answer: Thank you for giving us a chance to clarify this point. It is indeed a legitimate concern, however that is the very reason that we use the derivative of the GC3Ai intensity, which is a good proxy for the instantaneous effector caspase activity. We showed previously that this could detect transient pulses of caspase activity (Valon et al Dev Cell 2021). In fact, we could even correlate the downregulation of caspase activity with pulses of ERK activity using the derivative of GC3Ai intensity (with a lag time of 15 min), which exemplified the relevance of GC3Ai intensity derivative to measure instantaneous caspase activity *in vivo* and during physiological variations.

To our knowledge, there is no live sensor available with a faster turnover. In our hands, GC3Ai is the most sensitive and fastest (in the sense of delay between caspase activation and detection of a signal) live sensor of effector caspase available. Others are based on FRET (so more difficult to handle in terms of signal to noise), or transcription readout or relocalisation of a fluorescent probe from membrane to the nucleus where kinetics will also be influenced by import/export rate and poorly quantitative.

Last but not least, engineering the GC3Ai sensor to add a PEST sequence would be very challenging. The reviewer can check the original report of the tool: https://www.nature.com/articles/ncomms3157. This is based on modulation of the initial N and Cter of GFP where a loop containing the DEVD sequence is added to prevent proper protein folding, and where there is very fine tuning to optimize signal to noise (the ratio of intensity between caspase-cleaved and non-cleaved sensor). Adding or removing as little as one amino acid here is enough to change the signal to noise by 80%. There is no quick and easy way to change these probes (in fact we've been trying to generate a better red sensor for the last 3 years and only reached something acceptable now).

5. Along those lines, is there a caspase sensor with reversible activation kinetics to differentiate between transient and sustained caspase signaling which could be used here?

Answer: As mentioned above, this tool does not exist, but we believe the derivative of the GC3Ai intensity matches very well this expectation and was proven to be able to capture physiological reversion of caspase activity (see Valon et al. 2021 Dev Cell).

6. The manuscript would benefit from a clear schematic model summarizing the proposed model of how past and current caspase activities are integrated to determine cell fate

Answer: This is a very good suggestion. We have now added a schematic explaining how past and current activity can be combined to predict cell decision (while it should be clear that we do not have a clear molecular view of how this works), see new **Fig. 5A**.

7. Do caspase-primed cells exhibit changes in mechanical properties (e.g., tension, stiffness) that might influence extrusion?

Answer: This was indeed one of our favourite hypotheses. However we do not see obvious changes of MyoII (the main regulator of tension), or microtubules upon mild caspase activation (see **Figure S5**). In fact, we did not notice either any clear change of cell shape, suggesting that if anything, the change of mechanical properties must be very minor (otherwise cell shape would necessarily be affected). We have clarified this point in the discussion line line 422:

*"Sublethal caspase activity can also modulate cytoskeleton organisation[7], hence modulating the probability to enter extrusion. Accordingly, sublethal Dronc activity was shown to downregulate MyoII in the pupal notum[8], and global tension reduction was recently shown to be permissive for cell extrusion[9]. However, we could not detect clear modulation of MyoII intensity following sublethal pulses of optoDronc (**Fig. S5E, movie S13**). We also did not notice any clear change of cell shape in the zone exposed to sublethal caspases suggesting that cell mechanics is unlikely to be significantly altered. "*

8. Does apoptotic memory affect epithelial repair, regeneration, or barrier function?

Answer: Thanks for these suggestions. There are already previous works demonstrating that sublethal caspase activation promotes tissue repair and wound healing (see for instance Fujisawa *et al.* Development 2019 Miura lab). While these are interesting questions, we believe they really go beyond the scope of this article which is trying to understand the process of cell death engagement and not exploring non-apoptotic functions of caspases. We hope the reviewer will understand our point of view.

9. In the experiment in Figure 2D, the authors exposed the lateral domain of the midline to optoDronc twice and the contralateral region as a control. Could the authors perform the reverse experiment - exposing the contralateral region to optoDronc twice while using the lateral domain of the midline as a control - to rule out potential position effects?

Answer: Thanks for this suggestion. In fact, the very reason why we use the contralateral region is that we do not expect to see any asymmetry between left and right heminota. Previously, exhaustive quantitative characterizations have shown that the two heminota behave exactly the same up to few cell diameter precision in term of cell shape, intercalation, cell division and cell death (see for instance Guirao *et al.* elife 2015, https://doi.org/10.7554/eLife.08519) which we also confirmed in our previous quantitative characterization of cell death (Valon *et al.* Dev Cell 2021). To clarify this we include this data below (**Fig. R3A,B**). Essentially, in the absence of perturbation the two sides of the nota are perfectly mirroring one another.

Finally, our mapping of cell death following optoDronc mild activation always displays very clear symmetry in the supernumerary death distribution (**Fig. 1G**, **3A,B**, and see below showing single notum, **Fig. R3C**), suggesting that bilateral symmetry is very strong in the pupal notum when it comes to cell death regulation (and as far as we know all other morphogenetic features). We are therefore very confident that the two heminota should behave the same. We have included this argument now in the article line 216:

*"Note that the heminota should normally be perfectly symmetric in terms of cell death, as demonstrated by the highly symmetric pattern of cell death in a physiological context[5, 10], and the symmetric pattern of additional death triggered by a global pulse of Dronc activation (**Fig. 1.G, 3A,B**)."*

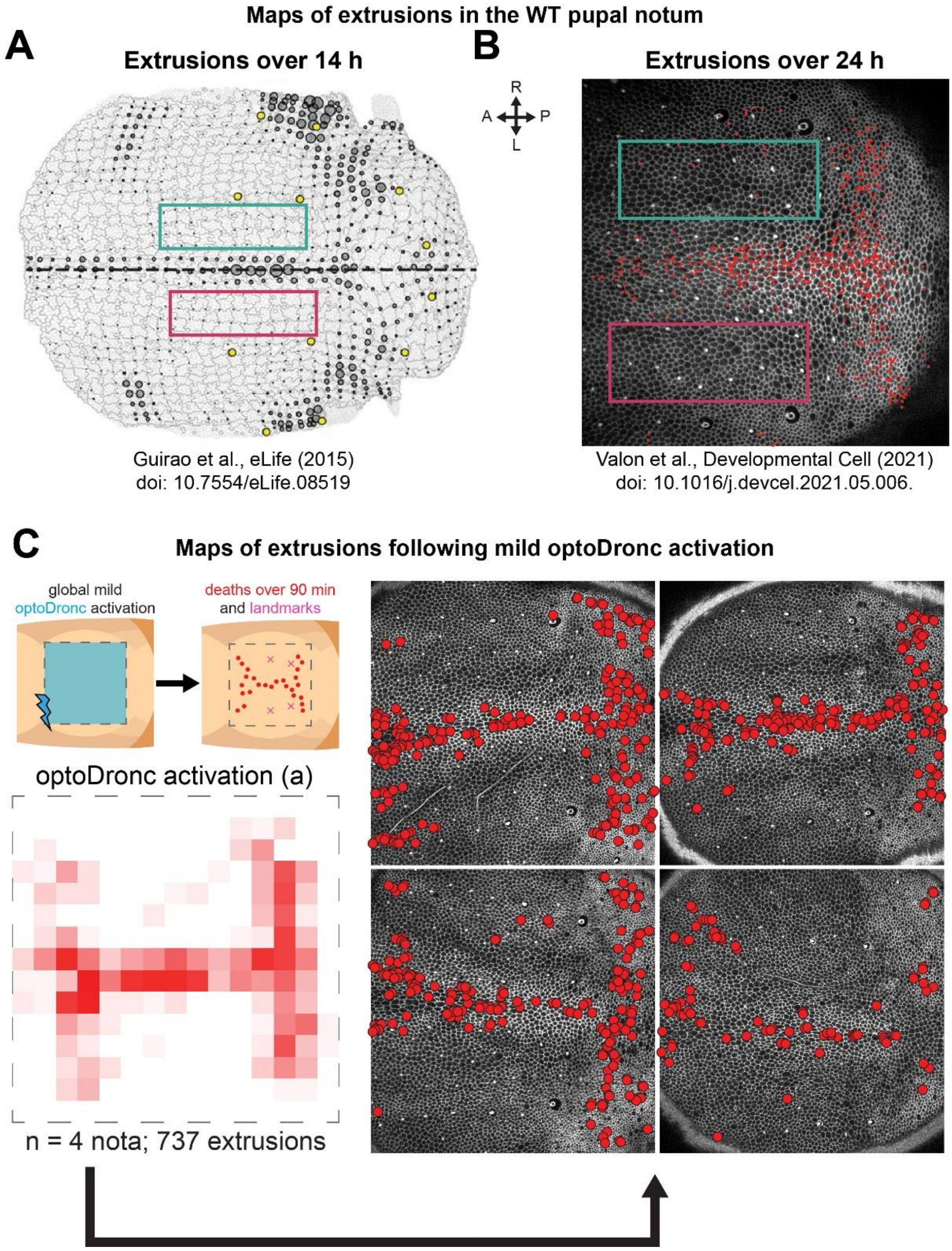

Figure R3: Cell death bilateral symmetry in the pupal notum. A: Extract from previously published results[10] showing the symmetry of cell death distribution in unperturbed pupae over 14h. The area of grey circle is proportional to the local number of cell death. Dotted line is the midline. Cyan and red rectangles show the approximate position of the regions where we perform the pulse of optoDronc activation separated in time for the memory experiment. **B:** Distribution of cell death in a single pupa over 24h (red dots) from [5], note that the scale is different from **A**. **C:** Distribution of supernumerary cell deaths triggered by global activation of optoDronc (from experiment in **Fig. 1F,G**). The heat map shows the averaged number of deaths on 4 nota, the right picture shows the actual distribution of death (red dots) on each notum. Note that the pattern is overall very symmetric in left and right (check for instance in the posterior domain).

10. Although hid is the primary apoptosis-inducing gene in the pupal notum, hid RNAi fails to completely eliminate caspase activity. This failure might simply reflect inefficient knockdown of hid. Alternatively, reaper and grim may also be (minor) contributors to apoptosis induction. To test this, the authors could simultaneously target all three genes using an available RNAi line that targets hid, reaper, and grim together, then assess whether this produces a stronger effect on caspase activity.

Answer: This is a good suggestion. In fact, we previously compared the efficiency of hid-RNAi and the micro RNA targeting hid grim and reaper in the wing disc. We actually found that hid-RNAi was more efficient at inhibiting cell death, which could be explained by the dominant role of hid in the tissue and the more efficient depletion of hid mRNA with the RNAi line compared to microRNA (which we confirmed by QPCR, see Matamoro-Vidal *et al.* Curr Biol 2024). We have already tried to use the microRNA in the notum and still noticed a few cells with active caspase in the midline, just like hid-RNAi (**see Figure R4 below**). It seems that hid-RNAi is working as efficiently as miRHG to block apoptosis upstream of Dronc in the notum. It is actually quite difficult to block every cell extrusion, and we already showed previously that even p35 overexpression will "only" reduce by 95% the rate of cell extrusion (we never completely block it). We would like to stress though that despite the few sporadic cells that still die, the spatial pattern of non-lethal GC3Ai signal seems completely abolished in hid RNAi background, which makes it a good background to "erase" most of the pattern coming from previous exposure to caspase.

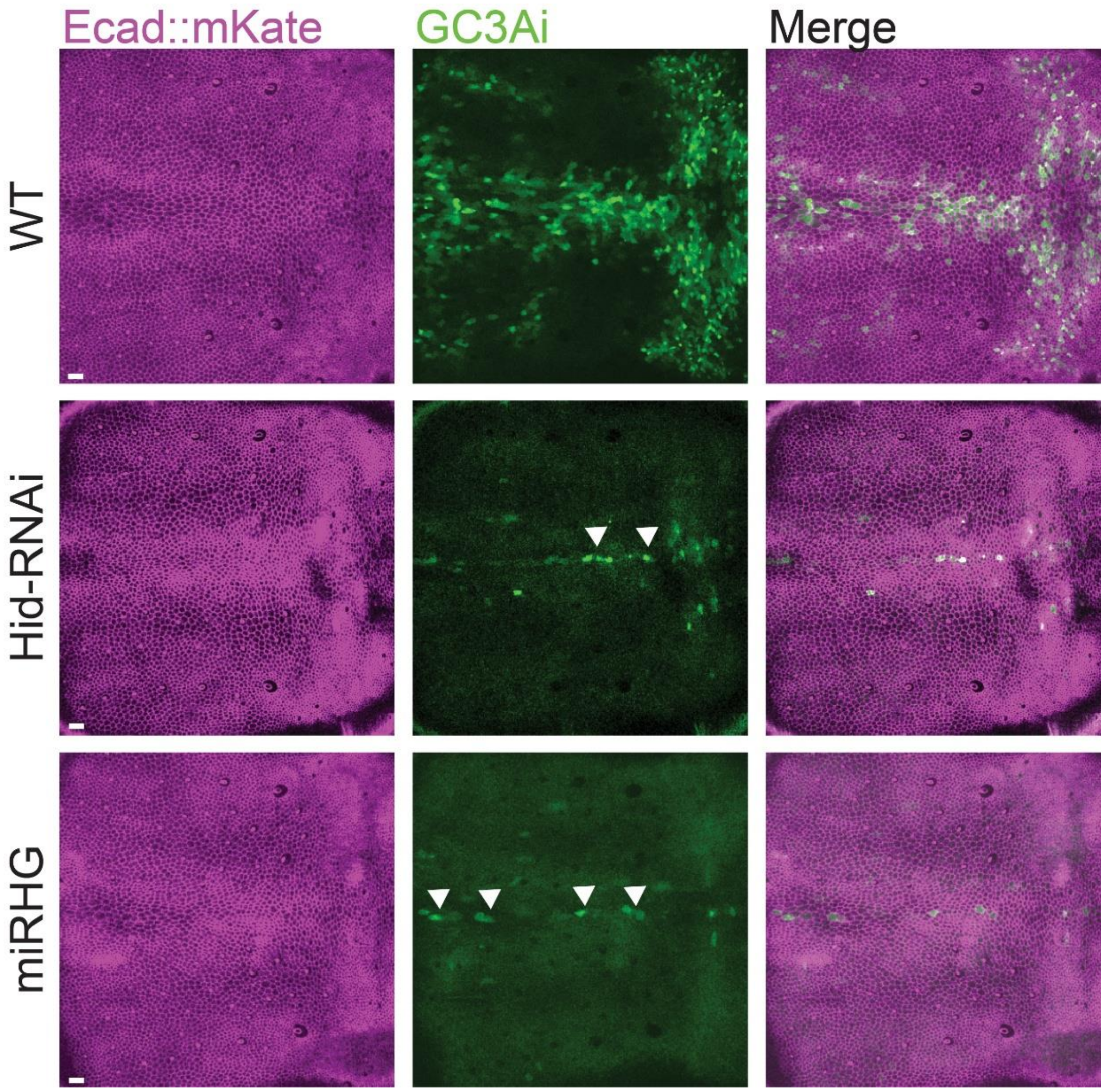

Figure R4: GC3Ai signal in control, hid RNAi and miRHG. Nota expressing hid-RNAi or miRHG in the pnr domain as well as GC3Ai (green), and E-cad::mKate (magenta). White arrowheads show the few sporadic cells still activating caspases in the hid-RNAI and miRHG backgrounds. Scale bars=10µm.

11. In the Discussion, lines 360-365: Shlevkov and Morata (2012) discussed a positive feedback loop in dying cells. Maybe the authors can follow up on their work and further examine this loop.

Answer: Thanks for pointing this very relevant study. Indeed, this type of positive feedback could be very relevant for the memory. This was also one of the key motivations to test the role of JNK in the memory. As pointed out in former Figure S4 (now **S5**) however, we could not find any match between the pattern of caspase activation (especially in posterior region) and JNK activity despite hours of exposure to sublethal caspases. This is therefore unlikely to be involved in this process. We now discuss this paper in the discussion and the relevance of this positive feedback when discussing the results of **Fig. S5** line 408:

"For instance, JNK pathway activity can be tuned by caspase[11] and directly impact apoptosis engagement which can generate a positive feedback loop in dying cells[12].

*However, while a fluorescent reporter of JNK (TRE-RFP[13]) shows activation in the midline, there was absolutely no sign of activation in the posterior domain which is exposed to caspase activity for several hours during development of the notum (**Fig. S5A**, **movie S10**).”*

12. The authors frequently use the phrase "for the first time," which is problematic for several reasons. First, most scientific findings are by definition novel, making this phrase redundant. Second, it's difficult to definitively claim “for the first time” given the vast body of existing literature. For instance, while the authors claim to have demonstrated heterogeneity in caspase thresholds for apoptosis across cells for the first time (Figure 1), Fan and Bergmann (2014) made similar observations in the eye imaginal disc, and M. Miura's group made comparable findings in Koto et al (2009). The authors should moderate their language and acknowledge these prior studies in their discussion.

Answer: This is indeed relevant. We have removed those statements and mention these two studies. It is true that tissue heterogeneity was already demonstrated, but we provide here a single cell view of this heterogeneity as well as its temporal dynamics which we believe is completely novel (as it needs single cell tracking and temporal dynamics). We mention these papers in the introduction line 70:

“Previous works in the Drosophila larval eye disc[14] and in the pupal notum[15] highlighted the role of modulation of the protein levels of Diap1, an inhibitor of apoptosis, which can tune cell sensitivity to apoptosis.”

13. Along these lines, both Fan and Bergmann and Koto et al. identified varying Diap1 protein levels as the basis for differential cellular sensitivity to apoptotic signals. Looking at Suppl fig S4H, there is variation in Diap1 levels. Interestingly, reduced Diap1 expression is reduced in regions of high apoptosis within the pupal notum. This could account for the heterogeneous cellular responses to uniform caspase input (optoDronc) and may also explain the effects of past caspase activity. The authors should investigate this further by experimentally manipulating Diap1 levels.

Answer: This is a very relevant point and the reason why we indeed already checked the status of Diap1 in the former Figure S4 (now **S6**). First, while there is indeed some reduction of Diap1 in a narrow band in the midline and in part of the posterior region, these regions do not really match very well the extent of the regions sensitive to caspases and showing death upon mild optoDronc activation (compare **S6A**, with **Fig 3A,B**) which makes Diap1 unlikely to explain the full pattern. We now performed spatial correlation analysis between Diap1 protein levels and optoDronc induced extrusions pattern in early and late pupae, outlining relatively low correlation (see Figure **S6C-F**). Secondly, when we exposed cells to transient caspase activation (targeting a full heminota) we could not see any clear reduction of Diap1. Of course, Diap1 could still play a role here, especially if this is not mediated by a modulation of the total protein

pool of Diap1 and rather involves for example some posttranslational modifications. We now clearly mention that in the discussion line 438:

*"We observed a narrow band in the midline with low Diap1, as previously reported using a reporter of Diap1 degradation[15], and a decrease in Diap1 levels at the posterior edge of the tissue at late stage of development (**Fig. S6A**, **movie S15**). Yet, a more detailed spatial correlation quantification revealed a poor match between the pattern of cell death triggered by optoDronc in early and late pupae and the corresponding pattern of Diap1 (**Fig. S6C-F**). Moreover, we saw no change in Diap1 levels following a sublethal pulse of optoDronc (**Fig. S6B, movie S16**). Altogether, this suggests that the pattern of Diap1 protein level is unlikely to explain the priming. However, we cannot exclude the existence of more subtle effects based on posttranslational modification of Diap1 protein or modulation of its interactors[16]."*

It is also true that we didn't test whether modulation of Diap1 pattern could affect the spatial pattern of caspase sensitivity. This would be hard to interpret though as Diap1 overexpression or downregulation will probably also affect the physiological pattern of caspase activity (like hid RNAi). If we observe a change of death pattern upon mild optoDronc activation, this could be equally explained by the change of cell history/past caspase exposure, or a direct impact of Diap1. We are therefore not sure this experiment will really be informative. To clarify this point, we now provide a more detailed comparison of cell death pattern and Diap1 pattern in new **Figure S6** and mentioned the potential role of Diap1 especially if based on posttranslational modifications.

Reviewer #2 (Remarks to the Author):

In this manuscript, Cumming at al. investigate whether past sublethal caspase activity can influence the likelihood of a cell to undergo apoptosis in the Drosophila pupal notum epithelium. Through live imaging and machine learning, they reveal large variability in caspase sensitivity across cells, and identify past caspase activation as a correlated factor potentially contributing to this heterogeneity. Optogenetic activation of caspases increased cell extrusion events, supporting the role of past caspase activation in determining cell extrusion.

While the manuscript presents an interesting hypothesis regarding the role of past caspase activity in priming cells for apoptosis, the major claims are not sufficiently supported by the data presented. Specifically, two major claims: 1) that caspase thresholds vary across cells, and 2) that cells integrate past caspase activity to modulate sensitivity to apoptosis, are neither clearly defined nor experimentally validated. Several conclusions rely on correlative observations without demonstrating causality. Moreover, the study does not distinguish its proposed model from simpler alternative explanations (see Point #3). In its current form, the manuscript lacks necessary evidence and mechanistic insights needed to support its major claims. For these reasons, we cannot recommend the manuscript for publication.

Answer: We understand the concern of the reviewer. Demonstrating that caspase activity varies across cells and that past exposure primes cells for apoptosis are indeed the two major conclusions of this article. We however kindly disagree with the reviewer when stating that these claims are not supported by our data and just based on correlative observations. We will give further explanations and details below. While indeed the first intuitions were based on correlative data, the past priming is supported by several direct experimental perturbations including:

1) the behaviour of cells exposed to two separated pulses of caspases

2) the demonstration that the spatial pattern of previous caspase activity spatially biases death upon optogenetic induction of caspases, which is erased by hid-RNAi.

3) the correlation between past caspase activity and probability to die following a pulse of caspase at the single cell level

4) the higher susceptibility of clonal death during normal development when cells were previously exposed to a pulse of caspase activity

All these observations can only be explained if past exposure to caspase activity primes cells for apoptosis.

Regarding point #3 raised by the reviewer, we actually believe that the hypothesis proposed by the reviewer totally matches our model and was exactly the sense of what we explored in the discussion of the article. Whether the priming is driven by some substrate cleavage, DNA damage, or any sort of molecular encoding, this matches with the notion that sublethal caspase activation can prime cells for apoptosis. The disagreement here might be purely semantic and we wonder if the terminology “integrate” that we used in the title might have been misunderstood. It is true that in essence, the notion of integration (in its pure mathematical definition) does not encapsulate the notion of “erasing” after a certain time. In that sense, the terminology “short term memory” might be more appropriate and more neutral regarding how cells “compute” the phenomenon and we therefore decided to change this in the title and abstract. We hope this will be judged as more appropriate by the reviewer.

1. The authors are encouraged to improve their data presentation. The manuscript presents processed metrics (e.g., correlation and probabilities) without showing the accompanying raw data, which limits reader’s ability to independently evaluate the validity of the claims.

Answer: We agree that showing raw data is always more compelling, but we would like to remind the reviewer that our analysis is based on a very large dataset that represents over 15,000 cells tracked over nearly 200 time points from which we quantify many individual parameters. We obviously cannot provide every single curve in the figures, and the metrics based on correlation and probabilities remain the most appropriate ones which provide an intuition of the behaviours. We can certainly provide

a higher number of individual examples and provide to the reviewer the dataset to reassure them that all these averaged curves are based on a large and solid dataset. We now provide a repository link containing the raw dataset and codes that the reviewer can explore. We also now provide several randomly picked single cell examples of dying and non-dying cells in the new **Figure S2** (note that the scale of the y-axis is different for each curve).

The code can be found here:

https://gitlab.pasteur.fr/adavidov/caspase_activity_for_cell_death

The two movies full dataset (so called "dataframes") can be found here:

https://drive.pasteur.fr/s/yqS8ZH5pSw6rJ3Q

(password: Cummingetal2026!)

1.1 To support the claim of heterogeneous caspase thresholds, the quantification of area, mean GC3Ai intensity, and caspase activity (as shown in the lower panel of Fig. 1C) should be provided for multiple cells. Ideally, this analysis should include all the cells quantified to generate Figures 1D & 1E to convincingly demonstrate heterogeneity.

Answer: As highlighted above, we cannot provide the 3 times 15,568 curves (area, GC3Ai raw and derivative) to support this claim. We now provide more single curve examples in **Figure S2** and the dataset to the reviewer (see above).

1.2 In Figure 2C, the accompanying time-series data of various morphometric and caspase-related parameters need to be shown to clarify the basis of the reported correlations.

Answer: Figure 2C is based once again on 1,130 dying cells and cloud plots for every time bin of each parameter. We are not sure to see how to provide in a readable way each cloud plot (more than 180 plots), nor the individual cell curves for all these parameters. **Figure 2B** represents the type of data obtained for each bin and illustrates well the analysis process. To clarify the process, we provide now in **Figure S4** single curves of all the individual parameters and decompose the process leading to one correlation value. We hope this will help.

1.3 The study only reports standard error to indicate confidence in the results. The standard error is expected to be small due to the large number of cells. To demonstrate and support caspase heterogeneity, the authors should report the standard deviation instead, or in addition to standard error.

Answer: Standard error is relevant when one wants to illustrate the statistical relevance of a claim. **Figure 1D,E** already gives a sense of the standard deviation by

representing the "cumulative" probability to enter apoptosis which is visible through the sharpness of the curve. It is more relevant in this case to use error bars to represent the statistical strength of each point and the confidence interval (which gets larger for extreme values which are less represented in the dataset). We now provide in the supplementary figures the distribution of caspase thresholds and the number of observations for each category (new **Fig. S2C**). In **2E**, s.e.m. is meaningful as we want to visualize the significance of the differences, one just need to multiply by the square root of 5 (or 7) to get the standard deviation (we are happy to provide it if the reviewer thinks this is more appropriate but the purpose here is to define significance so we would prefer to stick to s.e.m.). **Figure 3E** represents the confidence interval calculated on proportion (for each point, we represent the proportion of dying cells for a category of raw GC3Ai intensity), we cannot calculate here a standard deviation. Same for **Figure 4D** which represents a proportion of dying cells out of all the cells in the clones, there is no standard deviation here.

2. The central claim of the study that prior induction of caspase activity lowers the caspase threshold for cell extrusion and prime cells for apoptosis (lines 199-200) is not adequately support by data:

2.1 Caspase thresholds were not defined or quantified in the results.

Answer: With all our respect, we don't understand this claim. We quantified caspase thresholds on 1,130 dying cells. This is indirectly shown in **Fig. 1E** which shows the cumulative probability to enter in apoptosis above a certain caspase activity value. We also defined in the text how these thresholds were measured: detection of inflection point of cell area to detect the onset of extrusion (the point of no return) and measurement of the derivative of the GC3Ai signal at this time point. Maybe this was not clear enough in the text and we apologise for that. To have a more "direct" readout of the caspase threshold distribution, we now provide the distribution of the caspase threshold for all dying cells in **Figure S2C** (which is more or less equivalent to the derivative of the curve shown in **1C**). Note that the peak observed close to 0 is mostly due to the large overrepresentation of this category throughout the cell population (in fact the probability to die for these values is close to 0, check the number of observations for each category in the small green inset in **S2C**).

This is now mentioned in the text line 148:

"Here, we define 'caspase threshold' as the caspase activity value at which cells engage in cell extrusion."

And line 157:

"While this result suggests that effector caspase activity is partially predictive of cell death engagement, it also outlines the significant cell-to-cell variability in the caspase threshold associated with commitment to apoptosis/extrusion, as the range of caspase

*activity levels at which cells engage in apoptosis spans nearly two orders of magnitude (**Fig. 1E,** see **Fig. S2C** for the distribution of caspase activity values at which cells engage in extrusion).*

2.2 The experiments in Figure 2D and Figure 3 do not establish a causal link between past GC3Ai levels and subsequent cell extrusion. To demonstrate causality, GC3Ai levels need to be monitored in real-time from optoDronc activation until cell extrusion. Importantly, applying different levels of optoDronc activation can test whether modulating GC3Ai levels can change the caspase threshold, if there is indeed a causal relationship.

Answer: We totally agree that this will be the ideal way to measure how optoDronc modulates caspase threshold. There is however here an important experimental limitation: optoDronc is activated by blue light (even very mild intensity) and the imaging of GC3Ai is based on blue light exposure. It is therefore impossible to perform sublethal caspase activation followed by live imaging of GC3Ai (which would just kill all the cells, see figure **S3F**). Of note, GC3Ai is currently the most sensitive live sensor of effector caspase which exists.

We have been trying to develop a red caspase sensor for the last 3 years with many failures (RC3Ai, which is based on mCherry folding, does not work in flies, Schott *et al.* Development 2017). We recently obtained one based on mScarlet3 (one of the brightest red fluorophores) which seems to work with no significant lagtime compared to GC3Ai (see **Fig. R1** above, answer to referee 1), however the signal to noise is 5 to 10 fold less than GC3Ai and we failed to detect sublethal caspase activation (see **Fig. R2** above). We basically reached the limit of what is possible technically. Developing and optimizing a fast and sensitive red sensor remains a tedious and long task that could still take some years and clearly falls beyond the frame of this manuscript.

Of note, even if we would have a good red caspase sensor, it would still be very hard to assess the modulation of death-inducing activity threshold at the single cell level. Indeed, calculating the threshold is based on very tedious segmentation, curation and tracking of thousands of cells. In the experiment combining optoDronc and a red caspase sensor, we cannot use the proper cell contour marker based on E-cadherin for segmentation (only existing in red and green channels). We do have a cytoplasmic membrane marker in far red, but this is full of intracellular bodies and vesicles which make large scale segmentation impossible with reasonable manpower. Here we once more reach the limit of what we can do with the system in terms of multiplexed live imaging + optogenetics *in vivo*. We hope the reviewer will understand these technical difficulties.

Despite this limitation, we still believe that we already provide evidence for the impact of variable previous exposure to caspases on later apoptosis induction, as requested by the reviewer. This is exactly what we show when measuring the cell to cell variation of GC3Ai levels at time 0 (exploiting physiological variation of past exposure) and

showing correlation with cell death following a pulse of optoDronc (**Fig. 3D,E**), and when we demonstrate that sublethal activation of caspase in clones primes them for later death when exposed to later physiological caspase activation (**Fig. 4D**).

3. The study does not distinguish its proposed model, where past caspase activity is integrated to modulate cell extrusion caspase threshold, from a simpler alternative model where initial sublethal activation induces a persistent state of unresolved cellular damage or stress, which sensitizes cells to subsequent apoptosis. Prior studies have shown that sublethal caspase activity can lead to altered cell fates and phenotypes. The current data do not differentiate between regulated past caspase integration and persistent cellular stress or damage. These two models can be distinguished by monitoring GC3Ai levels in real-time after optoDronc activation and measurements of markers of cellular stress or damage (e.g., γH2AX) in the several hours preceding cell extrusion.

Answer: Thanks for giving us a chance to clarify this point. In fact, we are not sure to understand where the contradiction is between what is proposed by the reviewer and what we very openly discussed in the discussion of the article. Irrespective of the molecular nature of the signal responsible for the priming (cleavage of substrate, DNA damage, stress pathway activation…), down the line this is still compatible with the notion that past caspase exposure primes cells for apoptosis. When the reviewer states *"where initial sublethal activation induces a persistent state of unresolved cellular damage or stress, which sensitizes cells to subsequent apoptosis"*, we are not sure to see how this is different from what we propose, unless the reviewer suggests a priming independently of a modulation of caspase sensitivity. We have strong evidence that the "sensitizing" corresponds to a very downstream response since we see more sensitivity to optoDronc activation and subsequent caspase activation, so the priming must act downstream of initiator caspases. We are also happy to change the title and remove the notion of "integration" if that is one source of debate. We propose to use "short-term memory" which would be more accurate to describe a process that perdures for a finite timescale and would be more neutral regarding how the cells "compute" past exposure.

There is also an important difference between what we see and what the reviewer proposes in the time scale. While we do see that the memory is likely to act on the timescale of a few hours, what the reviewer proposes (DNA damage and a persistent state of stress) would probably act on a much longer timescale and does not really seem compatible with our observations confirming priming for few hours. We already tested whether the JNK pathway could explain the priming (several works characterized a positive feedback loop between apoptosis and JNK), but could not find a clear match between JNK activation and the priming of apoptosis in the notum. As highlighted above, we cannot monitor live GC3Ai following optoDronc activation. Still, as suggested we have now tested whether sublethal caspase activation is associated with DNA damage using γH2AX immunostaining (new **Fig. S5G**). While we could see

clear positive signal in late apoptotic debris (as expected), we could not find evidence of accumulation of positive signal in midline cells (showing positive GC3Ai signal while still being alive), which excludes DNA damage as a major driver of caspase sensitivity in the midline.

These results are now mentioned in the discussion line 448:

*"Caspase activity was also shown to trigger DNA damage[17, 18] which could sensitize cells for apoptosis over long timescales, however we could not detect any change of γH2AX spots in the midline despite positive GC3Ai signals, while late apoptotic cells were positively stained as expected(**Fig. S5G**)."*

4. The data shown in Figure 2E are inconsistent with results in Figure 2C. GC3Ai levels show negative correlation with caspase activity up to 6 hours prior cell extrusion (Fig. 2C). However, two pulses of caspase stimulation separated by 5 hours do not result in an increased % of extrusions.

Answer: It is correct that there is a slight inconsistency in the timescale observed in the correlation and the experiments with the pulse of optoDronc. There are two elements to consider here: first there is a significant notum to notum variability in the optoDronc experiment which may mask effects when they are more subtle (notably above 5 hours). In other words, there might still be a subtle impact, but the experiment is too noisy to reveal it. Secondly, the correlation shown in **2C**, as highlighted by the reviewer above, is just correlation. There could be other indirect effects that explain the longer timescale here. For instance, assuming that cells exposed to caspase 6 hours ago are more likely to be exposed again few hours later (since they are usually located in regions exposed to caspase activity throughout notum development), one would see a correlation extending beyond 3 hours even if the caspase activity taking place between 6 and 3 hours is not really influencing *per se* the final caspase/extrusion threshold. As rightfully pointed out by the reviewer, this correlation matrix is not a demonstration of the priming, but was an entry point to get an intuition of the parameters that may influence caspase threshold. The causality is really demonstrated by all the perturbative experiments performed from **Figure 2D**, **Figure 3** and **Figure 4**. We now openly discuss this inconsistency in the discussion of the paper line 355:

*"Of note, we also find a slight inconsistency between the timescale revealed by correlation (up to 6 hours, **Fig. 2C**) and the memory timescale revealed by optoDronc pulses (roughly 3 hours, **Fig. 2E**). Subtle effects may be persisting beyond 5 hours of delay and masked by the variability of the optoDronc experiment. Alternatively, the long-term correlation may be related to indirect effects (such as the probability to be exposed several times to caspase activity in a given domain of the tissue). Overall, these results match with a short-term memory of caspase activation lasting for few hours."*

5. The paper lacks comprehensive validation of the GC3Ai sensor's sensitivity and dynamic range in the specific context of the Drosophila pupal notum. Without specific validation, it's unclear whether the observed variability in caspase activity reflects biological differences.

Answer: It is a very relevant point. However, we would argue that the whole paper is a validation of the GC3Ai sensor's biological relevance since we do have strong predictive power coming from the GC3Ai derivative and relevant information provided by absolute GC3Ai signal (see **Fig. 1C**, **S1** and **4B,C**), reaching close to 80% of accurate prediction. This clearly argues for the relevant information provided by the derivative of the GC3Ai signal which so far is one of the best predictors of apoptosis close to the extrusion onset.

Currently the GC3Ai sensor is the only way we can quantitatively assess caspase activity at the single cell level *in vivo* and we don't have a way to correlate it at the single cell level with the proportion of active caspase (there is no tool to look at active caspase live). This cannot be worked around by biochemistry or single cell sorting either since we need to work with the time derivative of GC3Ai (requiring temporal dynamics and live recording). It is therefore very difficult to evaluate dynamic range *in vivo* (which by the way is difficult with all the live sensors currently used *in vivo*) in absence of alternative "ground truth".

While we cannot perform live imaging of GC3Ai combined with mild activation of optoDronc (see above), we can do ramp activation of optoDronc followed by a single time point measurement of GC3Ai 1 hour after activation (specifically in regions where there is no physiological caspase activity). We simultaneously exposed 4 regions in the notum to blue light with increasing pixel dwell time to modulate optoDronc activation, imaged E-cad::mKate to count extrusions and measured GC3Ai intensity from a single snapshot taken one hour after activation. This shows that we can tune the proportion of dying cells by changing blue light exposure (proving the tunability of optoDronc) and also increase GC3Ai signal proportionally. Note that GC3Ai intensity seems to saturate for the highest exposure values, most likely because most activated cells die before 60min for long exposure. This data is now presented in **Figure S3A-C** and described in the results line 169:

*"To assess the variability of caspase sensitivity in space, we used an optogenetic initiator caspase previously developed by our group (optoDronc[5]). optoDronc can be used to quantitatively modulate the level of caspase activation and the proportion of dying cells by modulating blue light exposure time (**Fig. S3A-C**). We therefore used global and short blue light exposure to trigger mild and uniform activation of caspases in a large band encompassing most of the notum (using the pnr-Gal4 driver, **Fig. 1F**)."*

6. The authors present a TSF model to classify dying vs. non-dying cells. However, the results partially conflict with the claim that past activation of GC3Ai primes cells for

death. TSF performance is lower than 60% for 1 hour before onset (Figure S1E). If past GC3Ai intensity is predictive of apoptosis, the model should be able to capture this or else raising doubts about the model. To clarify this, the authors should (1) compare to simpler baseline models (e.g., logistic regression or decision trees at individual time points) and (2) test performance on time-shuffled data. Moreover, key aspects of model development are missing. The authors should: (1) include cross-validation to assess generalizability; (2) conduct repeats for random sampling of non-dying cells to check model robustness; and (3) report 'specificity' to evaluate false positive rates, which complements their focus on identifying dying cells.

Answer: It is correct that TSF on raw GC3Ai does not show very high performance beyond a couple of hours. We would like to stress though that this is based here on raw and cell autonomous GC3Ai signal. As shown later in the paper, part of the decision is also non autonomous and depends on the relative signal with the neighbouring cells. When using relative GC3Ai signal to predict cell death among neighbours, the good guess score gets higher (>0.7 for more than 2h prior to extrusion).

Secondly, we would like to stress that our main claim is that <u>the dominant factor determining cell death remains the caspase activity just prior to extrusion initiation</u>. The <u>past exposure is a secondary parameter</u> that modulates the threshold but is not the dominant factor setting the death decision; otherwise, raw GC3Ai intensity alone would be predictive of death, which is contradicted by **Figure 1D** (where many cells with high raw intensity of GC3Ai are not dying). It is therefore not very surprising that TSF encompassing time windows which do not encapsulate caspase activity close to extrusion are not so strongly predictive by themselves and this matches our proposed model.

To complement this and as suggested by the reviewer, we have now tested prediction using logistic regression at individual time points and tested the performance of TSF with time-shuffled data (within given time windows prior to extrusion) to illustrate how much temporal dynamics are important for the prediction. Of note, this is not so different from what is represented in **Figure 1D** and **1E** (classifying all individual time points as "dead" or "alive" depending on the extrusion status of the cells). Interestingly, the logistic regression (LogReg) performed at single time points does not change drastically the prediction especially for caspase activity using absolute or relative values and guessing death among neighbours (as performed in **Fig. 4C**) (see **Figure R5B** below, compare with **R5A,** middle and bottom graphs). <u>This matches with the notion that caspase activity (GC3Ai derivative) is mostly relevant "instantaneously"</u>, and as such the predictive power of this parameter does not rely so much on temporal evolution. The situation is a bit different for raw GC3Ai where prediction is less accurate, especially less than 1 hour prior to extrusion, suggesting that the information provided by the dynamics in TSF (which includes the local slope of GC3Ai intensity variation) is here meaningful (F1 score >0.7 for TSF, ~0.55 for LogReg, **R5B** and **R5A top**). The fluctuations we observed with logistic regression also come from the single time points used for prediction. The same conclusion was reached by using <u>local</u>

temporal permutation of data within each time windows used in TSF approach and doing again TSF analysis (**Fig. R5C**). This does not greatly affect prediction with caspase activity, except for GC3Ai close to the extrusion engagement (F1 score<0.6), which is also reflected for the approach used to guess which neighbour will die through relative values (**Fig. R5A-C**, **bottom graphs**). Altogether, these analyses confirm our working model: namely that caspase activity close to extrusion is the most predictive factor which can be complemented by information from previous activity in the last 3-4 hours. Since this does not really change our conclusions we would prefer to stick to TSF which seems more reproducible and is actually the most recommended type of analysis for performing machine learning on temporal profiles.

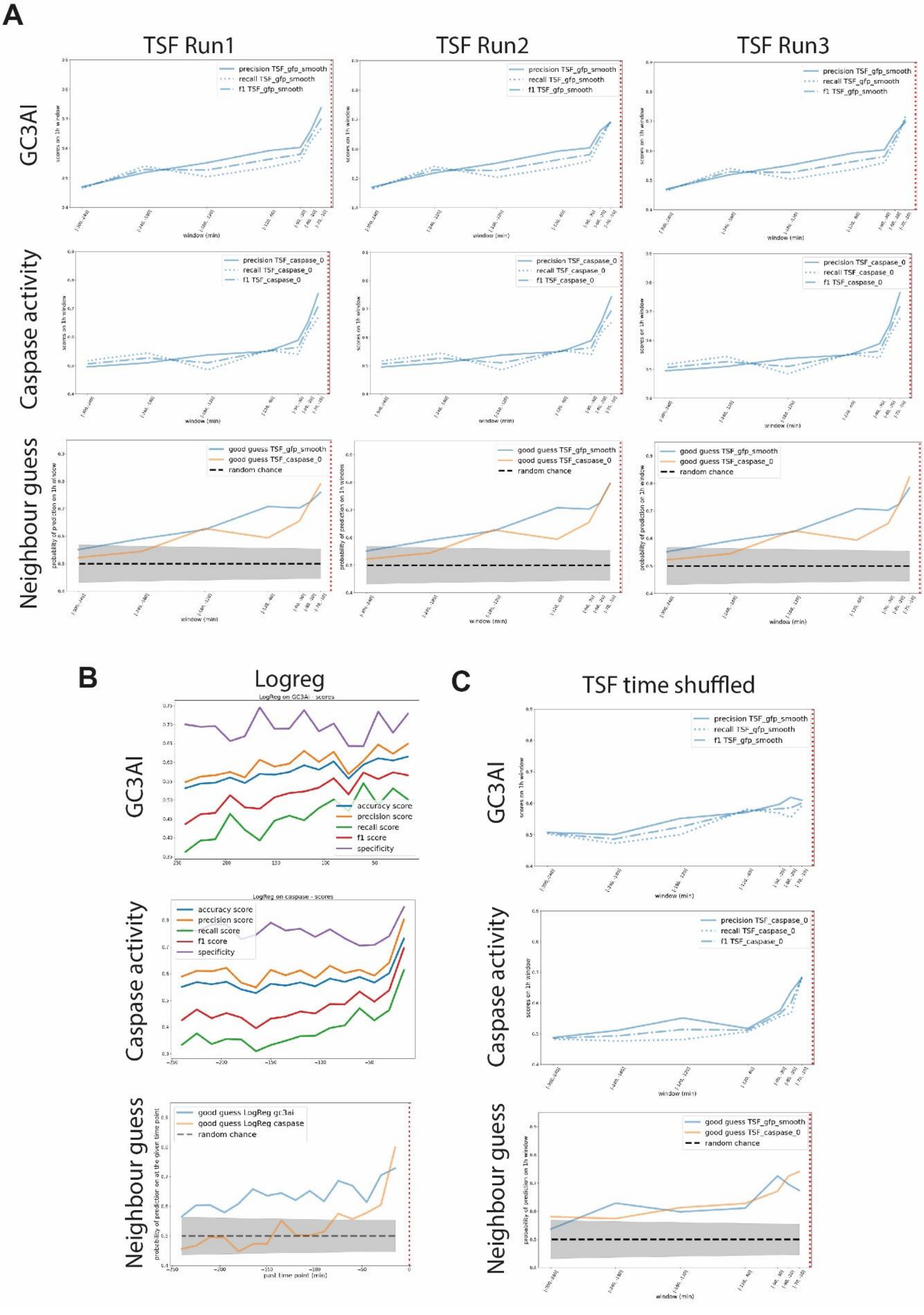

Figure R5: Contribution of time information in TSF and robustness. A: F1 score, precision and recall for predicting cell death on a given time windows through TSF using GC3Ai intensity (top), caspase activity (middle) or guessing which neighbour will die based on relative GC3Ai or relative caspase activity (bottom, see **Fig. 4C**). Each column shows one independent run

(random picking of one neighbour for training and for prediction), showing very subtle variations, illustrating the reproducibility of the TSF predictions. **B:** F1 score, precision, recall and specificity for predicting cell death through logistic regression (LogReg) using single time point of GC3Ai intensity (top), caspase activity (middle) or guessing which neighbour will die based on relative GC3Ai or relative caspase activity (bottom). **C:** F1 score, precision and recall for predicting cell death through TSF after randomization of time point within each time window of analysis, using GC3Ai intensity (top), caspase activity (middle) or guessing which neighbour will die based on relative GC3Ai or relative caspase activity (bottom).

Regarding cross-validation, in our understanding cross-validation (CV) is used for tuning hyperparameters and model evaluation. However, random forest algorithm (including TSF) has out-of-bag (OOB) error estimate which can be used instead of CV. By construction, random forest uses bootstrapping method - creates subsets on training datasets and trains one tree on each subset. The final prediction is created by aggregating all predictions. For every subset there are some missing data in the training that are then used for model validation, also called out-of-bag validation. Out-of-bag score is evaluated by aggregating predictions for all samples from the trees that did not use them in the training. We did use OOB error estimate to tune the number of estimators for our random forest model, and we believe that this estimate is sufficient to guarantee model generalisation. We provide now more details on this in the methods, line 986:

"Of note, random forest algorithm (including TSF) has out-of-bag (OOB) error estimate which can be used instead of cross-validation. By construction, random forest uses bootstrapping method - creates subsets on training datasets and trains one tree on each subset. The final prediction is created by aggregating all predictions. For every subset there are some missing data in the training that are then used for model validation, also called out-of-bag validation. Out-of-bag score is evaluated by aggregating predictions for all samples from the trees that did not use them in the training "

As for random sampling of neighbours, we would like first to clarify some of our choices. We voluntarily use neigbouring cells as controls to train the TSF as picking up more distant surviving cells as controls generates a lot of artefacts coming from the very fine patterning of the tissue. For instance, we previously used machine learning to test the impact of cell morphology/mechanics on cell death prediction and found very strong F1 score up to several hours before death. However, this appeared to be artefactual and mostly reflecting the "patterning" of cell shape in the tissue (in other words, cells in regions of high death rate also happen to have a different shape without causal link between shape and death). When the training used instead direct dying cell neighbours as control, the F1 score was drastically reduced. We just want to stress here that we probably use the most stringent/challenging prediction test by restricting it to direct neighbours of dying cells (where everything should be the same in terms of patterning or developmental time). We believe the predictive scores we reach, while not perfect, are already quite satisfactory.

Still, we complement the TSF by documenting the robustness of the prediction using several rounds of random sampling of dying cell neighbours as controls to illustrate the robustness. This is provided above in **Figure R5A**, showing the very subtle changes between each run. We mention this validation in the method now line 1101:

"We validated the reproducibility of the prediction compared three independent TSF predictions (showing almost no variation in the F1 score at all time windows)"

Finally, regarding specificity, we did provide in **Figure S1** the curve of the recall and precision for all the parameters, which are already providing the point the referee is looking for (evaluation of false positive rate with the precision which is TP/(TP+FP), evaluation of the false negative rate with the recall which is equal to TP/(TP+FN)). We clarified this in the legend. If the referee thinks it is essential, we can also calculate the specificity (negative prediction/TN) but for the moment we thought it was not absolutely required to display it (especially on figures that are already quite heavy).

7. The term "probability" is used inconsistently throughout the manuscript, referring at different sections to the proportion of dying cells, probability from the TSF model or logistic regression, or death rates. The authors should use method-specific terminology.

Answer: It is a good point and we apologise if this was confusing. We now use a specific terminology for each methodology while trying to preserve the accessibility of the article to a wide readership.

Reviewer #3 (Remarks to the Author):

I co-reviewed this manuscript with one of the reviewers who provided the listed reports. This is part of the Nature Communications initiative to facilitate training in peer review and to provide appropriate recognition for Early Career Researchers who co-review manuscripts.

terminology.

Answer: Thanks for taking time to assess our work. We hope our answers will look satisfactory.

Reviewer #4 (Remarks to the Author):

Summary

The manuscript of Cumming et al. presents an intriguing hypothesis that past caspase activity in otherwise viable cells, biases the induction of cell death at future timepoints in vivo. This manuscript provides a critical test of the caspase activity threshold and

demonstrates that activity alone is not a sole arbiter of apoptosis, rather is a factor along with past activity itself, which primes developing cells for eventually apoptotic/programmed cell death. This manuscript makes use of state of the art imaging and real time reporter systems in an in vivo setting (drosophila) to establish the role of caspase activity 'history' as a death determinant, a set of observations that will be compelling to a large number of cell and developmental biologists, as well as those biochemists that are interested in the temporal control over caspase activity and cell death onset.

The GC3Ai sensor combined with machine learning is a powerful method to track in vivo caspase activity exposure. The curation of caspase activity bursts using this approach is a major advance over prior protease activity assays. Figure 1E shows a relatively narrow band of death 'probability' which fits with the tightly regulated process of apoptosis, which is further validation for the assay. The use of extrusion as a proxy for late stage death is also a valid measure and aligns quite well with reporter activity across a subset of the caspase activated cell population. Moreover, the real time videos provide compelling data support for the hypothesis as articulated by the authors. Other individual experiments are quite compelling, for example the data within Figure 3A and 3B provide strong support for past caspase activation and eventual cell death (although 3B could be buttressed by more supporting observations, see below). Figure 4 is equally compelling in terms of 'neighbor' effects. Prior to publication the authors should address a number of points as noted below.

Answer: We would like to thank the reviewer for the very positive feedback and the appreciation of our work.

Corrections

Regarding the abstract. The abstract is somewhat obtuse when explaining the dynamics of caspase activity and cell death decision making. The authors introduce the threshold concept, yet the abstract walks the reader away from this notion entirely. Despite the focus on past sublethal capase activation as the bias for cell death/elimination, the authors data in Figure 4A,B in fact suggest that a caspase activity threshold may exist along with past exposure bias. Given this issue the authors should acknowledge this nuance in the abstract, as the current version indicates to the reader that activity thresholds are not a contingent factor at all.

Answer: This is a very valid point. It is true that we emphasize a lot the history part which seemed more novel, but down the line there is still a death threshold at the individual cell level. This is just more heterogeneous than what we initially expected. We have revised the manuscript abstract to better reflect this nuance as well as the discussion by first replacing the term sensitivity with threshold (to make clear that we

do not deny the existence of single cell threshold) and better reflect the combinatorial role of past caspase and current caspase activity for cell death decision.

Regarding comments in the introduction. The authors state that nonapoptotic links between MOMP, mitochondrial activation of caspase cascades is a recent phenomenon over the past decade, linked largely to caspase 3 activity in resistant cancer cells. The authors should actually acknowledge the work of the labs that predate these observations considerably which include the following papers showing nonapoptotic outcomes for mitochondrial involvement in nonapoptotic caspase function across a number of cell phenomenon, i.e. Arama et al 2003, Weber and Menko 2005, Putinski et al. 2013.

Answer: These are indeed very relevant references which already documented non-apoptotic function of caspases a while ago. There is indeed a large literature on the topic and we didn't try to be exhaustive. It is however very relevant to mention the pioneer works showing these non-apoptotic functions in vivo and we now properly quote these references in the introduction line 66:

"Similarly, mitochondrial outer membrane permeabilisation (MOMP), which was long thought to be an all-or-nothing process systematically leading to apoptosis[19, 20], was shown to be more nuanced due to the occurrence of partial MOMP that does not lead to cell death both in vivo[21-23] and in cell culture[17, 24]. "

Regarding Supp Figure 1J. The aligned image does not appear to highlight an area of measurable caspase activity or an intact cell per se. The authors should provide images that align with the modeling predictions.

Answer: The **Supp Fig 1J** shows a dying cell where GC3Ai signal appears quite late compared to other examples. Of note, the nuclear signal appears offset as cells are always a bit tilted along apico-basal axis in the notum. We now provide another example that will better illustrate the analysis, we hope it will be clearer now.

Regarding Figure 1. While I do agree that the gross variability is caspase activity is not predictive, this does not exclude the possibility that variation in regional caspase activity may be determinative, i.e. subcellular regions where protease activity is a threshold signal for cell fate/apoptosis. That is not really addressed in the manuscript. Given the novelty of the observations I am of the opinion that this should be addressed in future research efforts, however the authors should temper their interpretations somewhat or acknowledge this issue, perhaps in the discussion.

Answer: This is a very valid point and it is true that the GC3Ai sensor only gives us access to bulk caspase activity without subcellular resolution. We cannot exclude at this stage that a more clearly defined threshold may exist in subcompartments of the cell. We now mention this opening in the discussion more clearly. In fact, one key element missing here is the development of a tool allowing live assessment of subcellular caspase activity. We tried in the past to build a FRET-based effector caspase sensor (looking directly at the 'active' caspase, and not a downstream

substrate cleavage) but that was not successful so far. This would be a game changer for addressing this question. This point is now mentioned in discussion line 349:

"While our work outlined an important cell-to-cell heterogeneity in the caspase activity levels at which cells engage in apoptosis, it does not exclude the existence of a relevant single cell threshold for apoptosis induction. Moreover, our characterisation was based on a bulk sensor of caspase activity, and we cannot exclude that the apoptotic decision relies on a specific subcellular pool of active caspase where a well-defined deterministic threshold may still exist."

Regarding Figure 2D,E. This data is essentially the heart of the argument, i.e. the authors suggest that the use of the optoDronc activatable construct is consistent with recent past caspase activity being determinant for death. At this stage the variability in the data in Figure 2E is suggestive but certainly not definitive to support the 'past activity means death' hypothesis. Given the variability in the dataset, and to support the core argument the authors should provide time points on either side of the 1-5 hour window, 30 mins past activation 10 hours past activation. If the hypothesis is correct one would anticipate a dramatic difference in the bar graphs from what is observed in Figure 2D,E.

Answer: Thanks for pointing this out. As the reviewer noted, the experiment in **Fig. 2D,E** is quite variable and there is an inherent variability of the outcome of optoDronc activation from pupa to pupa (probably coming from differences in the levels of expression and/or the inherent variability of optogenetics). That is one of the very reasons why we systematically used an internal control. It is a very good suggestion to complement this experiment with more time points. We now provide the same quantification 30min or 8h post activation. Of note, we initially avoided those for two reasons: 30min is a very short time window and we could capture extrusions coming from the first pulse (since extrusion takes on average 20min). Secondly, we cannot really neglect anymore the impact of developmental progression after 10h which may change the response of the tissue after optoDronc activation. Having said that, these results should complement our estimation of the timescale of the memory. We have performed those experiments and added them in the manuscript in **Fig. 2E**. We observed as expected a high bias at 30min, similar to 1h, and no more bias after 8h as observed after 5h.

Regarding Figure 3B,C, I do agree with the authors that the data is generally supportive of the hypothesis, however the limitation here is a single point in time with the hid-RNAi experiment. Why did the authors opt for a late stage interference experiment and not an early experiment as well at 18hrs? An earlier ablation of hid would strengthen the conclusions to a significant degree. The data in Figure 3D,E may actually suffice for this request, however it is not clear at what point the combined optoDronc and GC3Ai experiment was initiated in the developmental window? Some clarity here could diminish the need for another timepoint as noted above. A slightly modified version of

movie S8 would also be helpful here (slower track) to visualize the overlay in signals which move to quickly.

Answer: This is a relevant point. We initially focused on later time point for two reasons: they are actually way easier to image/mount (especially in the hid RNAi background where the defective fusion of the heminota very often generates tissue folding at early stages which makes the optogenetic experiment and imaging very difficult, this folding is usually resolved at later developmental time), and also because we wanted to start from conditions showing the highest death rate in controls. To be clear and since the reviewer mentions *"earlier ablation of hid"*, in all the experiment hid is depleted at the same time (throughout development in the pnr domain), the only temporality changing is the time of activation of optoDronc. As highlighted above, while we agree that obtaining maps on earlier time points could be interesting, we would prefer to avoid this experiment which will be very difficult (we already tried but most of the tissues were highly folded in the hid RNAi background at this stage, preventing proper imaging and optoDronc activation). We hope the reviewer will understand.

Fig 3D,E was done on early pupae (18h APF), but did not focus on the global pattern and didn't use hid RNAi, we now make this more clear in the legend and main text. We also provide as suggested a modified version of **movie S8** showing on the top the still image of GC3Ai signal at T0 to facilitate the comparison with the tracking of dying cells. Note though that the GC3Ai image is tricky to obtain as we use the "pulse" of blue light used to activate optoDronc to image a snapshot of GC3Ai (doing more would start to excessively activate Dronc and kill all the cells).

Regarding Suppl Figure 3. The authors should consider placing this as a main figure (stand alone figure) as this is strong support for the generality of their model (another cell type) regarding caspase induced death and susceptibility to past caspase activity.

Answer: This is a good suggestion. We initially thought that this would better fit in the discussion as this is more of an opening (and we didn't characterize exhaustively how this is working in the wing disc), but we now include it in the main figure as suggested in a new **Figure 5B**. Showing the generality of the model is indeed important.

Regarding Suppl Figure 4. I do not understand the rationale for including this data. All of the assays and their targets do not appear to modify the past caspase activity and links to follow on death induction. Certainly a discussion of these targets is warranted by inclusion of a number of individual experiments do not provide definitive support or exclusion for these various modifiers. Indeed the authors themselves note that these candidates could operate in tandem or combination to effect the caspase activity sensitization step. Unless the authors want to actually address this issue directly with additional experimentation, they would be better served not presenting this preliminary data, which does not really allow for firm conclusions in its present state.

Answer: We thank the reviewer for this suggestion. We believe though that it is relevant to keep this data. It is important to document the fact that the most obvious

candidates for regulating this memory do not seem to explain the priming effect on their own. While these are mostly negative results, they are important to share if others want to explore the molecular nature of the memory (which we are also currently exploring in the lab). While the "n" of each observation is not huge, they really reflect the absence of an obvious link with the pattern of death and/or the impact of a sublethal optoDronc pulse. We would therefore prefer to keep this figure in the paper, especially since it is now not really possible to mention results in a paper without showing them.

References

1. Albeck, J.G. *et al*. Quantitative analysis of pathways controlling extrinsic apoptosis in single cells. *Mol Cell* **30**, 11-25 (2008).
2. Tawa, P. *et al*. Catalytic activity of caspase-3 is required for its degradation: stabilization of the active complex by synthetic inhibitors. *Cell Death Differ* **11**, 439-447 (2004).
3. Suzuki, Y., Nakabayashi, Y. & Takahashi, R. Ubiquitin-protein ligase activity of X-linked inhibitor of apoptosis protein promotes proteasomal degradation of caspase-3 and enhances its anti-apoptotic effect in Fas-induced cell death. *Proc Natl Acad Sci U S A* **98**, 8662-8667 (2001).
4. Choi, Y.E. *et al*. The E3 ubiquitin ligase cIAP1 binds and ubiquitinates caspase-3 and -7 via unique mechanisms at distinct steps in their processing. *J Biol Chem* **284**, 12772-12782 (2009).
5. Valon, L. *et al*. Robustness of epithelial sealing is an emerging property of local ERK feedback driven by cell elimination. *Dev Cell* **56**, 1700-1711 e1708 (2021).
6. Zhang, J. *et al*. Visualization of caspase-3-like activity in cells using a genetically encoded fluorescent biosensor activated by protein cleavage. *Nat Commun* **4**, 2157 (2013).
7. Nakajima, Y.I. & Kuranaga, E. Caspase-dependent non-apoptotic processes in development. *Cell Death Differ* **24**, 1422-1430 (2017).
8. Fujisawa, Y., Kosakamoto, H., Chihara, T. & Miura, M. Non-apoptotic function of Drosophila caspase activation in epithelial thorax closure and wound healing. *Development* **146** (2019).
9. Mann, Z. *et al*. Preexisting tissue mechanical hypertension at adherens junctions disrupts apoptotic extrusion in epithelia. *Mol Biol Cell* **35**, br3 (2024).
10. Guirao, B. *et al*. Unified quantitative characterization of epithelial tissue development. *Elife* **4** (2015).
11. Ryoo, H.D., Gorenc, T. & Steller, H. Apoptotic cells can induce compensatory cell proliferation through the JNK and the Wingless signaling pathways. *Dev Cell* **7**, 491-501 (2004).
12. Shlevkov, E. & Morata, G. A dp53/JNK-dependant feedback amplification loop is essential for the apoptotic response to stress in Drosophila. *Cell Death Differ* **19**, 451-460 (2012).
13. Chatterjee, N. & Bohmann, D. A versatile PhiC31 based reporter system for measuring AP-1 and Nrf2 signaling in Drosophila and in tissue culture. *PLoS One* **7**, e34063 (2012).

14. Fan, Y. & Bergmann, A. Multiple mechanisms modulate distinct cellular susceptibilities toward apoptosis in the developing Drosophila eye. *Dev Cell* **30**, 48-60 (2014).
15. Koto, A., Kuranaga, E. & Miura, M. Temporal regulation of Drosophila IAP1 determines caspase functions in sensory organ development. *J Cell Biol* **187**, 219-231 (2009).
16. Huang, Q. *et al.* Ubr3 E3 ligase regulates apoptosis by controlling the activity of DIAP1 in Drosophila. *Cell Death Differ* **21**, 1961-1970 (2014).
17. Ichim, G. *et al.* Limited Mitochondrial Permeabilization Causes DNA Damage and Genomic Instability in the Absence of Cell Death. *Molecular Cell* **57**, 860-860 (2015).
18. Sakahira, H., Enari, M. & Nagata, S. Cleavage of CAD inhibitor in CAD activation and DNA degradation during apoptosis. *Nature* **391**, 96-99 (1998).
19. Goldstein, J.C., Waterhouse, N.J., Juin, P., Evan, G.I. & Green, D.R. The coordinate release of cytochrome c during apoptosis is rapid, complete and kinetically invariant. *Nat Cell Biol* **2**, 156-162 (2000).
20. Rehm, M., Dussmann, H. & Prehn, J.H. Real-time single cell analysis of Smac/DIABLO release during apoptosis. *J Cell Biol* **162**, 1031-1043 (2003).
21. Arama, E., Agapite, J. & Steller, H. Caspase activity and a specific cytochrome c are required for sperm differentiation in. *Developmental Cell* **4**, 687-697 (2003).
22. Weber, G.F. & Menko, A.S. The canonical intrinsic mitochondrial death pathway has a non-apoptotic role in signaling lens cell differentiation. *Journal of Biological Chemistry* **280**, 22135-22145 (2005).
23. Putinski, C. *et al.* Intrinsic-mediated caspase activation is essential for cardiomyocyte hypertrophy. *Proceedings of the National Academy of Sciences of the United States of America* **110**, E4079-E4087 (2013).
24. Tait, S.W.G. *et al.* Resistance to caspase-independent cell death requires persistence of intact mitochondria. *Developmental cell* **18**, 802-802 (2010).

Point by point answer to reviewers.

Integration of past caspase activity biases cell elimination *in vivo*

General answer:

We would like to thank the reviewers for their constructive comments and suggestions as well as the appreciation of the novelty of our work. We have addressed the last points raised by reviewer 1 by adding two set of experiments which clearly suggest that:

- Diap1 overexpression can modulate death engagement downstream of Dronc activation (as previously suggested in previously published works)
- However Diap1 modulation does not seem to drastically change the effector caspase activity threshold at which cells engage in apoptosis.

Those are two additional piece of data that help to position Diap1 in the regulation of caspase threshold associated with cell death and we believe they are a nice addition in the manuscript. We hope the reviewer will find that these two last additions properly address their last concern.

Point by point answers

All the answers can be found in blue.

Reviewer #1 (Remarks to the Author):

The revised manuscript has clearly improved. The authors have added new experimental data, provided better quantifications of timing and reversibility of caspase priming, improved the validation of the GC3Ai sensor, and also added additional negative data (DNA damage, ROS, protasome activity, cytoskeleton) that strengthen the conclusion that priming is not simply explained by known pathways. These revisions meaningfully strengthen the central claims and address several concerns raised during review.

However, regarding the last point raised in my first review, I appreciate the efforts of the authors to address my comment regarding a potential role of Diap1 in heterogeneity of the apoptosis threshold, including the generation and analysis of the Diap1::mScarlet knock-in line. These data provide useful insight into the spatial patterning of Diap1 protein levels in the pupal notum and clarify that Diap1 does not appear to encode the short-term memory of past caspase activity. However, my original point was that cell-intrinsic differences in Diap1 protein levels could contribute to the heterogeneous caspase thresholds observed across epithelial cells, rather than to caspase memory per se. While the current data are correlative, a direct test of causality would require experimental manipulation of Diap1 levels. I therefore encourage the authors to consider reducing Diap1 dose heterozygously and, conversely, overexpressing Diap1 to directly assess whether Diap1 dosage modulates the caspase threshold for apoptosis in the pupa notum.

Answer: We thank the reviewer for the positive feedback and for raising this important point. We initially wanted to focus mostly on the role of Diap1 in the short term memory which is the main subject of this article, but we do agree that further evaluating how Diap1 cell intrinsic differences may alter caspase threshold is indeed a very relevant question, though we already documented that the endogenous pattern only partially explain the spatial pattern of death induction upon optoDronc activation. To address this last suggestion, we have performed two additional sets of experiments:

- We have tracked and compared the caspase threshold within the same tissue of WT cells and clonal cells where Diap1 was depleted by RNAi. Luckily, 48h induction of Diap1 RNAi in clones generates "hypomorphic" like conditions whereby clonal cells still survive, while having a higher probability to activate caspases and die, even ectopically (see new **Figure S6G**, compare top side in the clone and the symmetric WT part, new **movie S17**). Note that we assess caspase activity with a ubiquitous lexA driven GC3Ai sensor where signal is not as good as the Gal4 driven sensor. Still, we tracked a number of dying cells in the clone and in the WT cells and compared the level of caspase activity at the onset. We could not find a significant difference in the caspase threshold associated with the onset of extrusion (there is even a tendency to see slightly higher threshold in the clones, see new **Figure S6H**).
- We have assessed cell death upon mild optoDronc induction throughout the notum upon overexpression of Diap1 in the pnr-gal4 domain. Interestingly, Diap1 overexpression appears to drastically reduce the number of cell death induced upon mild Dronc activation (see new **Figure S6 I-K, movie S18**).

Altogether, these results confirm that Diap1 overexpression can modulate death induction downstream of Dronc, most likely through direct modulation of effector caspase activity as previously demonstrated (Li *et al.*, Nat Comm. 2011). However this also suggests (with the limit of the lexA driven sensor sensitivity) that Diap1 does not drastically modulate the effector caspase activity threshold at which cells engage in extrusion. These results are in line with previous publications (Fan and Bergman, *Dev Cell* 2014 or Koto *et al.* JCB 2009) which mostly documented a role of Diap1 levels in the reduction of sensitivity to pro-apoptotic signal (mostly *hid* or *reaper* expression) where Diap1 also modulates downstream effector caspase activation, but did not necessarily document the very downstream relationship between effector caspase activity and death engagement upon Diap1 modulation. We believe those results bring an interesting clarification, while we remain cautious on the fact that we may not be able to detect subtle change of caspase threshold with our tool. We now describe these new results in the discussion of the latest version of the article line 446:

*"Accordingly, while Diap1 RNAi was sufficient to trigger ectopic caspase activation in clones (**Fig. S6G**, **movie S17**), it did not change significantly the effector caspase activity threshold at which cells engage in apoptosis (**Fig. S6H**) suggesting that Diap1 levels are unlikely to change effector caspase sensitivity (with the limit of the sensitivity*

*of our sensor). However, in good agreement with previous results showing that Diap1 levels modulate sensitivity to pro-apoptotic signals[1, 2] and that Diap1 can modulate effector caspase activity downstream of Dronc[3], we found that Diap1 overexpression in the notum drastically reduces the number of cell death triggered by a pulse of optoDronc activation (**Fig. S6I-K, movie S18**). Altogether, these results confirm that Diap1 can modulate apoptosis entry downstream of Dronc, but is unlikely to modulate effector caspase activity threshold at which cells engage in apoptosis and to explain the priming."*

Reviewer #2 (Remarks to the Author):

We appreciate and acknowledge the authors' detailed responses, particularly their clarification of the system's limitations and how these may have constrained the measurements they were able to perform. Based on the revised manuscript, we believe the results largely support their conclusions, and we are supportive of the publication of their work.

Answer: We thank the reviewer for their very positive feedback and their support.

Reviewer #3 (Remarks to the Author):

I co-reviewed this manuscript with one of the reviewers who provided the listed reports. This is part of the Nature Communications initiative to facilitate training in peer review and to provide appropriate recognition for Early Career Researchers who co-review manuscripts.

Answer: We thank the reviewer for engaging time in evaluating our work.

Reviewer #4 (Remarks to the Author):

The authors have provided an extensive revision addressing my main points of concern. The additional experiments/data largely support the core hypothesis, and I anticipate that the field will benefit from the publication of this work.

Answer: We thank the reviewer for the very supportive feedback and the evaluation of the impact of our work.

Rebutal references:

1. Koto, A., Kuranaga, E. & Miura, M. Temporal regulation of Drosophila IAP1 determines caspase functions in sensory organ development. *J Cell Biol* **187**, 219-231 (2009).
2. Fan, Y. & Bergmann, A. Multiple mechanisms modulate distinct cellular susceptibilities toward apoptosis in the developing Drosophila eye. *Dev Cell* **30**, 48-60 (2014).

3. Li, X., Wang, J. & Shi, Y. Structural mechanisms of DIAP1 auto-inhibition and DIAP1-mediated inhibition of drICE. *Nat Commun* **2**, 408 (2011).
