# Supplementary material for: Short-term memory of past caspase activity biases cell elimination in vivo
Source: Nat Commun. 2026 Sep 22;17:11031. doi: 10.1038/s41467-026-77685-9 (PMC13687027; doi:10.1038/s41467-026-77685-9)
Supplement: Supplementary file 4 [file 41467_2026_77685_MOESM2_ESM.pdf]

Description of Additional Supplementary Files

Supplementary Movie 1: Movie related to **Fig. 1A-E, 2A-C, 4A-C, S1A-I**. ~16h APF (After Pupae Formation) notum local z-projection with E-cad::mKate (top left, magenta) and UAS-GC3Ai (top middle, green) driven by the pnr-Gal4 driver. Bottom left shows the skeletonised tissue. Bottom middle the cell tracking (1 colour per cell), the bottom right the colour coded cell average GC3Ai intensity (dark blue low, yellow high). Anterior, left, posterior, right. Scale bar is 50μm.

Supplementary Movie 2: Movie related to **Fig. 1C**. Example of an extruding cell from a notum local z-projection with E-cad::mKate (middle, magenta) and UAS-GC3Ai (right, green) driven by the pnr-Gal4 driver. The skeletonised contour of cells is shown on the left, the dying cell is shown in white and with yellow contours. Anterior, left, posterior, right. Scale bar is 5μm.

Supplementary Movie 3: Movie related to **Fig. 1F,J**. ~20h APF notum local z-projection with E-cad::mKate (greyscale) expressing UAS-optoDronc::GFP driven by the pnr-Gal4 driver after global mild activation of optoDronc (left) or without blue light activation (control, right). Red dots show the dying cells. Anterior, left, posterior, right. Scale bars are 50μm.

Supplementary Movie 4: Movie related to **Fig. S3F,G**. ~20h APF notum local z-projection with E-cad::mKate (left, magenta) expressing UAS-optoDronc and UAS-GC3Ai (middle, green) driven by the pnr-Gal4 during a strong and global activation of optoDronc. Anterior, left, posterior, right. Scale bar is 50μm.

Supplementary Movie 5: Movie related to **Fig. 2D**. ~20h APF notum local z-projection with E-cad::mKate (greyscale) expressing UAS-optoDronc::GFP driven by the pnr-Gal4 after a local activation of optoDronc (in the red and blue square regions). The blue square was already exposed to a pulse of blue light 1 hour before. Dying cells in the regions of interest are shown with red dots. Anterior, left, posterior, right. Scale bar is 50μm.

Supplementary Movie 6: Movie related to **Fig. 3A**. Nota local z-projection with E-cad::mKate (greyscale) expressing UAS-optoDronc::GFP driven by the pnr-Gal4 driver after global mild activation of optoDronc in a young pupae (left, 18h APF), late pupae (middle, 24h APF) or upon inhibition of *hid* (*UAS-hid* dsRNA, right). Red dots show the dying cells. Anterior, left, posterior, right. Scale bars are 50μm.

Supplementary Movie 7: Movie related to **Fig. 3B**. ~16h APF notum local z-projection with E-cad::mKate (left, magenta) expressing UAS-GC3Ai (middle, green) and UAS-hid dsRNA driven by the pnr-Gal4 driver. Compare with **Movie S1** for control. Anterior, left, posterior, right. Scale bar is 50μm.

Supplementary Movie 8: Movie related to **Fig. 3D,E**. ~16h APF notum local z-projection with E-cad::mKate (magenta) expressing UAS-GC3Ai (green) and UAS-optoDronc driven by the pnr-Gal4 driver after global mild activation of optoDronc (also used to image GC3Ai at T0). Red dots show the dying cells. The top panel show the still image of the GC3Ai signal at time 0. Anterior, left, posterior, right. Scale bar is 20μm.

Supplementary Movie 9: Movie related to **Fig. 4D**. ~24h APF notum local z-projection with E-cad::mKate (left, magenta), UAS-optoDronc::GFP and UAS-mCd4::mIFP-T2A-HO1 (green, middle) expressed in clones after mild activation of optoDronc. Red dots show the dying cells detected between 1.5hrs and 5hrs after the blue light pulse. Anterior, left, posterior, right. Scale bar is 30μm.

Supplementary Movie 10: Movie related to **Supp. Fig. 5A**. ~18h APF notum local z-projection with E-cad::mKate (left, green) and the JNK pathway sensor TRE-RFP (middle, magenta). Anterior, left, posterior, right. Scale bar is 50μm.

Supplementary Movie 11: Movie related to **Supp. Fig. 5B**. ~18h APF notum local z-projection with E-cad::mKate (left, green) and the ROS sensor GstD-GFP (middle, magenta). Anterior, left, posterior, right. Scale bar is 50μm.

Supplementary Movie 12: Movie related to **Supp. Fig. 5D**. ~16h APF notum local z-projection with E-cad::mKate (left, green) and the proteasome activity sensor Proteoflux (hs-CL1::GFP, middle, magenta) levels after a pulse of transcriptional activation. Anterior, left, posterior, right. Scale bar is 50μm.

Supplementary Movie 13: Movie related to **Supp. Fig. 5D**. ~20h APF notum local z-projection with sqh::mKate (greyscale) and UAS-optoDronc::GFP driven by the pnr-Gal4 driver after mild blue light stimulation in the blue square region. Anterior, left, posterior, right. Scale bar is 50μm.

Supplementary Movie 14: Movie related to **Supp. Fig. 5F**. ~20h APF notum local z-projection with UAS-αtub::mCherry (greyscale) and UAS-optoDronc::GFP driven by the pnr-Gal4 driver after mild blue light stimulation in the blue square region. Anterior, left, posterior, right. Scale bar is 50μm.

Supplementary Movie 15: Movie related to **Supp. Fig. 6A**. ~16h APF notum local z-projection with E-cad::GFP (green, left) and Diap1::mScarlet (KI) (magenta, middle). Anterior, left, posterior, right. Scale bar is 50μm.

Supplementary Movie 16: Movie related to **Supp. Fig. 6B**. ~20h APF notum local z-projection expressing UAS-optoDronc::GFP and UAS-mCd4::mIFP-T2A-HO1 (green, left) and Diap1::mScarlet (KI) (magenta, middle) with the pnr-Gal4 driver after mild activation of optoDronc in the blue square region. Anterior, left, posterior, right. Scale bar is 50μm.

Supplementary Movie 17: Movie related to **Supp. Fig. 6G-H**. ~24h APF notum local z-projection with E-cad::mKate (left, magenta), UAS-mCd4::mIFP-T2A-HO1 expressed in clones where Diap1 was depleted by RNAi (grey, middle) and ubiquitous expression of GC3Ai (act-lexA, lexAOP-GC3Ai, right, green). Anterior, left, posterior, right. Scale bar is 50 μm.

Supplementary Movie 18: Movie related to **Supp. Fig. 6I-K**. Notum local z-projection with E-cad::mKate (greyscale) expressing UAS-optoDronc::GFP and UAS-diap1 driven by the pnr-Gal4 driver after global mild activation of optoDronc (seen in green during the first time frames,18h APF). Not cell death is observed. Anterior, left, posterior, right. Scale bar is 50μm.
